# The association between flavor capsule cigarette use and sociodemographic variables: Evidence from Chile

**DOI:** 10.1371/journal.pone.0224217

**Published:** 2019-10-23

**Authors:** Guillermo Paraje, Daniel Araya, Jeffrey Drope

**Affiliations:** 1 Escuela de Negocios, Universidad Adolfo Ibáñez, Santiago de Chile, Chile; 2 Economic & Health Policy Research, American Cancer Society, Atlanta, Georgia, United States of America; 3 Department of Political Science, Marquette University, Atlanta, Georgia, United States of America; Medical University of South Carolina, UNITED STATES

## Abstract

**Purpose:**

The objective of this article is to examine the factors associated with smoking of flavor capsule cigarettes in Chile, where the popularity of these products has increased dramatically, a trend increasingly observed across the world.

**Methods:**

A representative poll of 851 smokers in Metropolitan Santiago de Chile, which comprises 40% of the country's total population, was implemented in mid-2017. Smokers were given a questionnaire that collected socio-demographic information and information on smoking patterns. Four discrete-choice models were estimated on the decision to smoke flavor capsule cigarettes to better understand the statistical relationships between traits of smokers and the consumption of flavor capsule cigarettes.

**Results:**

The results of these models show that each year less in a smoker’s age increases the likelihood of preferring flavor capsule cigarettes by, on average, between 0.8 and 0.9 percentage points. If the smoker is a woman, the likelihood of preferring flavor capsule cigarettes increases between 13.4 and 13.5 percentage points. Results also reveal a positive relationship between the price paid and the consumption of flavor capsule cigarettes, indicating that these cigarettes tend to be more expensive. There is no statistical relationship between participation in the labor market and smoking these products.

**Conclusions:**

Chile has the world’s highest prevalence of flavor capsule cigarette smoking, which is concentrated among young people (25 years and younger) and females. No relationship between socioeconomic status and use of these products is found, though there are indications that such relationship may exist, as they were at the time of study 14% more expensive, on average, than conventional non-flavored cigarettes. As in most countries, the tobacco industry appears to be deliberately promoting these products with the goal of halting or slowing the decline in cigarette consumption in Chile. Thus, to reduce cigarette consumption (especially among youth), restricting or forbidding cigarette flavorings of all types, including flavor capsules, would be an effective strategy.

## Introduction

Flavors for cigarettes have been used frequently and consistently by the tobacco industry since the beginning of the 20^th^ Century. Menthol is the most common flavoring, with a global market share of about 10% [1]. Its "cooling" and anesthetic properties are used, among other things, to reduce the irritation caused by the inhalation of cigarette smoke, the initial displeasure of smoking among new, usually young, smokers and the fact that these products are frequently perceived as less harmful than regular cigarettes [1–3]. From as early as 1933, flavored cigarettes were marketed as a “remedy to the burn, dryness and throat irritation that accompany smoking”, and to reinforce “identity” and “in-group belonging” in specific groups, such as African-Americans, women and youth in the USA [2]. Apart from the appealing features of menthol, there is also evidence that it increases the addictive properties of nicotine [4] and the toxicity of cigarettes [5].

Flavored cigarettes are an increasing concern for global public health, as their use is growing rapidly [1, 6]. Flavoring is defined as “additive that imparts smell and/or taste”, while additive is defined as “a substance, other than tobacco, that is added to a tobacco product, a unit packet or to any outside packaging” [6]. Consumer data from 2019 show that at least 18 countries (out of an available 78) have a market share for flavored cigarettes exceeding 20% [7]. Seven of these are high-income countries. Although countries with large flavored cigarette market segments are spread across the globe, six (Peru, Guatemala, Chile, Dominican Republic, Mexico and Colombia) are in Latin America and flavored cigarette use is spreading rapidly among the region’s remaining countries [8]. Within the flavored cigarette segment, the fastest growing sub-segment is the flavor capsule. These products, an innovation of the tobacco industry introduced as early as 2008, but strongly marketed after 2011 [7], consists of a flavor capsule (or more than one) that is located in the filter. If the smoker decides to press filter in order to crush the capsule, the filter is flooded with a flavoring chemical, which in turn, flavors the inhaled smoke. They are different than traditional flavored products, where manufacturers add flavors (for example, menthol) to tobacco leaf at the processing stage. The range of flavors that are marketed globally has been rapidly increasing, and also includes non-flavored capsules, where water is added to provide a cooling sensation [9]. Some of the flavors introduced by capsules are variations of mint/menthol, fruits (e.g. blueberry, strawberry), beverages/cocktails (e.g. green tea, mojito, whisky), etc. [9].

**Fig 1:**
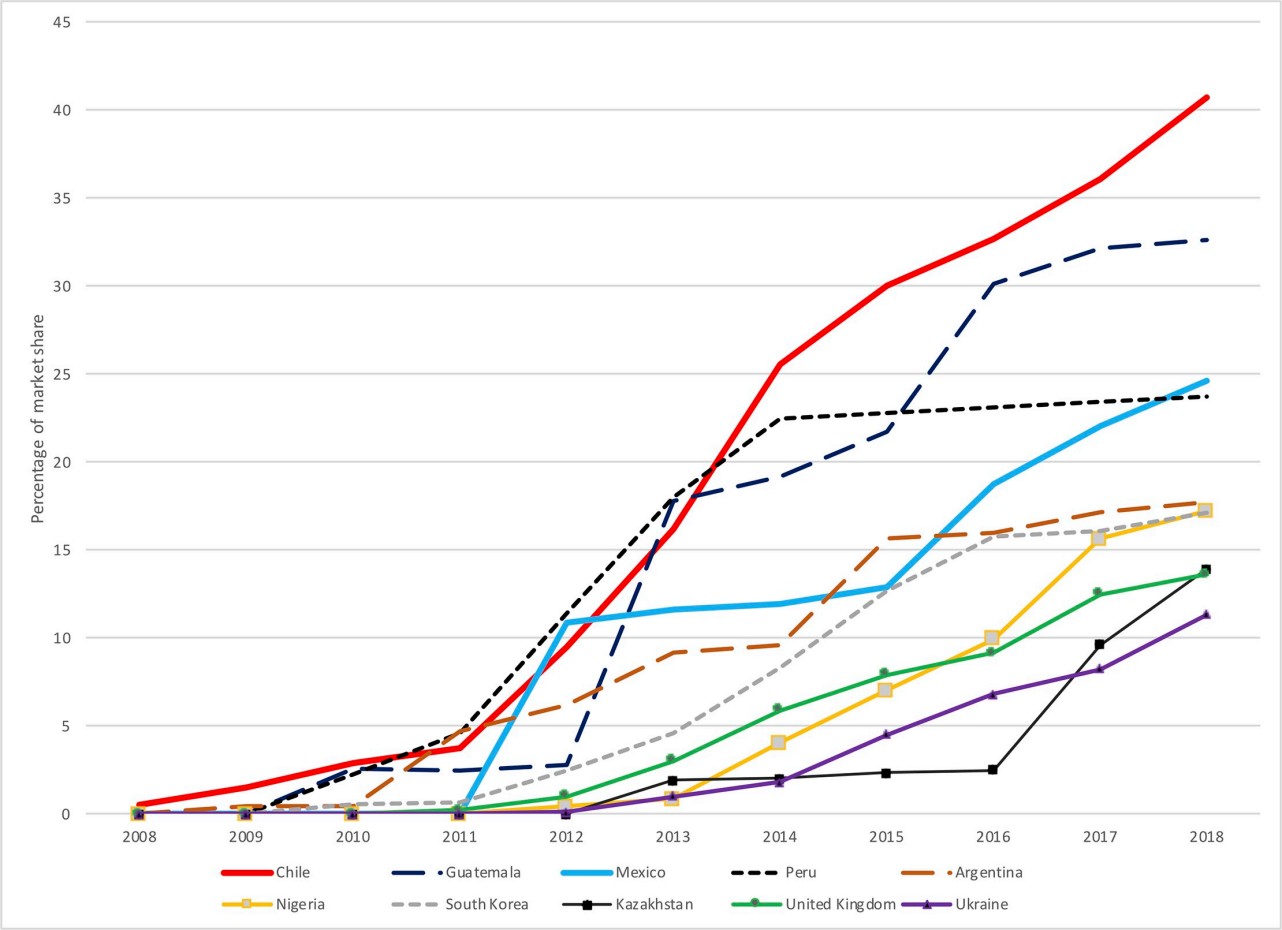
Top ten countries with the highest market shares for flavor capsule cigarettes, 2008–2018. Source: similar to Fig 1 from [9], with an extra year from [7].

Flavor capsule cigarettes have displayed the fastest growth amongst Latin American countries. The top five countries with the largest market share in the world for flavor capsule cigarettes are from this region: Chile, Guatemala, Mexico, Peru and Argentina (Fig 1). In each country, the rate of increase has been staggering: in Chile, the country with the world’s highest market penetration for these products, the average annual growth rate in the 2013–2018 period was 20% (a total increase of 151%) [7]. In the other four countries in the same time period, annual average growth ranged from 5.7% in Peru to 16.2% in Mexico (with total growth rates from 32% in the former to 112% in the latter) [7]. These rapid increases in the use of flavor capsule cigarettes have been observed closely by researchers and the tobacco control community, which have warned about the negative impact these products have on national efforts to curb smoking, especially among youth [8–11].

Flavor capsule cigarettes have been shown to be associated with more appeal and preferred taste for consumers, as they are perceived as smoother, providing fresher breath, and reducing cigarette odor [11]. In Mexico, which has the world’s third largest market share for these products, their use is associated with age (younger people use them more than middle-aged people, though adults in the 55–64 age group consume them as much as youths) and sex (females use them far more than males), similar to findings in the USA and Australia [12]. In the case of the United Kingdom, smoking cigarettes with capsules is also associated with age (as younger people are more likely to smoke them) but not with sex [13]. This evidence is also consistent with findings of other studies that present flavored cigarettes as gateways to other tobacco consumption, flavored or not [14, 15].

In addition to Chile having the world’s highest market share for flavor capsule cigarettes, as mentioned above, Chile also has among the world’s highest prevalence of tobacco use among youths, despite having adopted regulations to decrease its use. In 2006, after Chile’s ratification of the World Health Organization’s (WHO) Framework Convention on Tobacco Control (FCTC), Law 20105 increased the legal age to buy tobacco products to 18 (from 16), banned smoking in schools, forbade direct advertisement in any kind of mass media (though advertisement in point-of-sales is still allowed) and promotion in events (e.g. concerts, races, etc.), increased the size of health graphic warnings to 50% of both of the main faces of the packaging, and restricted smoking in public closed spaces (e.g. work places, bars, restaurants, etc.). At different points in time after 2005 (e.g. 2010, 2014), Chile also increased tobacco excise taxes, which along with the other measures had a direct effect on smoking among youths [16, 17].

As a result of these regulations, the smoking prevalence for the overall population (12–65 years old) fell from 42.4% in 2006 to 33.4% in 2016 [18, 19]. More impressively, the smoking prevalence among school children (typically, in the 12–17 age group) fell from 42% in 2005 to 19.2% in 2017 (16.6% for boys and 21.8% for girls)[20]. However, between 2011 and 2015 smoking prevalence plateaued, though it further decreased in 2017 [20]. This dynamic coincides with a rapid increase in the market share of flavor capsule cigarettes, which increased from only 3.7% of sales in 2011 to 30% in 2015 and to 40.7% in 2018 [7].

The objective of this article is to examine the variables associated with smoking flavor capsule cigarettes in Metropolitan Santiago de Chile (i.e. Greater Santiago de Chile) to understand better the increased use of these products and to consider the policies that should be implemented to deter such use.

## Material and methods

### Study sample

To generate an estimate, independent of the tobacco industry, of the effects of key socioeconomic variables on smoking behavior (e.g., choosing of flavored/unflavored, and/or illicit cigarettes) in Metropolitan Santiago de Chile (40% of the Chile’s total population), a poll of 851 smokers was implemented in mid-2017 (sampling error estimated at 3.3%). The sample methodology was designed by the Center for Territorial Intelligence (Centro de Inteligencia Territorial) of the Universidad Adolfo Ibáñez for the sample to be representative of Metropolitan Santiago de Chile’s smoking population, that is, those who permanently reside in the area but travel within Metropolitan Santiago de Chile for several reasons (e.g., work, education, leisure,). For this, a mix of random spatial sampling and a convenience quota sampling were utilized. A total of 424 points of high pedestrian traffic were identified from which 40 were randomly selected to be part of the survey, each following a quota (explained below).

To identify the high traffic points, data from two sources were used: a) the last Survey of Origin-Destination in Metropolitan Santiago de Chile (EOD in its Spanish acronym) [21]; and b) data from the Inland Revenue Service (SII in its Spanish acronym) of the built surfaces in square meters by type of use (for example, educational, commercial, etc.). The first source has data on visits made by people to 767 high traffic areas. The second source has data on the use of soil per square meter for 66,467 city blocks. The data from these two datasets were linked to estimate the following model (without intercept) by ordinary least squares regression (OLS):

$$N_{i}=\beta_{1}C_{i}+\beta_{2}S_{i}+\beta_{3}E_{i}+\beta_{4}O_{i}+\beta_{5}Q_{i}+\beta_{6}H_{i}+\beta_{7}T_{i}+\beta_{8}G_{i}$$

where *N*_*i*_ is the estimated number of visits to the high traffic area *i*, and the independent variables are the square meters of area *i*, for commerce (*C*_*i*_), sports facilities (*S*_*i*_), educational and cultural facilities (*E*_*i*_), office buildings (*O*_*i*_), churches and cultural places (*Q*_*i*_), healthcare facilities (*H*_*i*_), transportation and telecommunications venues (*T*_*i*_) and parks (*G*_*i*_).

Once the coefficients were estimated, they were used to estimate the number of visits to each of the 66,467 blocks included in the SII data. The data on estimated number of visits were used to identify the 400 blocks with an estimated number of visits higher than the 160 closest neighbors (to have well-scattered points). Because of the criteria of 160 neighbors and geometrical irregularity, 426 blocks were ultimately selected. Then, of these 426 blocks, 40 were randomly selected with probability proportional to the estimated number of visits (random spatial sample).

In each of the 40 blocks, a “high traffic point” was identified, which could be buildings such as offices or hospitals, or the intersection of two important avenues. The interviews were conducted at each of the 40 points of high pedestrian traffic and, in the case of difficulty finding smokers, the pollsters were permitted to move in a radius of approximately 500 meters. Smokers over the age of 13 were interviewed at each point, following the age and sex structure of the expanded sample of the survey on the use of drugs in the overall population that the Chilean government implemented in 2014 (convenience quota sampling)[22]. The results of a government survey, conducted in 2016 [19], came out after the fieldwork, and therefore the sample was re-weighted to have the same representativeness as the newer survey.

Using a tablet to store the information, smokers were interviewed utilizing a questionnaire that collected a number of measures discussed below. In addition, a photograph was taken of the front and side of the smoker's cigarette pack. Of the total, 41 interviews were discarded because the photos taken for those interviews were not sufficiently clear to gather the required information, leaving a final sample of 810 smokers. Flavor capsule cigarettes were identified using the photographic records of the cigarette packs. Flavor capsule cigarettes were defined as those whose packaging promotes them as having capsule flavors of any type. Descriptors such as “on button symbol”, “click”, “crush”, etc. were used to identify these products. When the picture was not clear on the product characteristics (very few cases), actual products’ packages were used to identify products.

The fieldwork was conducted by the Centre for Microdata (CM) of the Universidad de Chile. As such, the IRB clearance was obtained from the Ethics Committee of the Faculty of Economics and Business of the University of Chile that oversees CM surveys. Specifically, such a body authorized the collection of data on smoking behavior in Metropolitan Santiago de Chile. Informed participant consent was obtained verbally, after the enumerator read aloud an introduction explaining the objectives of the study, the funding bodies behind the study and the confidentiality of any individual-level data collected. No names or addresses of participants were requested.

### Measures

The questionnaire, which draws from similar previously-validated instruments utilized in other regional countries, including Colombia [23], was piloted (approximately 20 to 30 participants) among smoker students at the Universidad de Chile to determine if it was successfully capturing the sought-after phenomena.

As mentioned above, information was collected on the attributes that determined the last purchase of a pack of cigarettes (the one for which a picture was taken at the moment of the survey). The precise question, for which only one option was allowed, was “When you chose the package of cigarettes you purchased last, you did it based on: a) its taste/flavor; b) that it would be less harmful than the rest; c) its price” (in Spanish: “¿Cuando eligió la marca de cigarillos en su última compra, pensó principalmente en: a) sabor; b) en que causaría menos daño; c) en el precio”). From this question a set of three categorical variables using the three possible answers were constructed.

A separate question was asked to record the price of the last pack bought. Concretely, the question was: “How much did you pay for the last pack of cigarettes you have bought?” (in Spanish: “Ahora piense en el pago por cajetilla de cigarrillos, ¿Cuánto pagoó por la última cajetilla de cigarrillos que compró para usted?”). With that information and the size of the last pack purchased, a unitary price was estimated.

Other questions, regarding smoking behavior and/or characteristics of purchase, are standard to similar survey research about smoking. Among them: age of smoking initiation in years; frequency of smoking (in number of cigarettes smoked per day, week or month); place of purchase of last package of cigarettes (e.g. store, street vendor, supermarket, duty-free shop, abroad, etc.); etc.

Socioeconomic variables in the sample and used in the analyses include age in years, sex, educational achievement and occupational status. Education is recorded in terms of years of education. From this variable, a number of categories was constructed and used. In a country with a relatively high average of schooling (in years) it makes more sense to differentiate population by levels of education completed, which may provide a better signal of socioeconomic status than the continuous variable in years. This practice has been followed in other studies [17, 24]. Five categories are considered: 1) primary incomplete, which includes those with no education and some primary education; 2) secondary incomplete, including those with complete primary education, and some secondary education; 3) secondary complete; 4) some college/tertiary education; and 5) college/tertiary education completed.

Finally, occupational status is measured from the question: “In which activity you spent more time last week: a) working; b) searching for work; c) retired; d) studying; e) house work; f) incapacitated permanently to work; g) other” (in Spanish, “En qué actividad ocupó la mayor parte de su tiempo la semana pasada: a) trabajando; b) buscando trabajo; c) pensionado; d) estudiando; e) oficios del hogar; f) incapacitado permanentemente para trabajar; g) otros”). From this variable, a categorical variable was constructed with a value of 1 if working (outside home) and 0 otherwise.

### Statistical analysis

With the objective to better understand the statistical relationships between traits of smokers and the consumption of flavor capsule cigarettes, four discrete-choice (probit) models were estimated on the decision to smoke flavor capsule cigarettes. In the first two models, the decision to smoke flavor capsule cigarettes (equal to one if respondents smoke flavor capsule cigarettes) is explained by the following characteristics of the smoker: age, age squared (to test for non-linearities), sex (equal to 1 if the smoker is a woman), preference for the taste/flavor or price (the reference category is “cigarettes consumed are perceived to be less harmful than the rest”), level of education (as defined in the previous sub-section), number of cigarettes smoked per day, and employment status (as defined in the previous sub-section). Model 2 also included as an independent variable the natural logarithm of the reported price paid per cigarette. The reported price was included in order to determine a possible relationship between the price paid and the presence of flavor related to the purchased cigarettes.

Models 3 and 4 are similar to the first two but instead of including a continuous variable for age, it is divided into four groups: a) 25 years old and less; b) 26–34 years old; c) 35–49 years old; and d) 50 years and older. The partition of age was done to create groups of similar size except for the first group, which was constructed to include those who were students (94% of current students are in this first group). Generating discrete age groups permitted the inclusion of an interaction between sex and these age groups with the intention of gauging differential probabilities of use between males and females of different ages. Model 3 does not include cigarette prices, while Model 4 does include them.

A difference of means test between the reported price for flavor capsule cigarettes and that of regular cigarettes was also conducted to assess statistical differences between prices of these two sets of products.

## Results

**Fig 2:**
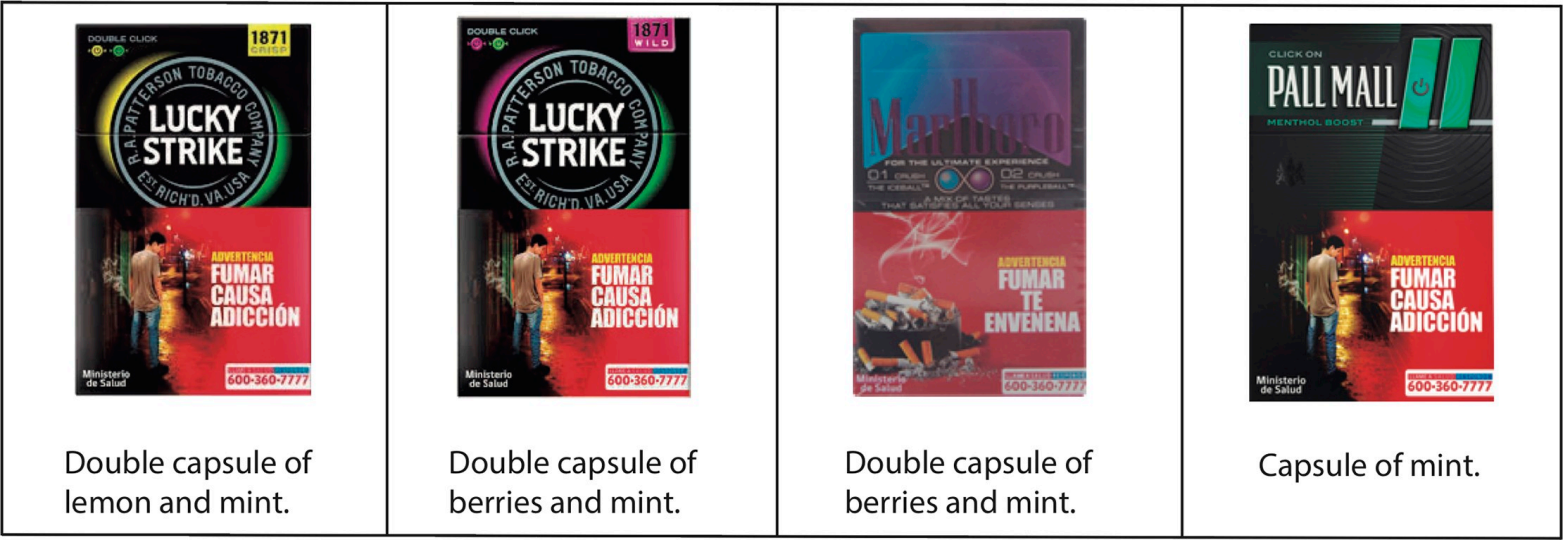
Examples of packages of flavor capsule cigarettes in Chile. Source: photographs from smokers’ survey.

Fig 2 shows examples of typical flavor capsule cigarettes found in the study. The photographic evidence reveals that, despite the health graphic warning covering half the package, attractive colors associated with and promoting flavors (e.g. green or blue for mint, yellow for lemon, etc.), are used in all cases.

Table 1 shows descriptive statistics for weighted and unweighted samples, classifying smokers according to the product they have chosen in their last purchase. Flavored cigarettes are pooled, as only 6 observations (of the 810) are non-capsule flavored cigarettes. As can be seen, weighted and unweighted estimates are similar (which is unsurprising because the unweighted population was defined following survey results only two years older than the weighted one). A majority of smokers chose cigarettes based on taste/flavor, followed by those who chose it based on price. The group who smoked capsule flavored cigarettes have, on average, a higher educational level.

**Table 1:** Descriptive statistics, by type of cigarette of last purchase.

	Weighted			Unweighted		
Sociodemographics variables	Non flavored	Flavor (Any type)	Total	Non flavored	Flavor (Any type)	Total
Percentage of non- flavor cigarettes	100.0%	-	60.1%	100.0%	-	60.0%
	-	-	(0.49)	-	-	(0.49)
Percentage of flavored non-capsule cigarettes	-	1.6%	0.6%	-	1.9%	0.7%
	-	(0.13)	(0.08)	-	(0.14)	(0.09)
Percentage of flavored capsule cigarettes	-	98.4%	39.2%	-	98.1%	39.3%
	-	(0.13)	(0.49)	-	(0.14)	(0.49)
Women	43.9%	59.3%	50.0%	42.6%	60.5%	49.8%
	(0.50)	(0.49)	(0.50)	(0.49)	(0.49)	(0.50)
Age (mean)	42.3	34.7	39.3	43.1	33.7	39.3
	(13.0)	(12.4)	(13.3)	(15.1)	(14.2)	(15.4)
Population 25 years old or younger	11.4%	26.4%	17.4%	14.8%	34.0%	22.5%
	(0.32)	(0.44)	(0.38)	(0.36)	(0.47)	(0.42)
Population between 26 and 34 years old	16.6%	29.8%	21.9%	14.0%	25.3%	18.5%
	(0.37)	(0.46)	(0.41)	(0.35)	(0.44)	(0.39)
Population between 35 and 49 years old	41.4%	29.3%	36.6%	35.8%	24.7%	31.4%
	(0.49)	(0.46)	(0.48)	(0.48)	(0.43)	(0.46)
Population of 50 years old or older	30.6%	14.5%	24.2%	35.4%	16.0%	27.7%
	(0.46)	(0.35)	(0.43)	(0.48)	(0.37)	(0.45)
Smokers who chose flavor/taste in last purchase	59.3%	75.9%	65.9%	59.3%	75.0%	65.6%
	(0.49)	(0.43)	(0.47)	(0.49)	(0.43)	(0.48)
Smokers who chose "less harmful" cigarettes in last purchase	7.9%	6.8%	7.5%	8.6%	6.5%	7.8%
	(0.27)	(0.25)	(0.26)	(0.28)	(0.25)	(0.27)
Smokers who chose cigarettes because of price in last purchase	32.8%	17.3%	26.6%	32.1%	18.5%	26.7%
	(0.47)	(0.38)	(0.44)	(0.47)	(0.39)	(0.44)
Population with incomplete primary education	3.5%	1.0%	2.5%	4.5%	1.2%	3.2%
	(0.18)	(0.10)	(0.16)	(0.21)	(0.11)	(0.18)
Population with incomplete secondary education	14.2%	8.2%	11.8%	18.1%	14.5%	16.7%
	(0.35)	(0.27)	(0.32)	(0.39)	(0.35)	(0.37)
Population with complete secondary education	35.2%	35.9%	35.5%	34.0%	35.5%	34.6%
	(0.48)	(0.48)	(0.48)	(0.47)	(0.48)	(0.48)
Population with incomplete superior education	13.2%	20.2%	16.0%	11.9%	18.5%	14.6%
	(0.34)	(0.40)	(0.37)	(0.32)	(0.39)	(0.35)
Population with complete superior education	34.0%	34.7%	34.3%	31.5%	30.2%	31.0%
	(0.47)	(0.48)	(0.47)	(0.47)	(0.46)	(0.46)
Working (outside home) population	79.2%	72.2%	76.4%	75.1%	64.2%	70.7%
	(0.41)	(0.45)	(0.42)	(0.43)	(0.48)	(0.46)

Strandard deviation in parentheses

Flavor (Any type) includes capsule and non-capsule cigarettes

Table 2 shows the percentage of smokers who consume flavor capsule cigarettes. Of the total sample, 39.2% consumed flavor capsule cigarettes, with the percentage higher among women (46.7% versus 32.3% for males) and those 25 years old or younger (60%). Women in the first age group showed the highest prevalence of smoking of flavor capsule cigarettes (71.9%).

**Table 2:** Percentage of flavor capsule cigarette smokers by age and sex.

**Total**					
** **	**25 years old or younger**	**26–34 years old**	**35–49 years old**	**50 years old or older**	**Total**
Proportion of group smoking flavor capsule cigarettes	60.0%	54.4%	31.7%	23.0%	39.5%
Standard deviation	(0.49)	(0.50)	(0.47)	(0.43)	(0.49)
**Men**					
** **	**25 years old or younger**	**26–34 years old**	**35–49 years old**	**50 years old or older**	**Total**
Proportion of group smoking flavor capsule cigarettes	49.8%	49.3%	25.1%	14.4%	32.3%
Standard deviation	(0.50)	(0.50)	(0.44)	(0.36)	(0.47)
**Female**					
** **	**25 years old or younger**	**26–34 years old**	**35–49 years old**	**50 years old or older**	**Total**
Proportion of group smoking flavor capsule cigarettes	71.9%	59.5%	37.7%	32.1%	46.7%
Standard deviation	(0.45)	(0.49)	(0.49)	(0.47)	(0.50)

Strandard deviation in parentheses

Incorporating the average intensity of smoking in the sample, it was estimated that the market share of flavor capsule cigarettes in Metropolitan Santiago de Chile was 32.3% (CI 95%: 28.3%-36.2%), lower than the 39.6% reported by Euromonitor for 2017 at the national level [25].

Table 3 shows the results from the difference of means test between prices of flavor capsule vs non-flavored cigarettes. The former was significantly higher than the latter, with an average unit value 14% higher than the non-flavored unit value. At the time of the survey collection the average exchange rate was USD 1 = CLP 668.

**Table 3:** Unit value per stick by cigarette type.

Cigarette Type	Not Flavored	Flavor capsule	Difference	P-value
Mean	$141.6	$161.2	-$19.6	0.000
Standard Error	(1.89)	(2.11)	(2.89)	-

**Fig 3:**
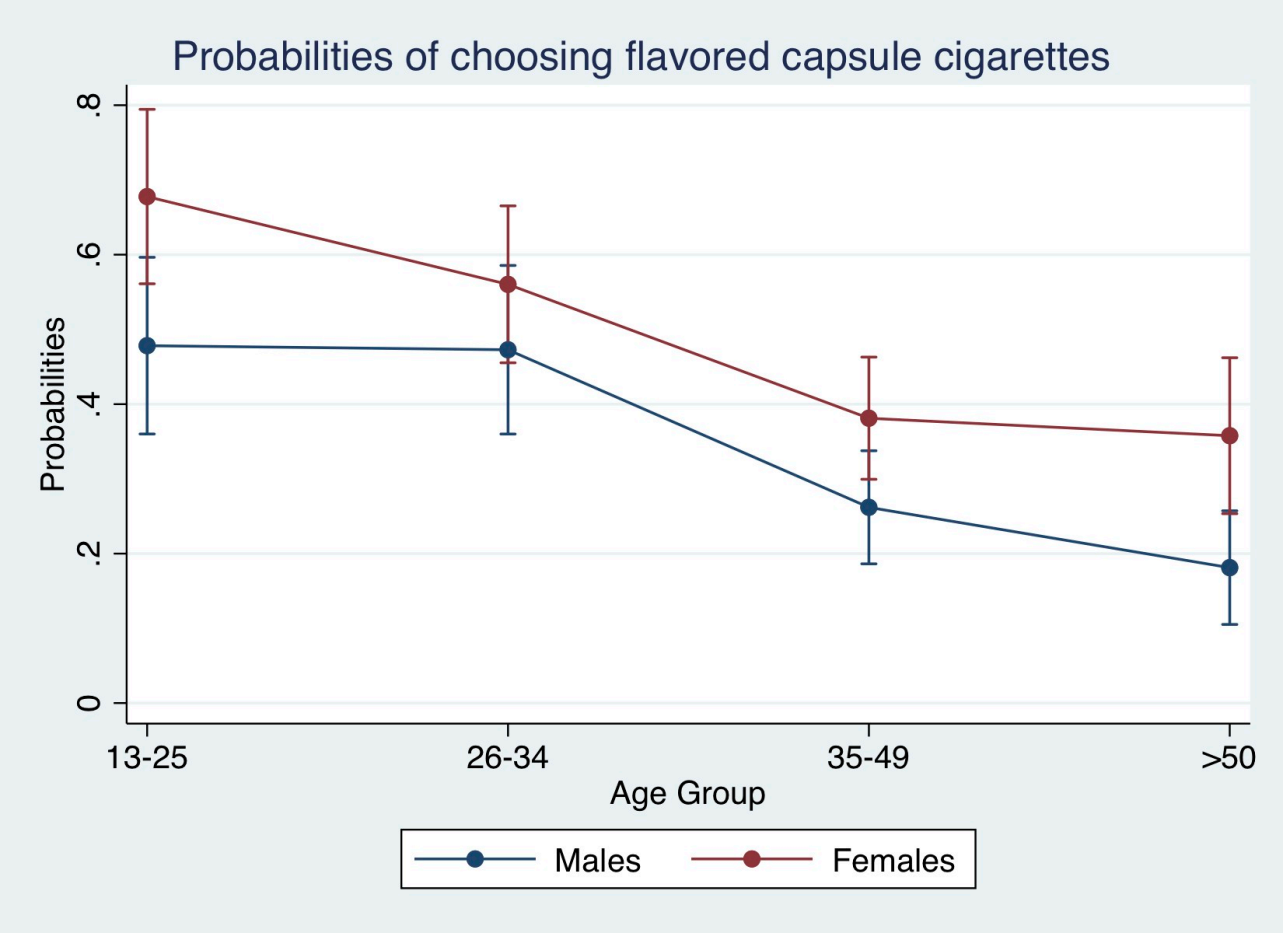
Confidence interval of unit value by type of cigarette. Source: Own from data from the present survey.

In Fig 3 the confidence interval (95%) of the unit value for flavored and not flavored cigarettes are presented: 95% of unit values of not-flavored cigarettes range between $137 and $147 Chilean pesos, while 95% of unit values of flavored cigarettes range between $154 and $165 Chilean pesos. There is no significant overlapping between prices of both types of cigarettes and, while there are cheaper and premiums brands, the price dispersion between them is not large, which is a reasonable expectation in a largely concentrated market (British American Tobacco controls more than 95% of the legal market [7]).

The results of the probit models are presented in Table 4. The negative and statistically significant coefficient of age in Models 1 and 2 demonstrated an inverse and meaningful relationship with the likelihood of smoking flavor capsule cigarettes. That is, each year less in the smoker’s age increased the likelihood of preferring flavor capsule cigarettes by, on average, between 0.8 and 0.9 percentage points. In turn, the positive and statistically significant coefficient for the age squared variable demonstrates that the probability of smoking flavor capsule cigarettes decreases (is less negative) the older the smoker is.

**Table 4:** Marginal effects of probit estimate on the decision to smoke flavor capsule cigarettes.

	(1)	(2)	(3)	(4)
VARIABLES	Model 1	Model 2	Model 3	Model 4
Age	-0.010***	-0.009***		
	(0.001)	(0.001)		
Age squared	0.000***	0.000**		
	(0.000)	(0.000)		
Sex (ref: Male)	0.139***	0.140***	0.135***	0.137***
	(0.034)	(0.034)	(0.035)	(0.034)
Preference taste/flavor (ref: less harmful)	0.051	0.058	0.047	0.055
	(0.072)	(0.071)	(0.072)	(0.070)
Preference price (ref: less harmful)	-0.129*	-0.089	-0.121	-0.076
	(0.076)	(0.077)	(0.075)	(0.077)
Ln price per stick		0.140**		0.153**
		(0.064)		(0.065)
Primary incomplete (ref: College/tertiary complete)	-0.116	-0.090	-0.146	-0.134
	(0.115)	(0.115)	(0.111)	(0.109)
Secondary incomplete (ref: College/tertiary complete)	-0.107*	-0.087	-0.104*	-0.100*
	(0.056)	(0.057)	(0.058)	(0.055)
Secondary complete (ref: College/tertiary complete)	0.010	0.017	0.006	-0.003
	(0.040)	(0.040)	(0.042)	(0.037)
College/tertiary incomplete (ref: College/tertiary complete)	0.049	0.049	0.048	0.047
	(0.054)	(0.053)	(0.054)	(0.054)
Number of cigarettes smoked per day	-0.006*	-0.005	-0.006**	-0.005
	(0.003)	(0.003)	(0.003)	(0.003)
Employed (ref: inactive/unemployed)	-0.042	-0.048	-0.035	-0.047
	(0.041)	(0.041)	(0.042)	(0.042)
26–34 years old (ref: 25 and less)			-0.052	-0.061
			(0.061)	(0.060)
35–49 years old (ref: 25 and less)			-0.250***	-0.256***
			(0.056)	(0.054)
50 years old and more (ref: 25 and less)			-0.314***	-0.307***
			(0.055)	(0.055)
Observations	810	810	810	810

Standard errors in parentheses

*** p<0.01

** p<0.05

* p<0.1

If the smoker was a woman, the likelihood of preferring flavor capsule cigarettes increased between 13.4 and 13.5 percentage points (Models 1 and 2). In addition, Model 2 revealed a positive relationship between the price paid and the consumption of flavor capsule cigarettes, indicating that these cigarettes tended to be more expensive, consistent with the result of the difference of means test. There was neither statistical relationship between participation in the labor market and smoking flavor capsule cigarettes, nor on different educational level (except secondary incomplete education in Model 4).

For Models 3 and 4, the main results are consistent, as women were more likely to choose flavor capsule cigarettes than males; and younger age groups are also more likely to choose them than older age groups. When interacting sex and age groups (for instance, in Model 4), the probability of choosing flavor capsule cigarettes is statistically higher for both females and males in the “25 years and less” group than those 35 years and older. Concretely, individuals that are in the 35–49 age group are 25%-26% less likely to choose capsule flavors cigarettes than those in the “25 and less” group.

**Fig 4:**
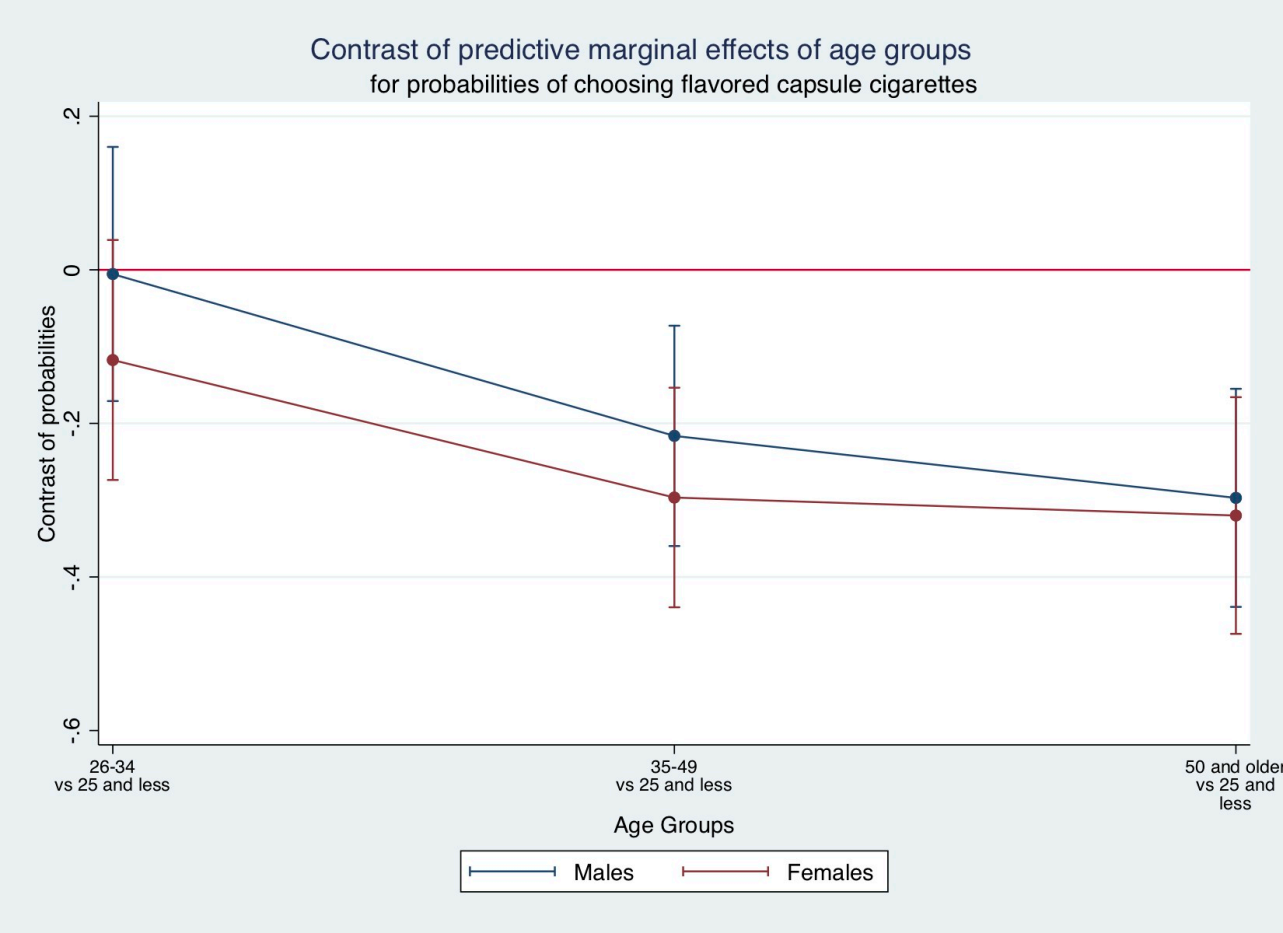
Predicted probabilities of choosing flavor capsule cigarettes. Source: authors’ calculations based on results from Model 4.

Fig 4 shows that within each age group there are no statistically significant differences among females and males, though they exist across age groups (based on marginal effects found for Model 4).

## Discussion

The results presented here indicate that the transition of the cigarette sales market toward flavor capsule cigarettes is particularly aimed at attracting new consumer groups, which is consistent with findings from other countries [13]. For example, for teenagers, the average age of initiation was 13.8 years old in 2017, a figure that has barely changed since 2011, when it was 13.4 [20]. Relatively new flavor capsule cigarettes are more appealing to teenagers. It cannot be inferred from these data that the consumers targeted by the firms selling the flavored cigarettes are of any particular income group, but it is suggestive that these products have a significantly higher price than conventional non-flavored cigarettes. A closer look at prices shows that the range of unit prices of flavored capsule cigarettes is almost the same as the one for non-flavored cigarettes, but the fact that the average unit value is higher suggests that consumers purchasing them may have higher incomes.

The challenge of flavor capsule cigarettes is growing in many countries. Projections from Euromonitor suggest that by 2023, at least 11 countries will have a flavor capsule cigarette market share of more than 15% (Chile being the highest, with 50% of the market). This transition presents a grave challenge for public health. The FCTC guidelines to the implementation of Articles 9 and 10 recommends countries to “regulate, by prohibiting or restricting, ingredients that may be used to increase palatability in tobacco products” and that they “should prohibit or restrict ingredients that have colouring properties in tobacco products” [26].

Observing these trends, governments are taking concrete steps to adjust policy to address the challenge. In 2012, Brazil took one of the most comprehensive approaches by completely banning tobacco additives, including all flavors, though due to legal challenges the regulation has yet to be implemented [27–29]. Canada has banned all flavors, including capsules [30], and countries of the European Union are scheduled to phase out any flavorings that mask the taste and smell of tobacco by 2020 [6]. Other countries that have banned at least some flavors that mask the taste and smell of tobacco are Ethiopia [1], Moldova, Turkey (only menthol)[31], and Senegal [32]. Noting the threat to their profits, the tobacco industry and its proxies have aggressively fought these bans [33].

In the case of Chile, the Senate has passed a bill to ban additives, including menthol, but the legislation has stalled in the lower Chamber of Parliament, mostly due to pressure by the tobacco industry [34–36]. In addition, it is not clear that such a ban includes flavor capsule cigarettes, as it would only forbid products with additives added to the tobacco at the “time of manufacturing the products” [37].

Chile has made significant progress regulating tobacco products beginning in 2006, including increasing excise taxes and establishing smoke-free zones, among other measures. However, the tobacco industry has promoted the consumption of flavor capsule cigarettes to counteract the decline in consumption, increasing flavor capsule cigarettes’ market share from 7% in 2011 to 40.7% in 2018 [25]. This growth in the proportion of sales is no coincidence, and is a likely explanation for the fact that the prevalence of smoking in school-aged children had a relatively small decrease since 2011, and is the highest in the region, especially for girls [38].

In countries with growing prevalence, the tactic is helping to grow the market by attracting new consumers [9]. Thus, to reduce cigarette consumption (especially among youth), restricting or even forbidding cigarette flavorings of all types—including or perhaps even especially flavor capsules—would be an effective strategy [26]. In this context, the government of Chile still has a serious pending task to reduce the future costs of this epidemic. On a more optimistic note, banning flavors is a relatively straightforward solution to a very grave problem.

The analysis has a number of limitations. First, the survey did not collect information on smokers who buy individual sticks (illegal in Chile). There are no studies estimating the prevalence of this practice, though health authorities, informally, do not consider it a widespread practice. In the course of the survey collection, the number of cases that reported buying individual sticks was not large. Unfortunately, those cases were not recorded, as one of the conditions to conduct the interview was having a package of cigarettes to show. Second, the contraband of cigarettes is a topic that is frequently put on the agenda by the tobacco industry to demand tobacco tax reductions [39, 40]. It is not a topic that is directly related to flavored capsule cigarettes and has been addressed extensively elsewhere [24, 41, 42]. Also, the sample collected for this study shows that the prevalence of illicit flavored capsule cigarettes is lower than the prevalence of illicit non-flavored cigarettes (5.7% versus 13.3%). Third, the collection instrument does not have any socioeconomic status (SES) variable, except for educational achievement, which restricts the possibility of inquiring about the association between SES and choosing flavored capsule cigarettes. The fact that the collection instrument was a short survey, administered on the street, was a factor behind the decision on not to include other SES variables. Underreporting of income, a common problem in surveys, was another reason we excluded it [43]. We do not consider this limitation to produce any particular bias in the estimates provided in the previous section. Finally, sample size is a limitation to develop analysis including interactions among many variables. Though the sample size is deemed correct for the type of analysis conducted, it is not representative by commune of residence (another SES variable) and does not allow to interact variables, such as gender, age or educational status, with unit value ranges and/or smokers’ preferences for chosen cigarettes. Such interactions could possibly have given more information on the motivations of consumers to choose between products. An example [11] shows that US smokers that preferred flavor capsule cigarettes, for instance, “were more likely to view their brand as more stylish, smoother and less harmful than people who smoked regular premium brands.”

## Supporting information

S1 File This is the database with information for the 810 individuals sampled and with complete information. Photographs of cigarette packs are available from corresponding author.
(DTA)

S2 File Complete report (in Spanish) with results of the study, location of data collection points, questionnaire, etc. (PDF)

## References

[1] World Health Organization. Case studies for regulatory approaches to tobacco products: menthol in tobacco products Geneva: World Health Organization; 2018. Report No.: WHO/NMH/PND/18.1.

[2] Anderson SJ. Marketing of menthol cigarettes and consumer perceptions: a review of tobacco industry documents. Tob Control. 2011;20 Suppl 2,:ii20–8. 21504928 10.1136/tc.2010.041939 PMC3088454

[3] Huang L-L, Baker HM, Meernik C, Ranney LM, Richardson A, Goldstein AO. Impact of non-menthol flavours in tobacco products on perceptions and use among youth, young adults and adults: a systematic review. Tobacco Control. 2017;26(6):709 10.1136/tobaccocontrol-2016-053196 27872344 PMC5661267

[4] Kabbani N. Not so Cool? Menthol’s discovered actions on the nicotinic receptor and its implications for nicotine addiction. Frontiers in Pharmacology. 2013;4:95 10.3389/fphar.2013.00095 23898298 PMC3720998

[5] Wertz MS, Kyriss T, Paranjape S, Glantz SA. The Toxic Effects of Cigarette Additives. Philip Morris' Project Mix Reconsidered: An Analysis of Documents Released through Litigation. PLoS medicine. 2011;8(12):e1001145 10.1371/journal.pmed.1001145 22205885 PMC3243707

[6] European Parliament. Directive 2014/40/EU. In: European Parliament, editor. Strasbourg,2014.

[7] Euromonitor International. Passport data 2019 [Available from: https://www.portal.euromonitor.com/portal/magazine/homemain. Access date: 6th March 2019.

[8] Thrasher JF, Islam F, Barnoya J, Mejia R, Valenzuela MT, Chaloupka FJ. Market share for flavour capsule cigarettes is quickly growing, especially in Latin America. Tobacco Control. 2017;26(4):468 10.1136/tobaccocontrol-2016-053030 27329114 PMC5177543

[9] Moodie C, Thrasher JF, Cho YJ, Barnoya J, Chaloupka FJ. Flavour capsule cigarettes continue to experience strong global growth. Tobacco Control. 2018:tobaccocontrol-2018-054711. 10.1136/tobaccocontrol-2018-054711 30368482

[10] Scollo M, Bayly M, White S, Lindorff K, Wakefield M. Tobacco product developments in the Australian market in the 4 years following plain packaging. Tobacco Control. 2018;27(5):580 10.1136/tobaccocontrol-2017-053912 28993520

[11] Thrasher JF, Abad-Vivero EN, Moodie C, Connor RJ, Hammond D, Cummings KM, et al Cigarette brands with flavour capsules in the filter: trends in use and brand perceptions among smokers in the USA, Mexico and Australia, 2012–2014. Tobacco Control. 2016;25(3):275 10.1136/tobaccocontrol-2014-052064 25918129 PMC4798911

[12] Thrasher JF, Abad-Vivero EN, Moodie C, O'Connor RJ, Hammond D, Cummings KM, et al Cigarette brands with flavour capsules in the filter: trends in use and brand perceptions among smokers in the USA, Mexico and Australia, 2012–2014. Tob Control. 2016;25(3):275–83. 10.1136/tobaccocontrol-2014-052064 25918129 PMC4798911

[13] Moodie C, MacKintosh AM, Thrasher JF, McNeill A, Hitchman S. Use of Cigarettes With Flavor-Changing Capsules Among Smokers in the United Kingdom: An Online Survey. Nicotine & Tobacco Research. 2018. 10.1093/ntr/nty173 PMC6821177 30165686

[14] Klausner K. Menthol cigarettes and smoking initiation: a tobacco industry perspective. Tob Control. 2011;20 Suppl 2,:ii12–9. 21504927 10.1136/tc.2010.041954 PMC3088463

[15] Anderson SJ. Menthol cigarettes and smoking cessation behaviour: a review of tobacco industry documents. Tob Control. 2011;20 Suppl 2,:ii49–56. 21504932 10.1136/tc.2010.041947 PMC3088444

[16] Feigl A, Salomon J, Danaei G, Ding E, Calvo E. Teenage smoking behaviour following a high-school smoking ban in Chile: interrupted time-series analysis. Bulletin of the World Health Organization. 2015;93(7):468–75. 10.2471/BLT.14.146092 26170504 PMC4490811

[17] Guindon GE, Paraje GR, Chaloupka FJ. Association of Tobacco Control Policies With Youth Smoking Onset in Chile. JAMA Pediatrics. 2019:E1–E9. 10.1001/jamapediatrics.2019.1500 PMC6563596 31180455

[18] Consejo Nacional para el Control de Estupefacientes. Séptimo Estudio Nacional de Drogas en Población General de Chile. Santiago de Chile, Chile,2007.

[19] Observatorio Chileno de Drogas. Décimo Segundo Estudio Nacional de Drogas en Población General. Santiago de Chile, Chile,2017.

[20] Observatorio Chileno de Drogas. Décimo Segundo Estudio Nacional de Drogas en Población Escolar. Santiago de Chile, Chile,2018.

[21] Secretaría de Planificación de Transporte. Encuesta de Origen y Destino 2012. Chile: Ministerio de Teletransportes y Telecomunicaciones; 2015.

[22] Observatorio Chileno de Drogas. Décimo Primer Estudio Nacional de Drogas en Población General. Santiago de Chile, Chile,2015.

[23] Maldonado N, Llorente BA, Iglesias RM, Escobar D. Measuring illicit cigarette trade in Colombia. Tobacco Control. 2018. 10.1136/tobaccocontrol-2017-053980 29540558

[24] Paraje G, Araya D, Drope J. Illicit Cigarette Trade in the Metropolitan Santiago de Chile. Tobacco Control. 2018. 10.1136/tobaccocontrol-2018-054546 30554162

[25] International Euromonitor. Cigarettes in Chile. Euromonitor International; 2017.

[26] World Health Organization. Partial guidelines for implementation of Articles 9 and 10 of the WHO Framework Convention on Tobacco Control; Regulation of the contents of tobacco products and regulation of tobacco product disclosures. Adopted 2010, amended 2012.

[27] WHO Framework Convention on Tobacco Control. Brazil—Flavoured cigarettes banned 2012 [Available from: https://www.who.int/fctc/implementation/news/news_brazil/en/.

[28] Lencucha R, Drope J, Labonte R. Rhetoric and the law, or the law of rhetoric: How countries oppose novel tobacco control measures at the World Trade Organization. Soc Sci Med. 2016;164:100–7. 10.1016/j.socscimed.2016.07.026 27475056 PMC4994523

[29] Cancian N, Casado L. Brazil Supreme Court Upholds Anvisa Norm That Prohibits Flavored Cigarettes. Folha de Sao Paulo 2nd 2 2018.

[30] Minister of Justice of Canada. Tobacco and Vaping Products Act. In: Minister of Justice of Canada, editor. Ottawa,2018.

[31] Tobacco Control Legal Consortium. How Other Countries Regulate Flavored Tobacco Products [Available from: http://publichealthlawcenter.org/sites/default/files/resources/tclc-fs-global-flavored-regs-2015.pdf.

[32] Campaign for Tobacco Free Kids. Senegal [Available from: https://www.tobaccocontrollaws.org/legislation/country/senegal/summary.

[33] Gray A, Edgecliffe-Johnson A. Big Tobacco prepares to fight proposed ban on menthol cigarettes. Financial Times. 17th 11 2018.

[34] Roman J. La nueva batalla sobre el tabaco se traslada a la Unicef. Pauta. 26th 6 2018.

[35] Said J. Cultivo de tabaco, la nueva piedra de tope. El Líbero. 2019 16th 5 2019.

[36] Mostrador El. Girardi responsabiliza al Gobierno por lobby de tabacaleras que tiene estancada nueva Ley del Tabaco. El Mostrador. 19th 6 2017.

[37] Cámara de Diputados de Chile. Sesión 49º. In: Chile CdDd, editor. Valparaíso: Cámara de Diputados de Chile; 2015.

[38] Observatorio Chileno de Drogas. Décimo Primer Estudio Nacional de Drogas en Población Escolar. Santiago de Chile, Chile,2016.

[39] Observatorio de Comercio Ilícito, Cámara Nacional de Comercio. Informes por industria 2018 [Available from: http://www.observatoriocomercioilicito.cl/estudios/informes-por-industria/#1484577459575-1ef5d87e-6715.

[40] Tercera La. Comercio ilegal de cigarrillos en Chile creció un 386% en cinco años. La Tercera 1st Februery 2017.

[41] Paraje G. Illicit Cigarette Trade in Five South American Countries: A Gap Analysis for Argentina, Brazil, Chile, Colombia and Peru. Nicotine & Tobacco Research 2018:nty098-nty. 10.1093/ntr/nty098 29767772

[42] Paraje G. Chile: Tackling the Illicit Tobacco Trade In: Dutra S, editor. Confronting Illicit Tobacco Trade A Global Review of Country Experiences. Washington DC: The World Bank; 2019.

[43] Paraje G, Weeks M. Income nonresponse and inequeality measurement. Revista de Análisis Económico. 2010;25(December 2010):193–221.

