# Supplementary material for: The association between flavor capsule cigarette use and sociodemographic variables: Evidence from Chile
Source: PLoS One. 2019 Oct 23;14(10):e0224217. doi: 10.1371/journal.pone.0224217 (PMC6808557; doi:10.1371/journal.pone.0224217)
Supplement: S2 File — (PDF) [file pone.0224217.s002.pdf]

INFORME FINAL

ESTUDIO SOBRE HÁBITOS DE CONSUMO DE CIGARRILLOS EN EL GRAN SANTIAGO

NOVIEMBRE 2017

GUILLERMO PARAJE, PHD

DANIEL ARAYA, MA

ESCUELA DE NEGOCIOS

UNIVERSIDAD ADOLFO IBÁÑEZ

CHILE

UNIVERSIDAD ADOLFO IBÁÑEZ

“Este trabajo fue financiado por la American Cancer Society y por programa de Liderazgo en el Control del Tabaco de la Bloomberg School of Public Health de la Johns Hopkins University, a quienes los autores agradecen. Las entidades anteriores no tuvieron ningún rol en el diseño ni análisis de la encuesta, ni en la interpretación y/o escritura de este reporte, que es de exclusiva responsabilidad de los autores. Los autores declaran no tener ningún conflicto de interés percibido o real, relacionado con este trabajo”.

Resumen Ejecutivo

El presente informe tiene como objetivo presentar los resultados del “Estudio Sobre Hábitos de Consumo de Cigarrillos en el Gran Santiago”, cuya finalidad es conocer los hábitos y patrones de consumo de los fumadores que residen en el Gran Santiago, así como indagar en torno al contrabando de cigarrillos y su magnitud en esta zona del país.

La información fue recolectada mediante una encuesta presencial en distintos puntos del Gran Santiago, la que fue diseñada y ejecutada por el Centro de Microdatos de la Universidad de Chile. La encuesta fue dirigida específicamente a fumadores, donde, además de recolectar información respecto a los hábitos tabáquicos, se fotografiaron las cajetillas de cigarrillos de los encuestados con el fin de identificar su procedencia (legal o de contrabando) y otras características.

La muestra final se compuso de 810 fumadores, de edades entre 13 y 88 años. Ajustando la base por factor de expansión, esta se compone en un 50,02% de mujeres, 3,2% de menores de edad, 48,96% de personas entre 18 y 40 años, 42,53% de personas entre 41 y 60 años y un 5,29% mayores a 60 años. La mayor parte de los encuestados (76,4%) se encontraba trabajando; por el lado del nivel educativo, un 42% terminó la educación media y un 50,27% tiene algún estudio universitario o superior. Sobre la comuna de residencia de los encuestados, ellos se distribuyen con diferentes niveles de concentración en 42 de las 52 comunas pertenecientes a la Región Metropolitana, donde las que reportaron una mayor concentración fueron Puente Alto (10,70%), Santiago (8,48%), La Florida (5,82%), Maipú (4,62%) y Pudahuel (4,58%).

Los resultados muestran que un 10,9% de los fumadores se encontraba consumiendo cigarrillos de procedencia ilegal al momento de la encuesta, porcentaje mayor en hombres. Desagregando por grupos de edad, el consumo de marcas de contrabando resultó mayor en los fumadores de edades más avanzadas (mayores a 60 años), seguidos por los de menor edad (entre 13 y 17 años).

Adicionalmente, quienes consumían marcas de contrabando reportaron consumir una mayor cantidad de cigarrillos diarios que quienes se encontraban consumiendo cigarrillos legales (13,04 versus 8,74 en promedio).

También se encontraron importantes diferencias entre los precios de cigarrillos legales y de contrabando; el precio promedio que pagaron los fumadores por marcas de procedencia ilegal fue de $61,19 pesos por cigarrillo, menos de la mitad de los $160,11 pesos que pagaron los fumadores de cigarrillos legales. Adicionalmente, las marcas de contrabando reportaron un rango que va desde los $49,5 pesos por cigarrillo hasta los $160, mientras que los cigarrillos legales presentan un rango entre los $95 pesos hasta los $400.

Para un análisis más técnico, se realizaron dos estimaciones logísticas: sobre la probabilidad de elegir cigarrillos de contrabando y otra sobre la probabilidad de elegir variedades saborizadas. En la primera, se encontró que la edad de los fumadores, la preferencia por el precio, y la cantidad de cigarrillos consumidos diariamente tienen una relación positiva con la decisión de consumir marcas de contrabando, mientras que la situación laboral (en el caso de poseer empleo) y el máximo nivel educativo alcanzado reportaron relaciones negativas.

Sobre la regresión logística sobre la probabilidad de elegir variedades saborizadas, se encontró que la edad del fumador, el tiempo como fumador, la preferencia por el precio y la cantidad de cigarrillos diarios influyen negativamente en la decisión de elegir cigarrillos saborizados, mientras que las fumadoras mujeres presentaron una mayor probabilidad de elegir estas variedades.

Entre las conclusiones respecto al comercio ilegal de cigarrillos, destaca que **se estima una prevalencia del 10,9% de contrabando en el Gran Santiago** (cifra que contrasta con el 24% estimado por la industria[1]). Entre los fumadores con mayor probabilidad de elegir marcas de contrabando resaltan los hombres, los consumidores de mayor edad (con evidencia de que podrían también ser los de

[1] http://www.latercera.com/noticia/cnc-calcula-22-comercio-ilicito-cigarrillos-pais/

menor edad, es decir, los que suelen contar con menos ingresos) y los que consumen mas cigarrillos diariamente.

Sobre las variedades saborizadas, resalta que los fumadores más jóvenes (y los que se han iniciado hace poco en el tabaquismo) presentan una mayor probabilidad de preferir estas variedades; en ambos casos esta probabilidad disminuye al aumentar los años (de edad y como fumadores). Adicionalmente, las mujeres se presentan más susceptibles a preferir cigarrillos saborizados, mientras que quienes tienen preferencias por el precio tienen una menor probabilidad de preferir estas variedades.

Contenido

1. Presentación 7
2. Acta de Aprobación de Comité de Ética 8
3. Diseño Muestral 12
3.1. Metodología de muestreo para implementación de la encuesta en población flotante 12
3.1.1. Estimación de la atracción de viajes. 13
3.1.2. Definición y cuantificación de áreas de flujo. 15
3.1.3. Selección aleatoria de áreas de flujo. 15
3.1.4. Representación cartográfica de los puntos de mayor atracción en las áreas seleccionadas. 18
3.1.5. Determinación de las características de las personas a encuestar 21
4. Diseño y Programación del Cuestionario 24
5. Reclutamiento y Capacitación de Encuestadores 29
5.1. Preselección de los Encuestadores y supervisores 30
5.2. Estructura y Contenido de las Jornadas de Capacitación 32
5.3. Manual de trabajo de campo 33
6. Resultados del Trabajo de Campo 34
6.1. Control y seguimiento de la muestra 34
6.2. Incidencias del trabajo de campo 36
6.3. Productividad 37
7. Supervisión del Trabajo de Campo 38
8. Reporte Cualitativo del trabajo de campo 39
9. Depuración de Base de Trabajo de Campo 44
9.1. Ajuste por Factor de Expansión 46
10. Principales Resultados 47
10.1. Caracterización Demográfica de la Muestra 47
10.2. Resultados de Preguntas Sobre Hábitos de Consumo de Cigarrillos 50
10.3. Caracterización de Fumadores 60
10.4. Análisis Econométrico 76
11. Conclusiones 81
12. Anexos 84
12.1. Cuestionario 84
12.2. Hoja de ruta 88
12.3. Tabulación simple de los resultados 90

1. Presentación

El presente estudio fue dirigido por el Profesor Guillermo Paraje, de la Escuela de Negocios de la Universidad Adolfo Ibáñez, gracias al financiamiento otorgado por la American Cander Society y la Public Health School de la Johns Hopkins University, con la colaboración de Daniel Araya.[2] El trabajo de campo fue realizado por el Centro de Microdatos (CMD) de la Facultad de Economía de la Universidad de Chile. El objetivo principal del estudio es conocer los hábitos y patrones de consumo de los fumadores que residen en el Gran Santiago, así como indagar en torno al contrabando de cigarrillos y su magnitud en esta zona del país.

La importancia de esta investigación se fundamenta en la necesidad de entender el comportamiento de los fumadores y su interacción con el posible consumo de cigarrillos provenientes del contrabando. El contrabando implica la evasión de impuestos al tabaco y el debilitamiento de la política sanitaria basada en impuestos. El conocimiento de este fenómeno es útil para incrementar los controles de seguridad aduanera y comprender el comportamiento de los consumidores en relación a este fenómeno.

Para esto, se realizó una encuesta presencial en 40 puntos del Gran Santiago con gran afluencia de personas. La encuesta se realizó durante los meses de mayo y junio del 2017 e incluyó a 810 fumadores.

El presente informe contiene los siguientes aspectos de la investigación realizada:

- Diseño Muestral
- Diseño y programación del cuestionario
- Resultados del trabajo de campo
- Reporte Cualitativo del trabajo de campo
- Principales Resultados
- Anexos

[2] Se agradece la valiosa colaboración de Natalia Campos y Claudio Fuentes.

2. Acta de Aprobación de Comité de Ética

Con el fin de transparentar y cumplir con los requisitos éticos de los estudios del tipo del presente trabajo, se presentó el protocolo del proyecto al Comité de Ética de la Facultad de Economía y Negocios de la Universidad de Chile. A continuación, se adjunta la respuesta recibida:

FACULTAD
ECONOMÍA Y
NEGOCIOS
UNIVERSIDAD DE CHILE

ACTA DE APROBACIÓN COMITÉ DE ÉTICA DE INVESTIGACIÓN

IDENTIFICACIÓN DEL PROYECTO

**Proyecto:**	Encuesta de Consumo de Tabaco en la Región Metropolitana.
**Investigador responsable:**	Sandra Quijada.
**Unidad Prestadora del Servicio:**	Centro de Microdatos.
**Otra información relevante:**	Prestación de servicio de levantamiento de los datos requeridos para el estudio "Caracterización del Consumo de Tabaco en la Región Metropolitana"

DESCRIPCIÓN GENERAL DEL PROYECTO.

El Centro de Microdatos ha sido contratado por la Universidad Adolfo Ibáñez para realizar el servicio de levantamiento de "La Encuesta de Tabaco en la Región Metropolitana" cuyo objetivo es caracterizar a la población que fuma, hombres y mujeres de 15 años y más, respecto de su consumo habitual.
La encuesta será realizada en puntos de afluencia de personas que han sido seleccionados en forma aleatoria por la contraparte técnica y con una probabilidad conocida. Luego, los entrevistados serán seleccionados por cuotas (no probabilístico) de acuerdo a su sexo y edad.

La base de datos derivada de este servicio de recolección de datos se entregará innominada a la contraparte técnica para el análisis de los datos bajo su responsabilidad.
El Centro de Microdatos por acuerdo con la Universidad Adolfo Ibáñez no contempla la publicación y/o difusión de los datos resultantes del proceso de recolección de datos.

ABOGADO JEFE ★ UNIDAD JURÍDICA ★ FEN-U DE CHILE

EVALUACIÓN DE ASPECTOS ÉTICOS

Se deja constancia que el Comité de Ética para la Investigación de la Facultad de Economía y Negocios de la Universidad de Chile, en Sesión Ordinaria del 5 de Mayo del año en curso y Presidido por el Señor Decano, Prof. Manuel Agosin Trumper, y compuesto por:

- Valentina Paredes Haz, Phd., Ingeniero Comercial, Profesora Asistente del Departamento de Economía.
- Pedro Leiva Neuenschwander, Phd., Psicólogo, Profesor Asistente del Departamento de Administración.

Han revisado la documentación y los antecedentes presentados por Sandra Quijada, Jefa de Proyectos del Centro de Microdatos de la Facultad de Economía y Negocios de la Universidad de Chile, y referidos a los aspectos éticos del proyecto de prestación de servicios para el levantamiento de la información de la investigación "Encuesta de Consumo de Tabaco en la Región Metropolitana" dirigida por Guillermo Paraje, académico de la Universidad Adolfo Ibáñez, como consta en el contrato adjunto suscrito entre la Universidad Adolfo Ibáñez y la Facultad de Economía y Negocios.

Durante el proceso de revisión de la documentación presentada por la Jefa de Proyecto, los miembros de este comité han evaluado exhaustivamente el Protocolo de Investigación para Prestación de Servicios de Investigación presentado, así como Carta de Información al encuestado, concluyendo que:

(1) El proyecto para el cual se presta servicios de investigación tiene valor científico y social por cuanto sus resultados investigativos podrían contribuir al desarrollo del conocimiento científico y/o social.

(2) El protocolo presentado garantiza que los procedimientos de levantamiento de información y almacenamiento de datos, así como el compromiso asumido en la entrega de los informes a la institución contratante protege los derechos, dignidad y bienestar de los sujetos involucrados en la investigación, no constituyendo amenaza ni daño emocional ni moral.

(3) Se presentan de manera satisfactoria, el procedimiento para obtener el consentimiento informado.

(4) Se otorga a los participantes el derecho a abandonar el estudio sin ninguna consecuencia adversa, si lo consideraran necesario.

ABOGADO JEFE * UNIDAD JURIDICA * FEN-U. DE CHILE

(5) Se describe de manera satisfactoria los procedimientos que se implementarán para proteger la confidencialidad de la información recolectada de cada uno de los participantes.

(6) Se describe de manera satisfactoria las razones de inclusión y/o exclusión de determinados seres humanos.

(7) Se establece el compromiso de tomar acciones para responder por daños eventuales como resultado de la investigación, e informar a la brevedad a este Comité de esta situación, de modo de evaluar en conjunto si las acciones son suficientes o se requiere de acciones adicionales.

Dadas las circunstancias enunciadas anteriormente, el Comité de Ética para la Investigación de la Facultad de Economía y Negocios, declara no tener reparos en relación con la prestación de servicios de investigación y la califica como **Aprobada**, ya que da cumplimiento con los estándares y Normas Éticas para Investigación con Seres Humanos establecidas por la Organización Panamericana de la Salud de la Organización Mundial de la Salud, y de los principios explicitados en la Declaración Universal sobre Bioética y Derechos Humanos.

Se extiende el presente informe, en el entendido que con su recepción, el/la Jefe/a de Proyecto se obliga a cumplir con los requerimientos que le ha planteado este Comité, y no exceder en el desarrollo de su investigación el alcance de las consideraciones que han permitido emitirlo.

Manuel Agosin Trumper.
Decano y Presidente del Comité de Ética
Facultad de Economía y Negocios
UNIVERSIDAD DE CHILE

ABOGADO JEFE
UNIDAD JURIDICA
FEN-U DE CHILE

Santiago de Chile, 5 de Mayo de 2017

3. Diseño Muestral

El objetivo general del presente estudio es determinar los productos que consumen los fumadores, mayores de 13 años, residentes en la zona urbana de la Región Metropolitana. El tamaño de la muestra corresponde a 810 fumadores, los que fueron distribuidos en 40 puntos representativos de la distribución de población flotante en el área de estudio. Tales puntos fueron identificados en función de la concentración de construcciones destinadas a usos específicos. El procedimiento de selección consistió en identificar puntos de concentración con una dispersión geográfica representativa, donde fuese más probable encontrar personas que fumasen.

Con este objetivo, se desarrolló una metodología de muestreo aleatorio espacial, la cual consistió en la estimación del potencial de atracción de viajes de distintos tipos de edificación, la definición y cuantificación de áreas con flujo de público, seguido de la selección aleatoria de estas áreas e identificación cartográfica de los puntos de mayor atracción en las áreas seleccionadas.

3.1. Metodología de muestreo para implementación de la encuesta en población flotante[3]

A continuación, se detallan cada una de las fases asociadas a la determinación de los puntos de afluencia seleccionados en la muestra, la determinación de cuotas y el entrevistado final.

[3] La documentación del diseño muestral y selección de los 40 puntos para la realización de la encuesta fue provista por: Matías Garretón, Luis Valenzuela y Ricardo Truffello del Centro de Inteligencia Territorial, Universidad Adolfo Ibáñez.

3.1.1. Estimación de la atracción de viajes.

La estimación del potencial de atracción de población flotante que tienen distintos tipos de edificación se basa en datos de la Encuesta Origen y Destino del Gran Santiago 2012 (EOD), y en el catastro de superficies construidas del Servicio de Impuestos Internos, actualizado a 2016 (SII), combinados a nivel de 767 zonas definidas en la EOD. Para cada zona, se calculó el total de viajes atraídos diariamente (excluyendo el motivo de retorno a domicilio) y el total de m^2 construidos según los 22 usos definidos por el SII (excluyendo los usos habitacionales y bienes comunes). Estas exclusiones responden a que interesa identificar los atractores de viajes en los que se distribuye la población flotante de forma independiente a los generadores de viajes (donde se localiza la población residente).

Con dicha información se realizó una regresión multivariada usando como variable dependiente la cantidad de viajes atraídos y como variables explicativas los usos construidos, sin considerar el intercepto. Cabe destacar que se excluyeron los usos no significativos hasta obtener el modelo descrito en la Tabla 1.

Tabla 1: Atracción de viajes en función de tipos de construcción

```
Call:
lm(formula = N ~ C + D + E + O + Q + S + T + AV + 0, data = atractores)

Residuals:
   Min     1Q Median     3Q    Max 
-24874  -1597    734   3889  42256 

Coefficients:
   Estimate Std. Error t value             Pr(>|t|)    
C  0.168494   0.009747  17.287 < 0.0000000000000002 ***
D  0.249856   0.118444   2.109             0.035230 *  
E  0.144557   0.017630   8.200  0.00000000000000103 ***
O  0.063552   0.005316  11.954 < 0.0000000000000002 ***
Q  0.671209   0.149025   4.504  0.00000771772112184 ***
S  0.140289   0.030002   4.676  0.00000346336574362 ***
T  0.268148   0.079904   3.356             0.000831 ***
AV 0.015237   0.003060   4.980  0.00000078772107454 ***
---
Signif. codes:  0 ‘***’ 0.001 ‘**’ 0.01 ‘*’ 0.05 ‘.’ 0.1 ‘ ’ 1

Residual standard error: 6825 on 759 degrees of freedom
Multiple R-squared:  0.781,	Adjusted R-squared:  0.7787 
F-statistic: 338.4 on 8 and 759 DF,  p-value: < 0.00000000000000022
```

Nota: variables consideradas en el modelo

N: número de viajes

C: m^2 comercio

D: m^2 deporte

E: m^2 educación y cultura

O: m^2 oficinas

Q: m^2 culto

S: m^2 salud

T: m^2 transporte y telecomunicaciones

AV: m^2 área verde

Con el modelo de la Tabla 1 se estimó el potencial de atracción de viajes a nivel de las 66.467 manzanas catastradas por el SII en función de las superficies construidas en cada una y de los coeficientes correspondientes a cada tipo.

3.1.2. Definición y cuantificación de áreas de flujo.

Para obtener un universo de muestreo con una buena dispersión geográfica, se identificó a las manzanas cuyo potencial de atracción es mayor a sus vecinas, apuntando a obtener un total de polos de atracción algo mayor a 400, número adecuado para obtener 40 lugares de muestreo (tasa de selección=1/10), repartidos de forma regular en el área urbana. La definición de un criterio de 160 vecinos más cercanos permitió identificar 424 polos locales de atracción (pese a que 66.467/160=415, se obtuvo un número mayor debido a la irregularidad geométrica de las manzanas).

El resto de las manzanas se identificó con el polo local más cercano a cada una, lo que permitió definir áreas aproximadamente circulares, las cuales cubren la totalidad del área urbana catastrada por el SII de forma unívoca y no redundante. Para cada área, se calculó el potencial total de atracción de población flotante, sumando los de las manzanas que la componen, y que fueron previamente estimados.

3.1.3. Selección aleatoria de áreas de flujo.

Del total de 424 áreas de flujo, se seleccionó una muestra de 40 zonas, mediante un procedimiento de selección aleatoria con probabilidad proporcional al tamaño (PPS), considerando que éste corresponde al potencial total de atracción de población flotante de cada área. Este punto es relevante, ya que la unidad de selección es el área y no una manzana específica dentro de ella. Debido a su tamaño, un conjunto de áreas centrales de alta densidad tuvo una probabilidad de selección de 100% y un subconjunto de éstas se repetía en la selección. En consecuencia, se utilizó un tamaño muestral objetivo de 43, obteniéndose una muestra final de 40 áreas luego de eliminar las repeticiones.

La Figura 1 muestra la distribución geográfica de los atractores locales que definieron el universo de selección y los centros de áreas seleccionadas en la muestra. Como se utilizó una selección aleatoria dependiente del tamaño, la muestra refleja una alta concentración de edificaciones consideradas como atractores de viajes en la zona central del Gran Santiago, en el eje centro-nororiente y en el eje norte-sur, aunque también contiene puntos en zonas periféricas con estimaciones de carga relativamente bajas. En contraparte, las zonas más extensas sin puntos seleccionados son aquellas donde existe una alta especialización residencial y, por lo tanto, un menor potencial de atracción de población flotante.

Figura 1: Atractores locales y centros de áreas seleccionadas en el Gran Santiago

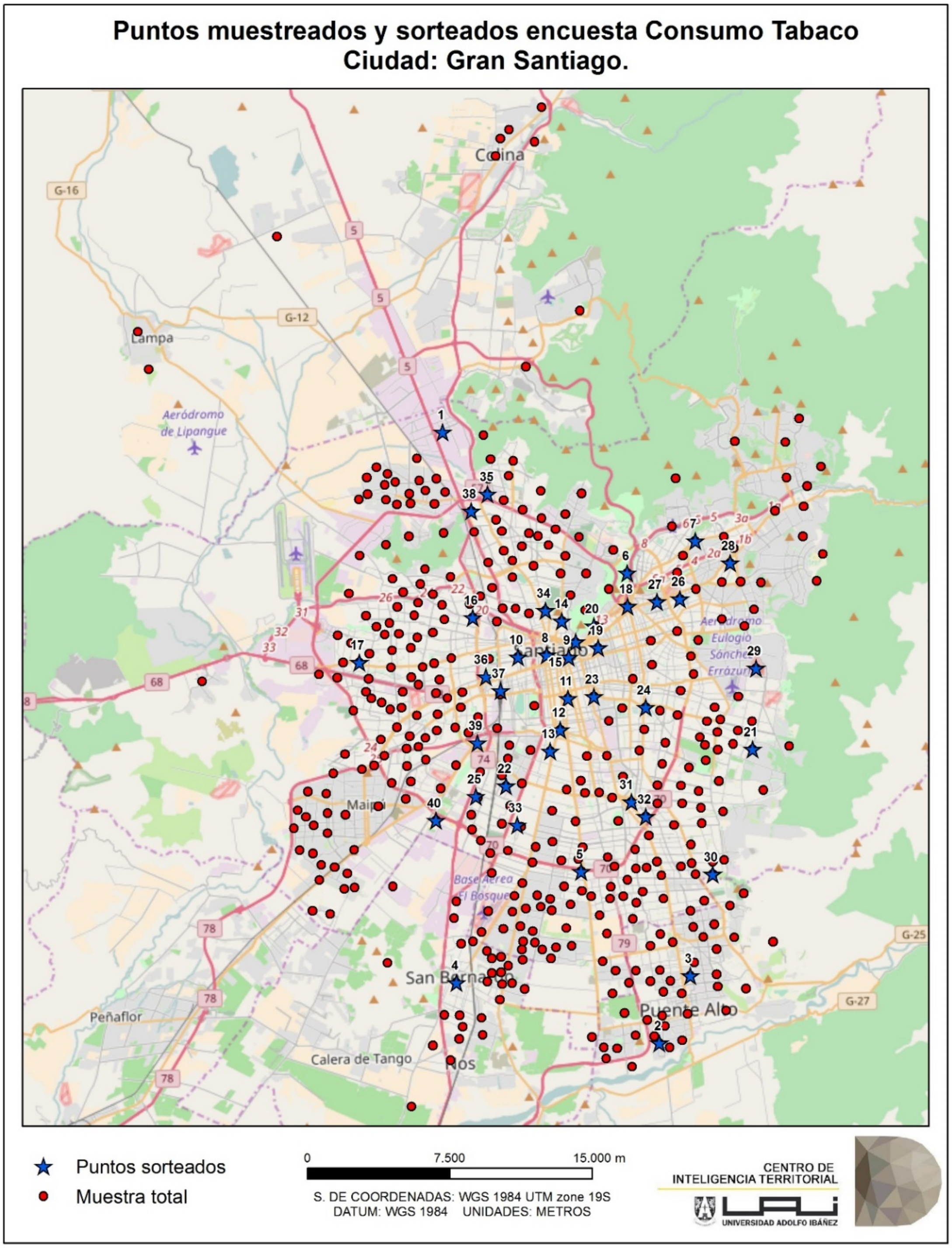

3.1.4. Representación cartográfica de los puntos de mayor atracción en las áreas seleccionadas.

En cada una de las áreas seleccionadas se identificó la manzana con mayor potencial de atracción de flujo de público, la que puede corresponder o no, a la manzana identificada originalmente como un polo local. Cabe destacar que la definición inicial de polos locales responde al objetivo de generar un universo de puntos con una buena dispersión y cobertura de la ciudad, pero que la selección aleatoria se realizó a nivel de áreas y, por lo tanto, no hay una restricción a priori de cuál debe ser la coordenada de referencia para encuestar cada área.

Por motivos de representatividad, y también pragmáticos, se tomó como referencia la manzana con mayor capacidad de atracción en cada área. Por un lado, porque es el lugar que tiene la mayor probabilidad de encontrar a una persona que accede al área en cuestión. Y, por otro lado, porque, al esperarse en ella la mayor concentración de flujos se favorece la realización exitosa del proceso de levantamiento de encuestas.

Las manzanas seleccionadas bajo este método y su entorno inmediato fueron debidamente representadas en una serie de mapas correspondientes (ejemplo en Figura 2), en los que se identificó también una referencia de transporte según dos criterios. Primero, se tomó como referencia una estación de metro si existía alguna en el entorno de la manzana. Segundo, si no hay estaciones de metro próximas, se tomó como referencia la intersección de dos calles relativamente importantes en el perímetro de la manzana. Cabe señalar que las infraestructuras de transporte no han sido consideradas en sí mismas como atractores de viajes, ya que son lugares de paso, sin importar lo intenso que sea el flujo que reciben. En este sentido, para evitar dobles cuentas de la magnitud que determina la probabilidad de selección, fue preferible incluirlas dentro de una relación funcional a un atractor final de viajes, posibilitando así la aplicación de encuestas en ellas sin introducir distorsiones en el proceso de selección.

Considerando lo anterior, y el patrón usual de desplazamiento peatonal que tiende a conectar una estación de metro o una intersección importante con edificaciones atractoras de viajes, se recomendó el siguiente criterio para el trabajo de campo. "El encuestador debiese llegar al punto identificado como referencia de transporte y comenzar el proceso de levantamiento de información en el trayecto entre esta y la manzana principal. En seguida, el encuestador puede recorrer el perímetro de esta manzana. Si, pese a ello, no se obtuviera el número de encuestas necesarias, el encuestador podrá explorar los alrededores con bastante libertad, en un radio de 500 metros alrededor de la manzana principal, ya que este espacio también forma parte de la unidad seleccionada".

Figura 2: Mapa referencial de una zona de trabajo de campo

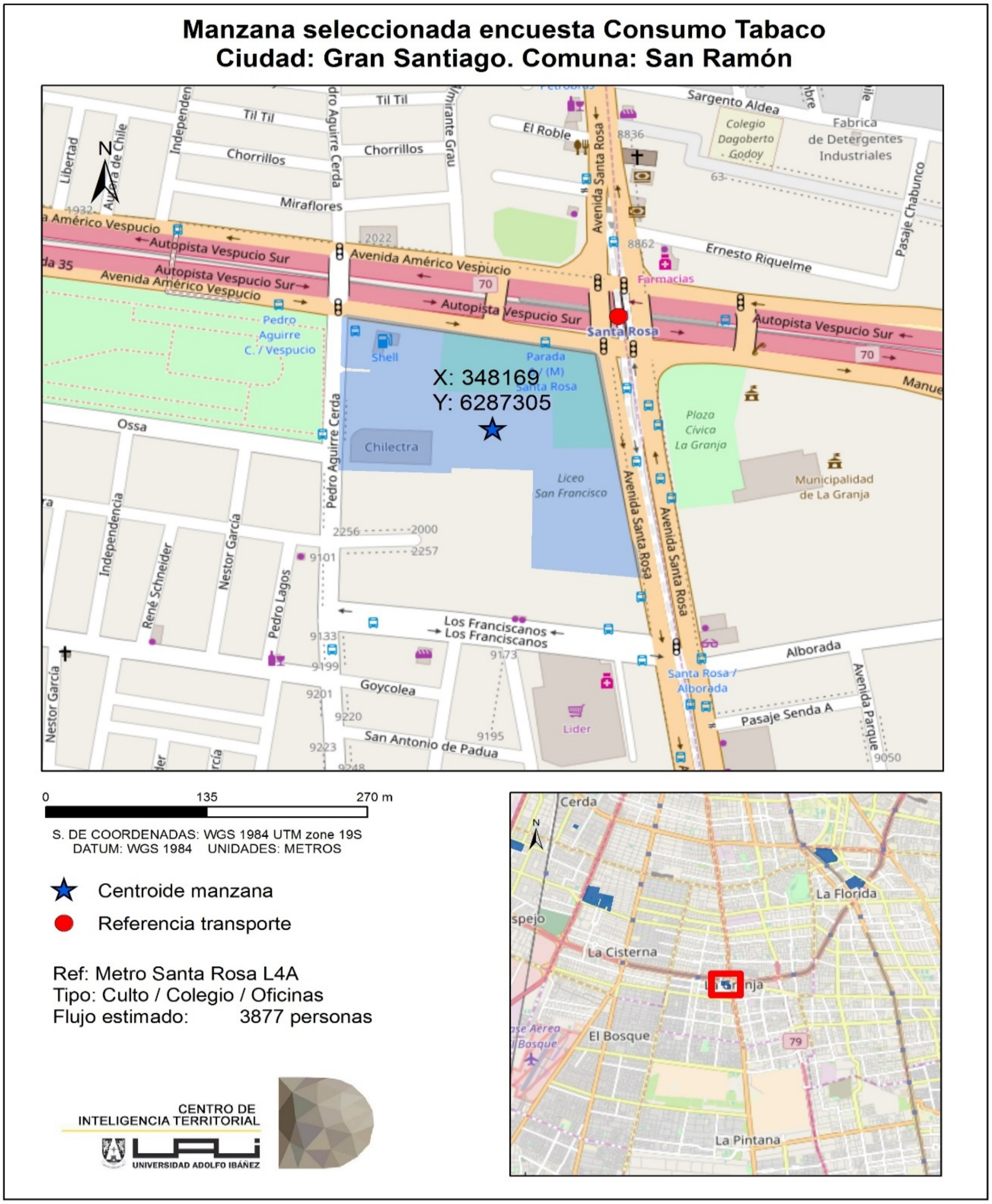

3.1.5. Determinación de las características de las personas a encuestar

El Centro de Inteligencia Territorial de la UAI facilitó los puntos de afluencia de público para la selección de los entrevistados, conforme a ello se determinó encuestar a 20 personas por punto de afluencia. Luego, para definir las cuotas, se tomaron como referencia los resultados de prevalencia en el consumo de tabaco de la última base de datos de la encuesta a población general disponible en la página web de SENDA.

El análisis de la base de datos se realizó por sexo, edad y comuna, dando origen a la distribución. Finalmente, la selección definitiva del entrevistado se realizó in situ en el punto de afluencia sobre personas que estuvieran fumando.

Tabla 2 – Distribución de la Muestra y cuotas asociadas al número de entrevistados

Punto Geográfico	Comuna	Hombres				Total Hombres	Mujeres				Total Mujeres	TOTAL
		18 y menos años	19 a 40	40 a 60	61 y más		18 y menos años	19 a 40	40 a 60	61 y más		
Autopista Aconcagua esq. Av San Ignacio	Quilicura	3	3	2	1	9	4	4	2	1	11	20
Av. Ejercito Libertador esq. Patria Nueva	Puente Alto	3	3	2	1	9	3	4	3	1	11	20
Av. Las Nieves esq. Nemesio Vicuña	Puente Alto	3	3	3	1	10	3	3	3	1	10	20
San José esq. Eyzaguirre	San Bernardo	2	3	3	1	9	2	4	3	2	11	20
Metro Santa Rosa L4A	San Ramón	2	3	3	2	10	3	3	2	2	10	20
Plaza Antilen	Vitacura	2	4	2	1	9	3	4	2	2	11	20
Av. Vitacura esq. Embajador Doussinage	Vitacura	2	3	2	2	9	3	3	3	2	11	20
Agustinas esq. Morande	Santiago	1	6	2	1	10	1	6	2	1	10	20
Metro Universidad Católica L1	Santiago	1	6	2	1	10	1	6	2	1	10	20
Cumming esq. Compañía de Jesús	Santiago	1	4	3	1	9	2	5	3	1	11	20
Av. Santa Rosa esq. Amazonas	Santiago	2	5	2	1	10	2	5	2	1	10	20
San Diego esq. Placer	Santiago	2	4	3	1	10	2	4	3	1	10	20
Metro San Miguel L2	San Miguel	2	3	3	1	9	2	4	3	2	11	20
Metro Cerro Blanco L2	Recoleta	2	5	2	1	10	2	5	2	1	10	20
Bellavista esq. Pio Nono	Recoleta	1	6	2	1	10	1	6	2	1	10	20
Carrascal esq. Aviador Bleriot	Quinta Normal	2	3	3	2	10	2	3	3	2	10	20
San Pablo esq. Santa Victoria	Pudahuel	3	4	2	1	10	3	4	2	1	10	20
Av. Vitacura esq. Nueva Tobalaba	Providencia	1	5	2	1	9	1	6	2	2	11	20
Metro Salvador L1	Providencia	1	5	2	1	9	1	6	2	2	11	20
Bellavista esq. Francisco Puelma	Providencia	1	4	2	2	9	2	5	2	2	11	20
Las Perdices esq. Antupiren	Peñalolén	3	2	4	1	10	3	2	4	1	10	20
Alhue esq. Quemchi	Pedro Aguirre Cerda	2	3	3	2	10	2	3	3	2	10	20
Av. Vicuña Mackenna esq. Victoria	Ñuñoa	1	4	3	1	9	2	4	3	2	11	20
Av. Grecia esq. Av. José Pedro Alessandri	Ñuñoa	2	3	3	1	9	2	4	3	2	11	20

Punto Geográfico	Comuna	Hombres				Total Hombres	Mujeres				Total Mujeres	TOTAL
		Menores de 18 años	19 a 40	40 a 60	61 y más		Menores de 18 años	19 a 40	40 a 60	61 y más		
Av. Cardenal Raúl Silva H. esq. Buenaventura	Lo Espejo	2	3	3	2	10	2	3	3	2	10	20
Av. Apoquindo esq. Puerta del Sol	Las Condes	1	4	2	2	9	2	5	2	2	11	20
Metro Alcántara L1	Las Condes	1	4	2	2	9	2	5	2	2	11	20
Av. Las Condes esq. Las Azaleas	Las Condes	2	3	3	1	9	2	4	3	2	11	20
Av. Alc. Fdo. Castillo V. esq. Valenzuela Llanos	La Reina	3	4	2	1	10	3	4	2	1	10	20
Av. La Florida / Santa Amalia	La Florida	3	3	3	1	10	3	3	3	1	10	20
Metro Mirador L5	La Florida	2	4	2	1	9	2	5	3	1	11	20
Metro Bellavista de la Florida L5	La Florida	1	4	3	1	9	2	4	3	2	11	20
Av. El Parrón esq. La Paz	La Cisterna	2	4	2	1	9	2	5	3	1	11	20
Av. Independencia esq. Av. Santos Dumont	Independencia	2	4	2	1	9	2	5	3	1	11	20
Av. Américo Vespucio esq. Los Libertadores	Huechuraba	2	3	3	1	9	2	4	3	2	11	20
Av. Ecuador esq. Enrique Kirberg	Estación Central	2	4	2	1	9	2	4	3	2	11	20
Metro Estación Central L1	Estación Central	2	4	3	1	10	2	4	3	1	10	20
Av. Am. Vespucio N. esq. Av. Pdte. Eduardo Frei M.	Conchalí	3	3	2	2	10	3	3	2	2	10	20
Av. Pedro Aguirre Cerda esq. Buzeta	Cerrillos	3	3	2	2	10	3	3	2	2	10	20
Autopista Vespucio Sur esq. Camino a Lonquen	Cerrillos	2	3	3	2	10	3	3	3	1	10	20
**Total**		**78**	**151**	**99**	**51**	**379**	**89**	**167**	**104**	**61**	**421**	**800**

4. Diseño y Programación del Cuestionario

El cuestionario, que se adjunta en el anexo, consta 25 preguntas, adaptadas de un estudio similar realizado en Colombia durante 2016. Las adaptaciones fueron:

1. Se hicieron cambios en la introducción y presentación de la encuesta de acuerdo a las recomendaciones del comité de ética de la Facultad de Economía y Negocios de la Universidad de Chile, que evaluó y respaldó el levantamiento de información de este proyecto.
2. En la pregunta ¿Usted reside en esta ciudad? Se modificó indicando si la persona residía en Santiago, ya que uno de los requisitos para participar en la encuesta es que fuese residente de la zona urbana de la Región Metropolitana.
3. En la pregunta 8 se adecua el texto a la pertinencia de Chile.
4. En la pregunta 9 se incorporó un texto de introducción para las preguntas siguientes
   "Ahora le hare unas preguntas sobre los lugares que venden cigarrillos, sobre la marca de su última compra y los precios de esa compra".
5. Se incorpora una pregunta para definir si el entrevistado es Asalariado o Independiente.
6. No se incluyen preguntas relativas a ingresos o tramos de ingresos de los entrevistados.

Consiguientemente, dicho cuestionario se programó en un software de diseño de cuestionarios desarrollado por el CMD desde 2012. El documento final se adjunta digitalmente en los anexos de este informe.

La programación de la encuesta en Tablet estuvo a cargo de un equipo experto en desarrollo informático, específicamente en software para la aplicación de encuestas en Tablet y la correspondiente gestión de éstas.

El diseño de esta aplicación permite:

- Que el encuestador trabaje de manera rápida y simple para acortar los tiempos de entrevista.
- Obtener datos más confiables, en la medida que la malla de validación que es aplicada durante la entrevista sea completa y esté bien definida.
- La malla de validación se haga cargo del control de saltos condicionados de preguntas en función de las respuestas anteriores, para que las respuestas se limiten al recorrido definido para cada pregunta y exista coherencia en las respuestas a preguntas relacionadas, alertando al encuestador ante respuestas fuera de rango o inconsistentes.
- Suspender temporalmente la entrevista y reanudarla en un momento posterior de acuerdo a la disponibilidad del entrevistado.
- La visualización de la hoja de ruta con toda la información requerida para identificar al hogar y/o al entrevistado.
- Llevar un registro en línea sobre el avance del trabajo en terreno, de manera de contar con las alertas oportunas frente a cualquier dificultad.
- Medir el tiempo de aplicación del instrumento, entregando la fecha y hora en que fue realizada la entrevista[4].
- Registrar información del cuestionario, de las condiciones de aplicación y los resultados de las visitas. El día y hora asociado a ello son generados de manera automática en el momento en que el encuestador seleccione un determinado hogar (o entrevistado) y su Código de Disposición Final.
- Registro del encuestador asociado a la encuesta[5].
- Agregar nuevas direcciones o actualizar la dirección existente y los datos de contacto con el entrevistado.

---

[4] Todas las acciones realizadas en la Tablet cuentan con un registro de horario, incluso para cada una de las preguntas que componen el cuestionario.

[5] Cada encuestador tiene asignada una Tablet y una clave de acceso, por tanto, toda la información que registre en relación a un determinado entrevistado le será imputado automáticamente a dicho encuestador, lo que permite monitorear de manera directa el trabajo de cada integrante del equipo en tiempo real.

A continuación, se presentan imágenes que ejemplifican la forma en que se presentó la información para los encuestadores en la Tablet.

Ilustración 1: Ejemplo de Hoja de Ruta en Tablet

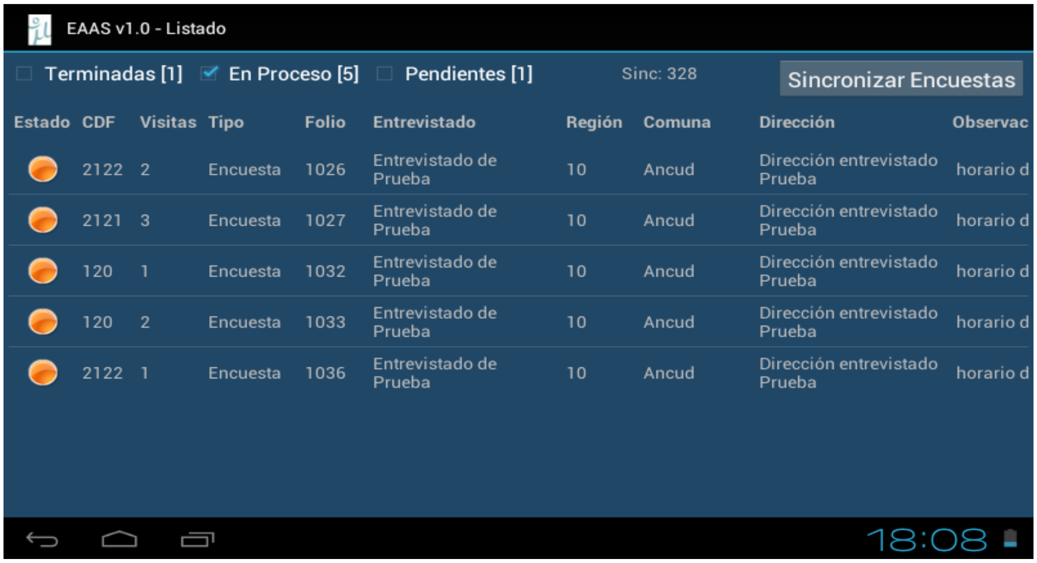

Ilustración 2: Acceso a una imagen con el mapa asociado a la dirección

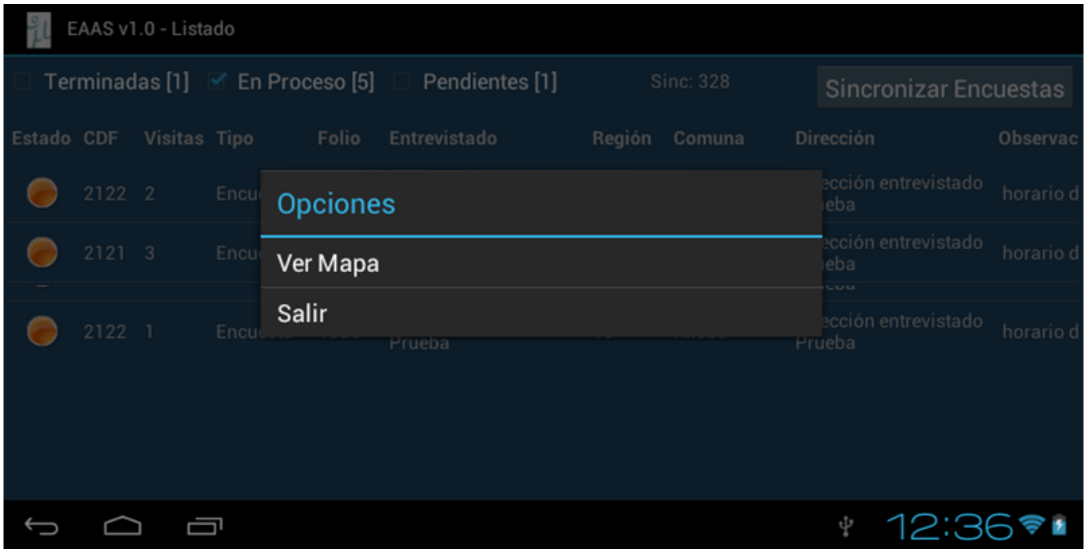

Ilustración 3: Detalle de los intentos de contacto realizado en un determinado hogar

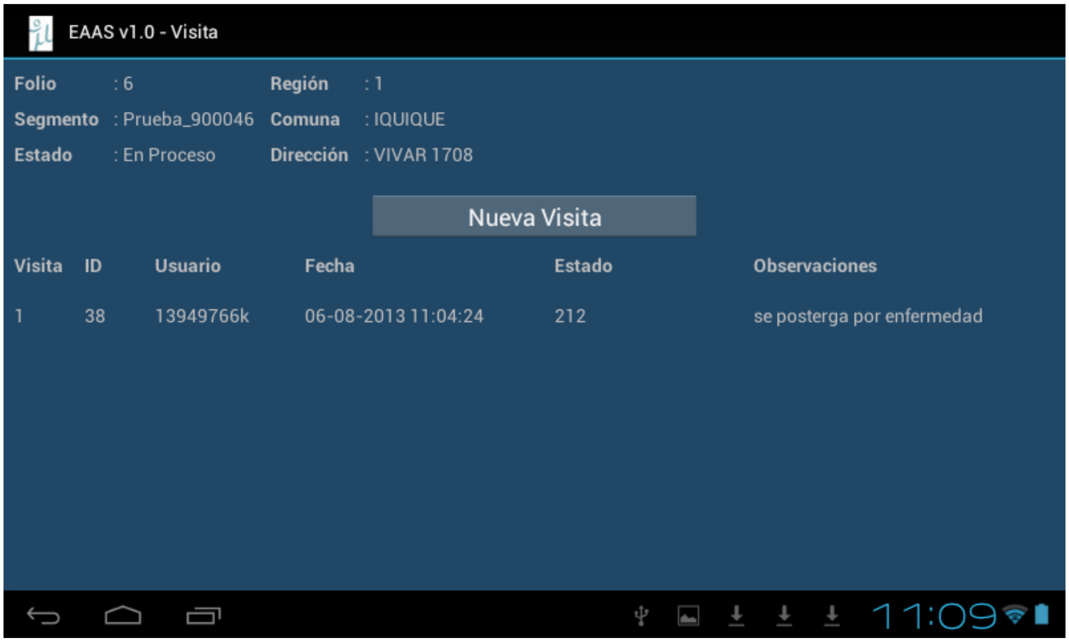

Ilustración 4: Ejemplo de presentación de preguntas-respuesta única

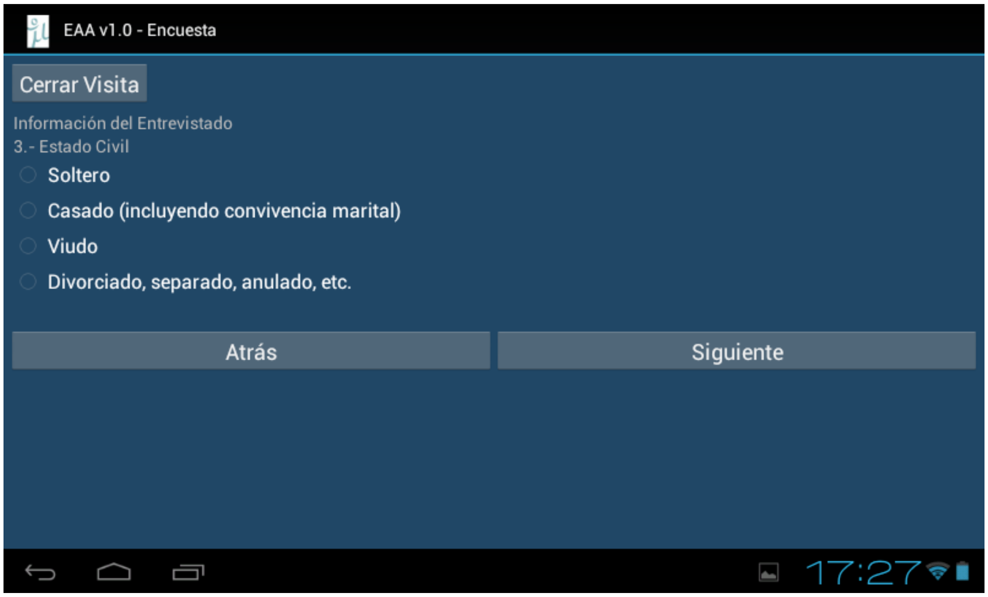

Ilustración 5: Ejemplo de presentación de preguntas-respuesta única

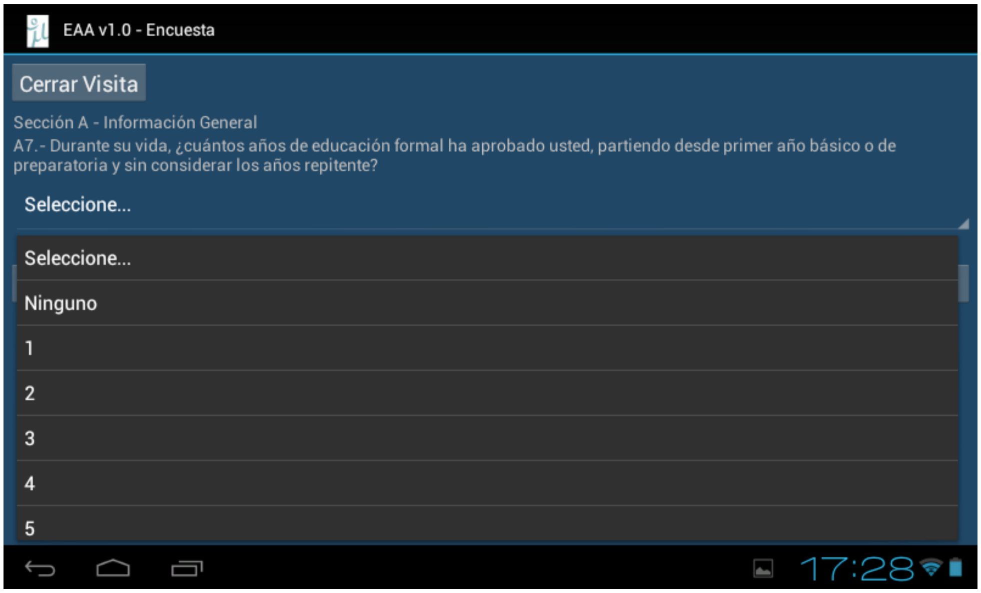

Ilustración 6: Ejemplo de presentación de preguntas-respuesta abierta

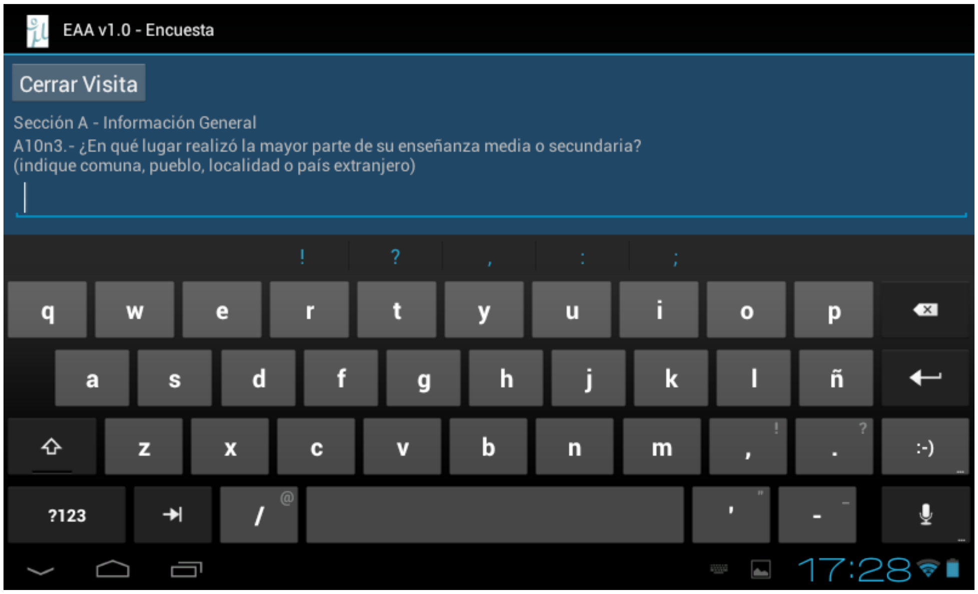

Ilustración 7: Ejemplo de validación de respuestas

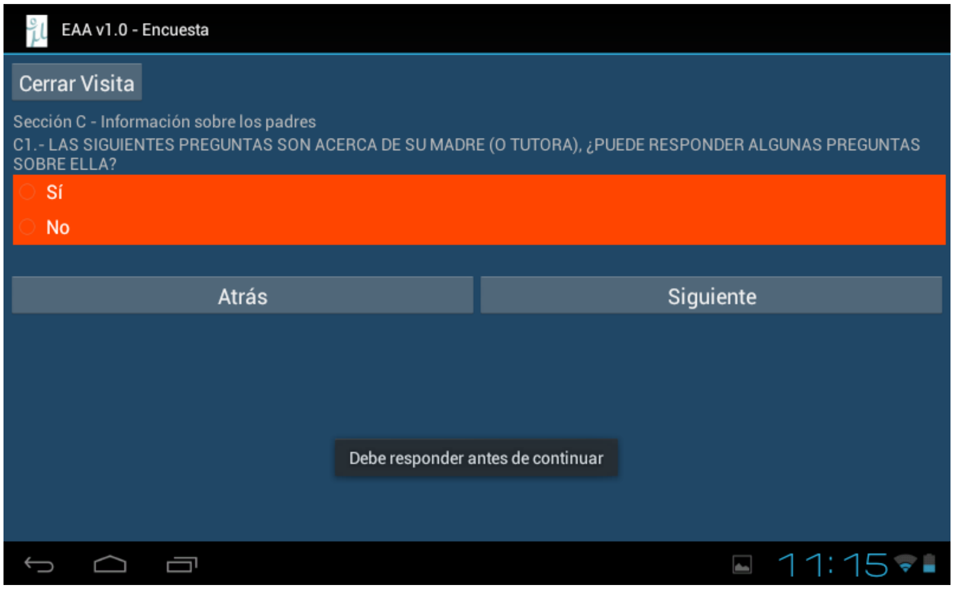

La programación de la Tablet fue revisada sistemáticamente por el equipo investigador y de validación con el fin de asegurar la inexistencia de divergencias con el cuestionario en papel, problemas de flujo o saltos erróneos. Además, la debida programación permitió retroceder durante la encuesta, sin borrar la información ya recolectada. De igual forma, la información ya recolectada durante una entrevista quedó almacenada en el disco duro de la tablet aunque esta se hubiese apagado por problemas técnicos.

5. Reclutamiento y Capacitación de Encuestadores

Este proceso se inició con la publicación del formulario de inscripción de encuestadores en el sitio web del CMD. En forma paralela, se invitó a participar a los encuestadores que, previamente, tuvieron un buen desempeño en las últimas encuestas levantadas en los últimos 5 años. El CMD cuenta con una página web

donde se publican todos los estudios que están en curso de levantamiento. Esta página principal cuenta con una página especial de oportunidades de trabajo, en la cual se puede verificar la información de cualquier encuestador que esté trabajando con la Unidad de Encuestas en alguno de sus diversos proyectos.

Ilustración 8: Verificación de antecedentes de los encuestadores.

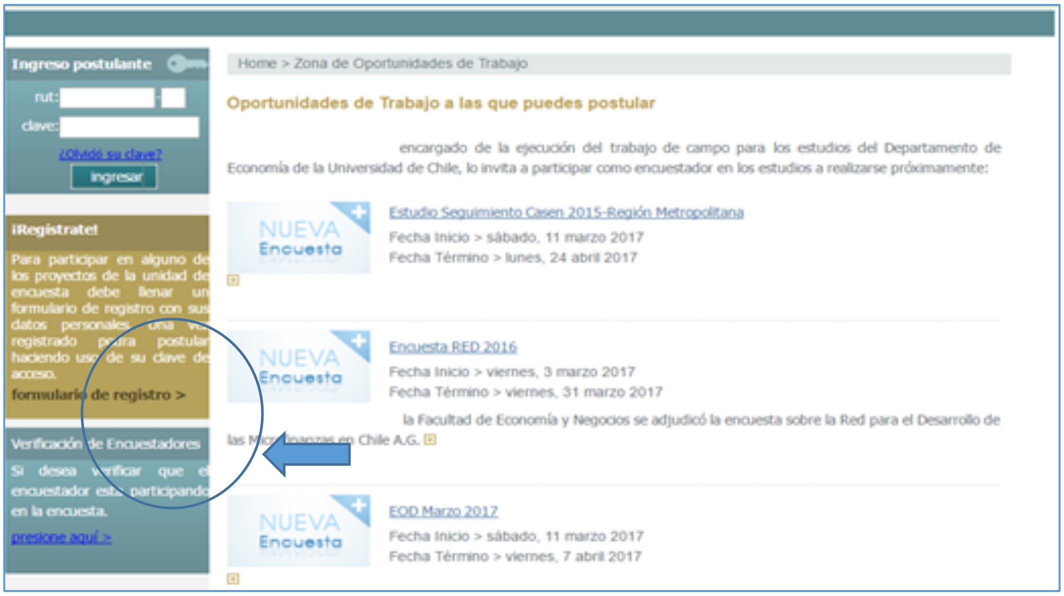

Dado el tamaño de la muestra no fue necesario realizar una difusión más amplia, en suma, los encuestadores con experiencia y que trabajan habitualmente con el CMD fueron seleccionados para el trabajo de campo de este proyecto.

5.1. Preselección de los Encuestadores y supervisores

Para cumplir con los objetivos de la encuesta, se requirió contar con encuestadores y supervisores con una vasta experiencia en encuestas a individuos, consecuentemente, se privilegió la experiencia en encuestas cara a cara en puntos con afluencia de público. Para cumplir con esta exigencia se consideró reclutar encuestadores del staff central. Específicamente, se consideró a personas que

realizaron esta labor previamente y que tuvieron un desempeño destacado en Trato Usuario del Ministerio de Salud 2016, Encuesta Casen, Encuesta COES, Encuesta Jóvenes, Encuesta Consumo de Tabaco y en otros estudios relevantes ejecutados por nuestra Unidad de Encuestas durante los últimos tres años.

Todo el personal preseleccionado y dispuesto, asistió a la jornada de capacitación donde recibieron el manual del encuestador en formato digital (sin anexos) con el objetivo de que su contenido fuese revisado con anterioridad a su asistencia a la jornada de trabajo.

La capacitación de los encuestadores es una de las etapas fundamentales de los procesos de levantamiento de información a través de las encuestas. Según la literatura de metodología de encuestas[6] uno de los errores más recurrentes no asociados a la muestra, es el error de medición en que incurre el encuestador, que por no recibir las indicaciones adecuadas o por no tener una buena supervisión, induce al entrevistado a errores al responder o bien incurre en errores al codificar en terreno las respuestas que está entregando el entrevistado al momento de la entrevista.

Conscientes de la importancia de este tema, se realizó un proceso de capacitación estandarizado, el que fue ejecutado por personal con experiencia en relatorías y selección de encuestadores del CMD. Estas capacitaciones se llevaron a cabo en la sede central del CMD, las que fueron financiadas y ejecutadas en su totalidad por este. En estas capacitaciones se consideró la participación de la contraparte técnica quien fue invitada a participar.

[6] Ver Biemer, P. P;Lyberg, L.E (2003). Introduction to Survey Quality. John Willey and Sons, New Jersey.

5.2. Estructura y Contenido de las Jornadas de Capacitación

Las jornadas de capacitación requeridas tuvieron una duración entre 4 a 6 horas. La primera capacitación fue trabajada con la Contraparte Técnica; esta capacitación se realizó previo al levantamiento definitivo y estuvieron presentes todos los integrantes del equipo de encuestadores, junto con los supervisores y todo aquel profesional que formó parte del trabajo de campo. Esta capacitación cubrió todos los aspectos técnicos y operativos del trabajo de campo incluyendo la práctica con las computadoras portátiles.

La distribución de los contenidos específicos tratados se presenta a continuación:

Tabla 3: Programa Jornada Capacitación para Encuestadores

Día	Horario	Temas a tratar
**Día 1**	09.00 a 11.00	Registro de participantes.<br>Presentación del estudio (objetivos de la investigación, muestra, contexto en que se realiza la aplicación, estructura y contenido del cuestionario).<br>Temas Organizacionales y de trabajo.<br>Actividades generales que comprende el levantamiento.<br>Condiciones de trabajo (perfil, rol del encuestador, honorarios, estrategias de selección, entre otros).
	11.00 a 11.30	Café
	11.30 a 13.00	Protocolo de aplicación (Etapas específicas para ejecutar el levantamiento: Retiro de material, técnicas de acercamiento, protocolo de contacto, indicaciones para la aplicación instrumento, estrategias de motivación al entrevistado y notificación de avances.).

		Potenciales dificultades a enfrentar y propuesta de soluciones.
	13.00 a 14.30	Almuerzo
	16.30 a 18.30	▪ Role Playing (simulación de contacto con el entrevistado)<br>▪ Práctica Tablet

En todos los levantamientos de información se generan rotaciones del personal del trabajo de campo (encuestadores y supervisores), por tal razón, posteriormente se generó solo un nuevo proceso de capacitación, el cual tuvo el mismo estándar que su antecesor en lo que respecta a la entrega de información, cubriendo aspectos técnicos y operativos del trabajo de campo.

5.3. Manual de trabajo de campo

Como complemento al trabajo de campo de los encuestadores y supervisores se desarrolló un manual de trabajo de campo, el que contiene los siguientes ítems:

- Objetivos del estudio
- Labores del cargo
- Técnicas para abordar al encuestado (protocolos)
- Descripción de la muestra
- Cuestionario
- Uso de la Tablet

El manual desarrollado para la implementación del trabajo de campo se adjunta en formato digital como adjunto al presente informe.

6. Resultados del Trabajo de Campo

A continuación, se presentan los principales resultados del trabajo de campo que fue realizado entre el 1 de Junio y el 17 de Julio de este año. Durante este tiempo el equipo de encuestadores estuvo compuesto por ocho encuestadores, siete de ellos mujeres y un hombre.

El logro de la muestra fue de un 7% superior a lo requerido, para llegar a este resultado se llevó un control y seguimiento de la muestra mediante nuestro sistema de gestión de encuestas, los resultados se evidencian a continuación.

6.1. Control y seguimiento de la muestra

Al igual que para todos los proyectos del CMD se generó una página especial de gestión para esta encuesta, donde se observaba el avance diario del logro de dichas entrevistas, revisión de las mismas, supervisión y estimación de tiempos de aplicación, entre otros aspectos propios de la operación del trabajo de campo.

Los resultados del trabajo de campo se traducen en un cuadro de avance que indica el logro en las cuotas determinadas para la definición de la muestra. El resultado final de cumplimiento se muestra en la Tabla 4:

Tabla 4: Logro por celda definida por sexo y tramo de edad

						Sexo					
				Masculino					Femenino		
				Tramos edad					Tramos edad		
	**H menores de 18**	**H 18 a 40**	**H 41 a 60**	**H 61 y más**	Total Hombre	**M menores de 18**	**M 18 a 40**	**M 41 a 60**	**M 61 y más**	Total Mujer	Total general
Segmento	1	2	3	4		1	2	3	4		
1	2	4	4	1	11	1	4	4	1	10	**21**

2	1	6	5	1	13	7	2	3	2	14	**27**
3	1	4	4	1	10	4	3		1	8	**18**
4	2	5	2	1	10	2	7	3	1	13	**23**
5	1	4	4	1	10	2	3	4	1	10	**20**
6		4	2	1	7		2	2		4	**11**
7	1	3	4	2	10		3	5	2	10	**20**
8	1	5	5	2	13		6	2		8	**21**
9		5	3	1	9		3	4	1	8	**17**
10	1	7	5	1	14	1	4	2	1	8	**22**
11	1	4	5	1	11	1	5	3	1	10	**21**
12	1	5	5	1	12	1	5	1	1	8	**20**
13	7	8	12	1	28	4	18	5	6	33	**61**
14		6	1	1	8		3	2	4	9	**17**
15	2	6	1	1	10	1	4	2	4	11	**21**
16	1	4	4	2	11	1	3	5	1	10	**21**
17	1	4	4	2	11	1	4	5		10	**21**
18	1	4	6	1	12	1	4	6	1	12	**24**
19	1	4	6	1	12	1	4	5	1	11	**23**
20	1	4	6	1	12	1	5	5	1	12	**24**
21		3	1		4	1	3	6		10	**14**
22	1		1	4	6		1	1		2	**8**
23	2	5	5	1	13	2	5	5	1	13	**26**
24		4	5	1	10	1	4	4	1	10	**20**
25	1	5	4	1	11	1	4	4	1	10	**21**
26	1	6	4	1	12	1	4	5	1	11	**23**
27		3	2	1	6	1	4	3		8	**14**
28		7	4	3	14		4	7	1	12	**26**
29	1	4	4		9	1	4	4	2	11	**20**
30		1	6	1	8	1	1	5	1	8	**16**
31	2	2	8	1	13	1	6	7	1	15	**28**
32	1	3	6	1	11	1	4	5	1	11	**22**
33	1	5	3	1	10	1	6	4		11	**21**
34		3	2	2	7	1	3	6	2	12	**19**
35	1	5	4	1	11	1	5	5	2	13	**24**
36	1	5	3	1	10	1	5	3	1	10	**20**
37	1	7	4	2	14	1	5	3	1	10	**24**
38	2	5	2	1	10	1	4	4	1	10	**20**
39	1	4	2		7	1	3	3		7	**14**
40	1	5	4	1	11	1	6	5	1	13	**24**
Total general	43	178	162	48	431	48	173	157	48	426	**857**
Muestra	**45**	**154**	**158**	**44**	**401**	**53**	**164**	**134**	**48**	**399**	**800**

6.2. Incidencias del trabajo de campo

En el protocolo de trabajo de campo se le indicaba al encuestador que registrase cada intento de contacto con el posible entrevistado, para ello se adaptaron a este levantamiento los siguientes estados de registro de incidencias:

Tabla 5: Código disposición final de casos

Códigos para registro de intentos de entrevista.
510 = Sin CDF
110 = Entrevista Completa
111 = Entrevista Terminada que no sirve
120 = Entrevista Parcial
210 = No lograda, se negó el entrevistado
220 = No lograda, se negó el entrevistado rotundamente
230 = No lograda, se negó un familiar del entrevistado
233 = Problemas de idioma
300 = No lograda, por otras razones. Especifique

Luego, durante el trabajo de campo se dio la instrucción de que la encuesta sería válida solo en la medida que esta incluyera el consentimiento del entrevistado para tomar la fotografía de la cajetilla de cigarrillos. Dado esto, se registraron más encuestas completas que las entregadas finalmente en la base de datos, tal como se observa en la tabla que se presenta a continuación, donde tenemos 161 encuestas no válidas, no obstante, se logró un 107% de la muestra objetivo.

Tabla 6: Resultados de los intentos de contactos

Códigos	Número de encuestas
110 = Entrevista Completa	857
111 = Entrevista Terminada no valida	161
120 = Entrevista Parcial	52
210 = No lograda, se negó el entrevistado	8
220 = No lograda, se negó el entrevistado rotundamente	1
230 = No lograda, se negó un familiar del entrevistado	1
233 = Problemas de idioma	1
300 = No lograda, por otras razones	35
510 = Sin CDF	7
**Total**	**1123**

6.3. Productividad

Como se mencionó previamente, durante el trabajo de campo el equipo de encuestadores estuvo conformado por 8 encuestadores, abordando 4 puntos diarios, es decir, no todos los encuestadores estuvieron activos durante todo el período. Solo dos encuestadoras se mantuvieron durante todo el período logrando el mayor número de encuestas realizadas en proporción al total de encuestas válidas. Quienes presentan productividades menores a 10% son encuestadores que realizaron encuestas en las últimas dos semanas del trabajo de campo.

Tabla 7: Productividad de los encuestadores

Encuestadores	Encuestas Sincronizadas	Terminadas	Encuestas validas	%Productividad encuestas sincronizadas	%Productividad muestra valida
**1**	176	146	127	16%	14,9%
**2**	10	9	8	1%	0,9%
**3**	192	175	153	17%	17,8%
**4**	141	131	114	13%	13,3%
**5**	55	44	38	5%	4,5%
**6**	41	39	34	4%	4,0%
**7**	386	341	297	34%	34,7%
**8**	122	98	85	11%	10,0%
**Total**	**1.123**	**983**	**857**	**100%**	**100,0%**

7. Supervisión del Trabajo de Campo

Para la supervisión de la encuesta se usaron dos métodos de control de terreno:

1. Primer Método de Supervisión

El primer método consistió en una visita a los distintos puntos donde se debía posicionar el encuestador con el fin de detectar los espacios donde se ubicaban las personas que se buscaban y que en ese momento se encontraran fumando.

A cada uno de los encuestadores que participaron en este levantamiento se les visitó al menos una vez en el punto inicial asignado y se comprobó visualmente que estuviesen cumpliendo con las instrucciones de:

- Respetar el área asignada para el levantamiento de información, ya que en esta visita se le especificó y delimito el área que podían recorrer para realizar la entrevista.
- Que efectivamente realizaran encuestas a personas que se encontraban fumando y con la cajetilla de la marca correspondiente en sus manos, ya que a veces se encontraban fumando, pero la cajetilla la habían dejado en su oficina o lugar de trabajo, por tanto no eran susceptibles de encuestar.

Posteriormente, en aquellos casos en que el rendimiento del encuestador no era óptimo se efectuaron visitas aleatorias a estos puntos y se comprobó que, aunque estaban en las áreas de encuestaje correspondientes, el flujo estimado de fumadores no era alto, lo que justificaba la demora en el levantamiento de algunos sectores.

2. **Segundo Método de Supervisión**

El segundo método consistió en realizar el control de las encuestas levantadas. Dicha estrategia se efectuó haciendo llamados telefónicos a los números de contacto solicitados por el encuestador a los entrevistados que efectivamente accedieron a entregarlos.

En efecto, se seleccionaron aleatoriamente el equivalente a un 10% de las encuestas realizadas por cada encuestador, esto correspondió a 85 encuestas distribuidas en todos los puntos del levantamiento correspondientes a la muestra objetivo. Hay que considerar que no todos los entrevistados facilitaron su número de celular ya que algunos se negaron por ser privado y otros debido a que no contaban con celular, por tanto, esta muestra de controles se obtuvo de un total de 500 encuestas.

El control cumplió con todos los estándares de calidad y no hubo encuestas objetables, es decir, no se encontraron encuestas que no estuviesen acordes con los objetivos del estudio o con la calidad de los datos recopilados. Las únicas encuestas consideradas como no validas fueron aquellas que no contaban con la fotografía de la cajetilla, requisito que ya se especificó anteriormente.

8. Reporte Cualitativo del trabajo de campo

Como parte del proceso de recolección de información, se contempló una reunión con alguno de los encuestadores participantes en el trabajo de campo con el fin de rescatar información que no es posible observar directamente de la base de datos.

La reunión se realizó el día viernes 21 de julio a las 10:00 am en las dependencias del Centro de Microdatos. A continuación, se detallan cada uno de los temas abordados en el grupo de conversación y los principales hallazgos del trabajo de campo.

- **Presentación**

En una primera instancia se generó la presentación del grupo de personas integrantes en la reunión de finalización del trabajo de campo de la encuesta, luego se abordaron algunos tópicos asociados a la recolección de datos y percepciones acerca del cuestionario.

- **Jornada de Trabajo y Contacto con los encuestados**

En términos generales, se comentó que las jornadas de trabajo fueron de aproximadamente 8 horas, con bastante tiempo muerto por la espera en completar las cuotas y que se concretará el flujo de personas necesario.
Se utilizó el radio demarcado en los planos de la muestra para encontrar flujos de público, como por ejemplo en el punto de Vicuña Mackenna y Victoria, donde al momento de la encuesta se estaba construyendo un nuevo corredor del transantiago, hecho que generó que el punto de afluencia se moviera a otra parte, este inconveniente se identificó y modificó de acuerdo al análisis del jefe de terreno.
La identificación de los horarios asociados a los flujos de las personas fue clave para la optimización del trabajo de campo. Las cuotas de mujeres fueron más complicadas de completar, lo cual podría explicarse porque, posiblemente, ellas fumen menos en la calle, haciendo que fuese más difícil identificarlas y contactarlas.
Los jóvenes en general no contaban con cajetillas, pues obtenían sus cigarrillos a través de terceros.
En el sector socioeconómico más alto costó completar las cuotas de edades, en particular porque los adultos mayores presentaron mayor resistencia y desconfianza para participar de la encuesta.
Las personas que trabajan en supermercados o centros comerciales en general bajan a fumar sin cajetilla sino que solo con el cigarrillo que fumaran.

En el sector socioeconómico más bajo, costo encontrar personas con cajetillas, pues en general tenían cigarrillos sueltos.
Por las dificultades mencionadas previamente, respecto de las cajetillas y contacto, es que los encuestadores optaron por aplicar los filtros previos a comenzar la encuesta, lo que fue autorizado por el coordinador de terreno, ya que la encuesta solamente era válida en la medida que se contara con la autorización del entrevistado para fotografiar su cajetilla.

- **¿Cómo abordaban a los entrevistados?**

Principalmente se identificaba lugar y luego se dirigían donde había gente que se juntaba y estaba fumando, todo dentro del radio especificado en los planos entregados a los encuestadores.

Al principio del levantamiento se comenzaba la encuesta con todos los fumadores, luego al detectar que las personas no tenían cajetillas o no permitían tomar la foto (por temas de seguridad no querían sacar cajetillas de carteras, mochilas o bolsos) se aplicaron filtros de residencia y de tenencia de cajetillas, previo a comenzar la encuesta.

Al ser consultados, los fumadores en general no mostraron rechazo, en particular por ser una encuesta corta que no indagaba demasiado en antecedentes personales, lo que generó confianza a las personas para responder la encuesta honestamente.

Dado los problemas generales de seguridad, las personas mayores de 60 años fueron quienes generaron algún grado de rechazo para responder la encuesta, pero no por el tema a tratar en la entrevista sino más bien por temor a ser asaltado. En cuanto a los jóvenes, si bien no mostraban rechazo a responder la encuesta, fue complejo detectar a aquellos que tuvieran cajetillas, ya que la mayoría solo tenía el cigarrillo que estaba fumando o alguno más, pero no portaban cajetillas.

Los pocos rechazos que se generaron en el resto de la población tuvieron que ver con la mención de la American Cancer Society, puesto que las personas se resistían a escuchar alguna recomendación sobre su salud y el hábito de fumar, en algunos casos ni siquiera dando la oportunidad al encuestador de explicar que no era el objetivo de la encuesta.

Un aspecto relevado por los encuestadores fue en los puntos donde se encontraban las construcciones o plazas en segmentos de bajos ingresos, las personas en lugar se fumar cigarrillos fumaban marihuana, lo que dificultaba el contacto en estos segmentos de la población.

- **Dificultades para el levantamiento**

Entre las dificultades mencionadas que no tuvieron base en las características de la encuesta, fueron el mal clima, lluvias, bajas temperaturas, protestas ciudadanas que no permitieron completar las encuestas en una sola jornada de trabajo como estaba previsto.

Se sugirió ampliar el universo a quienes fuman tabaco y no solamente cigarrillos. También se recomendó ampliar para aquellos que no estén fumando pero que pueden ser potenciales fumadores.

Las encuestas se realizaron previo a las 18:00 horas, pues posterior a tal hora la luz natural es escasa y es muy difícil distinguir la información de las cajetillas e incluso en algunos sectores se tornaba peligroso.

Cabe destacar que se generó una dificultad con el manejo de la Tablet al tratar de tomar la foto de la cajetilla, ya que esta se debía apoyar en alguna parte y la Tablet tiene un tamaño que hace complejo su manejo. Para solucionar dicho problema los encuestadores tuvieron que pedir ayuda para sostener la cajetilla al tomar la foto, especialmente para enfocar correctamente. Adicionalmente, se requirió también de atención especial para evitar posibles robos. Se sugirió que la Tablet facilite el

control de cuotas e indique que las encuestas faltantes como guía evitar ver la hoja de ruta, también incorporar el cálculo de la edad que se hizo complejo.

En términos de calidad de los datos se observó en el trabajo de campo que las mujeres en general subreportan su edad y los hombres la redondean hacia arriba.

Los filtros del cuestionario debiesen haber estado al principio de la encuesta, por ejemplo, en lo relacionado a si tenían o no cajetilla y si permitiría o no tomar una foto. Se generaron muchos tiempos muertos por el cumplimiento de cuotas y de horarios de flujo de personas. En promedio se realizaron 2 visitas a cada punto para lograr las encuestas.

Se detectó la venta de cigarrillo suelto, ya que al abordar al entrevistado y aplicar los filtros asociados a la cajetilla, los encuestados revelaban que los habían comprado sueltos, en particular en los estratos socioeconómicos bajos. Los encuestadores sugirieron que de acuerdo a su observación 9 de cada 10 jóvenes no compraban cajetillas, sino cigarrillos sueltos.

En Estación Central, principalmente por ser una comuna con importante tránsito de personas (terminales de buses y trenes), se perdía muestra porque se encontraba mucha gente de otras comunas de la RM que son rurales o de personas que no viven en esta región, esto también pasó en las afueras de los hospitales cuando se abordó a los posibles entrevistados. Finalmente, se aplicó el filtro de residencia antes de hacer la encuesta.

En los colegios católicos, en general no se encontraban jóvenes con las características de la muestra.

Los tiempos de desplazamiento y el alto número de visitas a algunos puntos de recolección de datos generaban que el proyecto no estuviese bien pagado de acuerdo con la percepción de los encuestadores.

- **Otros aspectos y sugerencias**

La productividad de las entrevistas alcanzaron hasta 10 encuestas por punto y en promedio eran requeridas 20, por lo que en un día no fue posible completar las cuotas en cada punto.

Dado que la instrucción era entrevistar personas que estuviesen fumando, los compradores de cigarrillos no fueron objeto de posible contacto, por tanto, se sugiere incorporarlos como posibles seleccionados en una nueva versión de la encuesta.

Otro tema es la detección de compra y venta de cigarrillos sueltos, sería importante tomar en cuenta este punto y generar un filtro que permitiera seguir con la encuesta y cuantificar dicha práctica.

En cuanto a la programación del cuestionario, se sugiere incorporar en el diseño del cuestionario una tabla que administre las cuotas, determinando automáticamente la edad del entrevistado, lo que reduciría el posible error asociado al cálculo realizado por el encuestador.

La falsificación de cigarrillos es muy difícil de detectar en el levantamiento, en general se detecta por las advertencias, pero para ello se requiere un conocimiento importante de ellas y de los años de publicación.

En cuanto al cuestionario, los encuestadores advirtieron que las preguntas de la marca y el país parecían repetitivas, pues se contaba con la foto, fuente desde la que se podían obtener esta información.

9. Depuración de Base de Trabajo de Campo

Una vez digitalizada la información proveniente del trabajo de campo, fue necesario corroborar que la información de cada encuestado correspondiera a la información registrada por la fotografía que se le tomó a la cajetilla del encuestado. Se

corrigieron todas las encuestas cuyo registro no coincidiera con la información de la cajetilla, así como también se eliminaron de la base de datos las encuestas con fotos inutilizables[7].

Para identificar errores en la encuesta fue necesario revisar las fotografías de las cajetillas de cigarrillos una a una, registrando, de forma separada de la encuesta, las características de cada cajetilla (Marca, cantidad, advertencia[8] y si correspondía a cigarrillo mentolado). Una vez identificadas estas características, se procedió a comparar la información de las fotografías con la registrada en la encuesta.

El primer paso de la depuración fue eliminar las encuestas con errores imposibles de corregir mediante las fotografías. Se eliminaron de la base 20 encuestas con fotos ilegibles (muy oscuras, desenfocadas y que no correspondían a cajetillas), 25 encuestas de personas que no contestaron la pregunta correspondiente a la edad de inicio (o la edad de inicio era posterior a la edad actual reportada) y 2 encuestas en las que las personas declararon comprar cigarrillos sueltos. En total, 47 encuestas fueron descartadas de la base de datos, las que equivalen a un 5,48% de la base total original; la base final se compuso de 810 encuestas, aún por sobre las 800 encuestas de la muestra teórica inicial.

Posteriormente, se identificaron 96 encuestas donde la marca registrada no correspondía con la marca de la fotografía; todos aquellos casos fueron corregidos por la marca en la fotografía. De igual forma, se identificaron 38 encuestas cuya cantidad de cigarrillos en la cajetilla se encontraban mal identificados, nuevamente se corrigió en base a la cantidad de la fotografía.

Finalmente, para identificar el precio que se pagó por cajetilla, se les preguntó a los encuestados el precio y la marca de la última cajetilla comprada, suponiendo que esta sería la misma que se encontraban fumando al momento de la encuesta (y la

[7] Se consideran como inutilizables aquellas fotos muy oscuras, extremadamente desenfocadas o que no correspondían a cajetillas de cigarrillos.
[8] Advertencia de Chile, otro país o sin advertencia.

misma de la foto). Sin embargo, se identificaron 20 encuestas en las que la marca declarada de la última compra difería de la marca consumida al momento de la encuesta, además de 12 encuestas en las que los encuestados no respondieron a la pregunta de precio. A estos casos se les imputó la mediana del precio de las cajetillas del mismo precio y misma cantidad.

Una vez corregidas todos los errores en la base entregada, se procedió a crear variables relevantes para caracterizar el consumo de cigarrillos. **La primera que fue necesario crear fue el origen de la los cigarrillos consumidos (legales o contrabandeados). Estos fueron definidos como toda cajetilla que no contara con alguna advertencia para Chile y que no hubiesen sido adquiridos de forma legal**. Los cigarrillos de procedencia extranjera fueron considerados como compras legales sólo si es que estos fueron adquiridos en zonas francas, fuera del país o por medio de algún conocido. Además, se crearon variables respecto al nivel educacional, situación laboral, edad e intensidad de consumo (cigarrillos consumidos por día).

9.1. Ajuste por Factor de Expansión

Con el fin de que el presente trabajo sea representativo con la población de fumadores en la Encuesta Nacional de Población General 2014 (ENPG 2014) realizada por el Servicio Nacional para la Prevención y Rehabilitación del Consumo de Drogas y Alcohol (SENDA), fue necesario ajustar la base por un factor de expansión. Este fue creado en base a las estratificaciones por grupos de edad y sexo utilizados en el marco muestral[9] (en total 8 estratos) y a las proporciones de estos en la ENPG 2014 de forma que el factor de expansión fue igual a:

[9] Cuatro grupos de edades (13-17, 18-34, 35-65 y 65 o más años) y ambos sexos.

$$FE_{ij} = \frac{PoblaciónENPG_{ij}}{PoblaciónCont_{ij}}$$

Donde el factor de expansión (FE) de un individuo de sexo i y grupo de edad j es igual a la población total en la ENPG 2014 con sexo i y grupo de edad j (ajustada por factor de expansión), dividido por la población total de individuos de seco i y grupo de edad j en la encuesta del presente estudio.

Para hacer el análisis de las siguientes secciones comparable con la población total de fumadores, las estadísticas se presentan todas ajustadas por el factor de expansión construido.

10. Principales Resultados

10.1. Caracterización Demográfica de la Muestra

Como se observó en la sección de resultados del trabajo de campo (sección 4) y en su posterior depuración, la base final contó con 810 entrevistas, 1,25% más de lo requerido. Las cuotas definidas establecían una distribución de acuerdo con los datos de las últimas encuestas de población general de senda. Al analizar la distribución de género de los entrevistados (ajustados por factor de expansión) en la Tabla 8 se puede apreciar un balance equilibrado entre hombres y mujeres, con un 49,98% del total de hombres y un 50,02% mujeres.

Tabla 8: Distribución de Género Ajustados por Factor de Expansión

Género	Frecuencia	%
Hombres	404,81	49,98
Mujeres	405,19	50,02
**Total**	**810**	**100**

En cuanto a la edad de los entrevistados, éstas van desde un mínimo de 13 años a un máximo de 88 años. Luego, según tramo etario (Figura 3), se observa que la mayor parte de las personas se concentran entre los 18 y los 30 años (26,33%), entre los 31 y los 40 años (22,63%) y entre los 41 y los 50 años (24,89%). Además, un 3,22% de las personas son menores de edad y un 5,29% son mayores de 60 años.

Figura 3 – Distribución de la muestra según tramos de edad

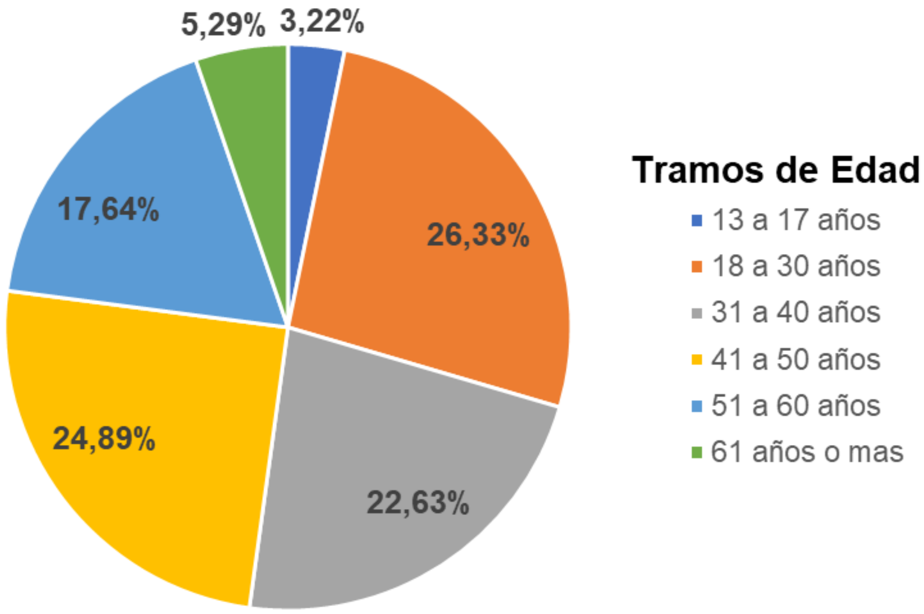

Con respecto a los niveles educacionales de los encuestados (Figura 4), un 5,66% afirma haber alcanzado como máximo el segundo ciclo de la enseñanza básica (hasta 8°), mientras que un 42,94% dice haber obtenido enseñanza media. Además, un 15,76% tiene un título técnico y un 17,87% título universitario, mientras quienes tienen estudios de postgrado (con o sin título) representan apenas un 0,65% de la muestra.

Figura 4 – Distribución de la muestra según máximo nivel educacional alcanzado

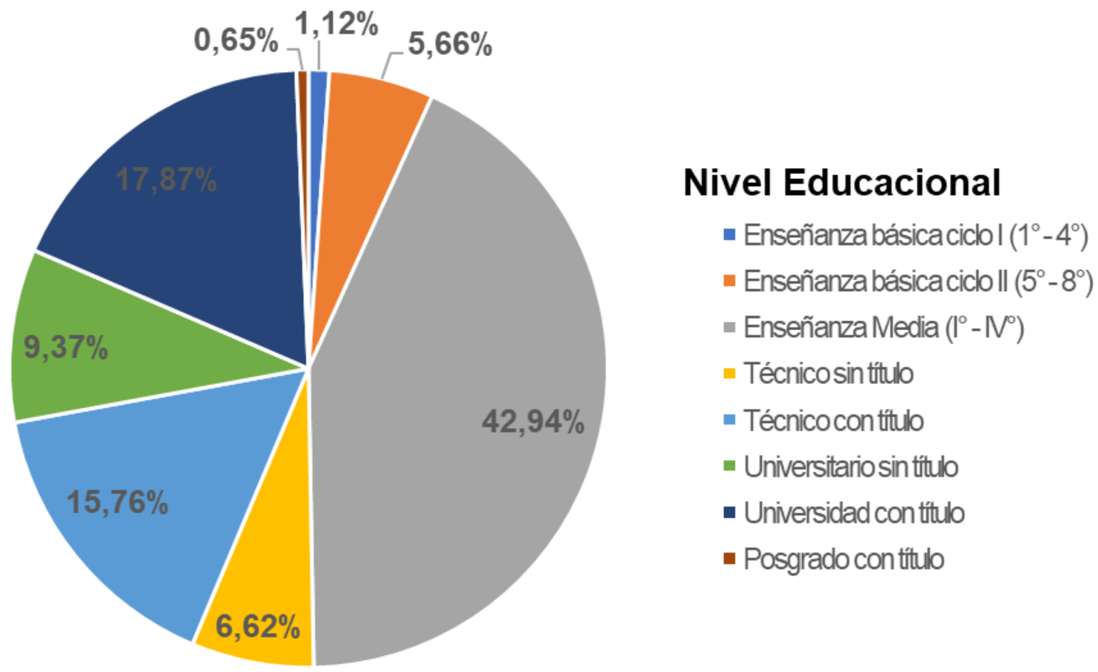

En relación con las actividades realizadas (Tabla 9) por los entrevistados, la mayoría dedicó en la semana previa a la entrevista la mayor parte de su tiempo a trabajar (76,41%), mientras que otro grupo importante lo dedicó al estudio (8,43%). De aquellos que declararon trabajar, un 74,64% expresó ser asalariado (empleados u obreros) y 25,36% dijo ser independientes o trabajador por cuenta propia. Más pequeños son los grupos de personas que se encontraban en la búsqueda de trabajo (6,53%), oficios del hogar (5,6%) o que eran pensionados (2,22%). Aquellos que afirmaron realizar otra actividad especifican que “no hacen nada” representan un 0,27% del total de encuestados.

Tabla 9 – Actividad en la que ocupó la mayor parte del tiempo la semana pasada

Actividad	Frec	%
Trabajando	618,93	76,41
Buscando trabajo	52,93	6,53
Pensionado	17,97	2,22
Estudiando	68,30	8,43
Oficios del hogar	45,40	5,60
Incapacitado permanente para trabajar	4,26	0,53
Otra Actividad. Especifique	2,22	0,27
**Total**	810	100

Finalmente, al analizar la distribución de la comuna de residencia de los encuestados, se observa que ellos se distribuyen con diferentes niveles de concentración en 42 de las 52 comunas pertenecientes a la Región Metropolitana. Las comunas donde vive la mayor parte de los encuestados son Puente Alto (10,70%), Santiago (8,48%), La Florida (5,82%), Maipú (4,62%) y Pudahuel (4,58%). En el lado contrario, las comunas de Peñaflor, Colina, Padre Hurtado, Talagante, Buin, El Monte y Paine son en las que viven menos personas del grupo de encuestados, con menos del 0,5% del total en cada una de ellas. Siendo todos residentes de la Región Metropolitana.

10.2. Resultados de Preguntas Sobre Hábitos de Consumo de Cigarrillos

A continuación, se indaga en las preguntas del cuestionario relacionadas al consumo de cigarrillos. En primer lugar, en la Tabla 10, se observa que un 94,7% de las personas consume cigarrillos diariamente, mientras que un 5,15% lo hace

algunos días a la semana y sólo un 0,15% de los fumadores fuman menos de una vez a la semana.

Tabla 10 – Frecuencia e Intensidad con que fuman cigarrillo

**Frecuencia**	Diariamente	Algunos días a la semana	Menos de una vez a la semana	**Total**
**%**	94,70	5,15	0,15	100
**Intensidad**	9,67	8,61	6	9,53

Las personas que expresaron consumir cigarrillos diariamente fuman, en promedio, 9,67 cigarrillos al día y, al desagregar según género, se encuentra que los hombres fuman al día, en promedio, más cigarrillos que las mujeres (10,24 vs 8,97). Por otro lado, al descomponer según la actividad que realizan estas personas, se encuentra que quienes más fuman al día, en promedio, son los pensionados (11,94 cigarrillos), los incapacitados permanentemente (10,27 cigarrillos), los que trabajan (10,91 cigarrillos) y los que buscan trabajo (9,81 cigarrillos), mientras que al desagregar según el nivel educacional alcanzado se encuentra que quienes más fuman al día, en promedio, son aquellos que sólo alcanzaron el primer y el segundo ciclo de la enseñanza básica (13,87 y 11,66 cigarrillos, respectivamente). Por otro lado, las personas que consumen cigarrillos algunos días de la semana fuman, en promedio, 7,77 cigarrillos a la semana.

Al analizar las edades en que las personas probaron por primera vez el cigarrillo se observa que éstas van desde los 6 hasta los 57 años y que, en promedio, esto ocurre a los 16,7 años, sin presentar grandes diferencias entre hombres y mujeres.En la Figura 5 se puede ver que más de las tres cuartas partes de la muestra probó el cigarrillo por primera vez antes de los 15 años (30,40%) o entre los 15 y los 18 años (46,08%). También existe un grupo importante de personas,

aunque más pequeño, que lo probó por primera vez entre los 19 y los 25 años (19,32%).

Figura 5 – Edad en que probaron el cigarrillo por primera vez

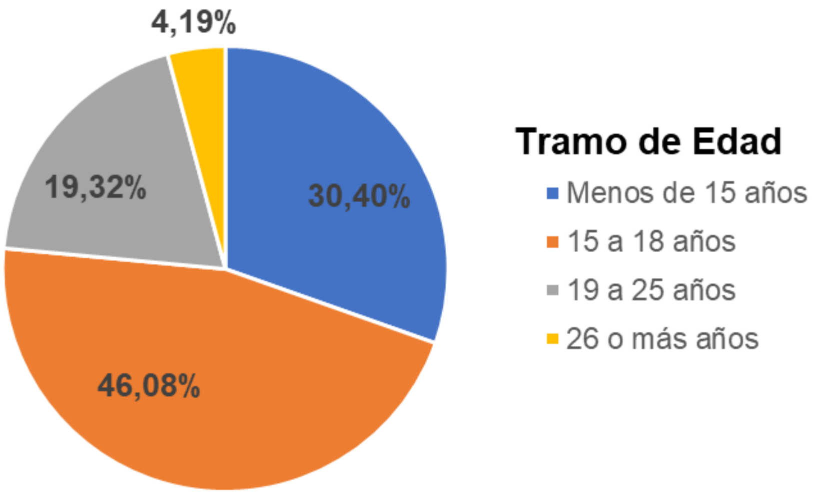

En relación a las marcas de cigarrillo que fumaron las personas la última vez (Figuras 6a y 6b), se observa un claro predominio de ciertas marcas en particular: un 45,44% fumó Pall Mall, un 22,19,3% fumó Lucky Strike y un 18,27% fumó Kent. Al descomponer según género se observa que son las mujeres las que más fumaron Pall Mall (51,8% vs 39,1% de los hombres) y los hombres los que más fumaron Lucky Strike (25,6% vs 18,8% de las mujeres). Mientras que al descomponer por tramo de edad se observa que los dos grupos más jóvenes consumen más Pall Mall (51,88% y 48,29% respectivamente) y los dos grupos de mayores edades consumen más Kent.

En un nivel más bajo de consumo destacan marcas como Fox (2,4%), Jaisalmer (2,1%) y Carnival (2%), mientras que menos de un 0,5% consumió la última vez marcas como Nirvana, Business Class, Latino, Parliament, Indy, Viceroy u otros.

Figura 6a – Marca de cigarrillos que fumaron la última vez por sexo

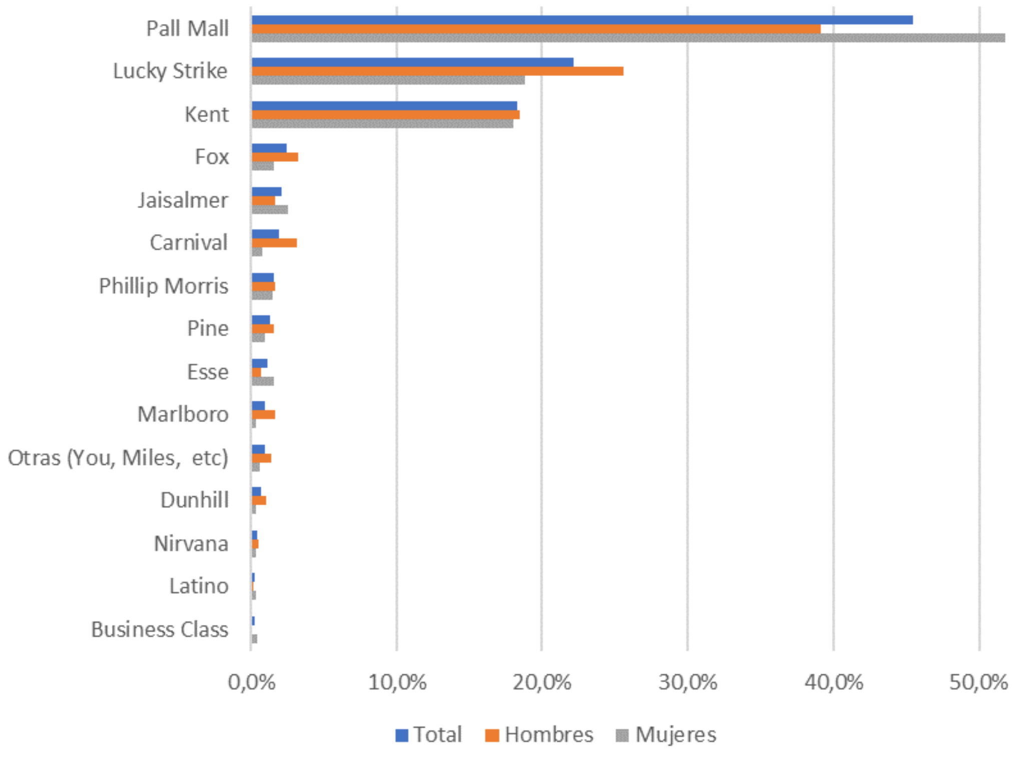

Figura 6b– Marca de cigarrillos que fumaron la última vez por edad

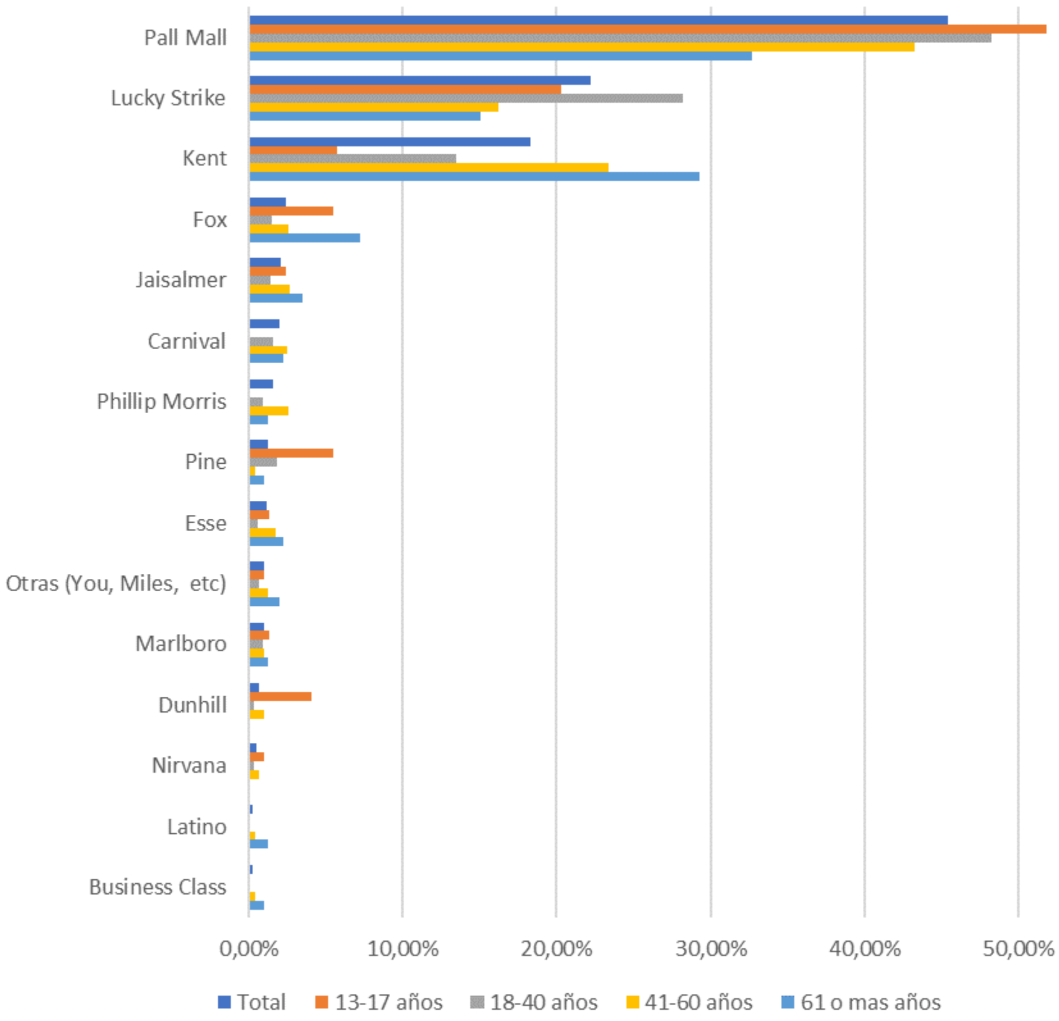

Con respecto a la procedencia y sabor de los cigarrillos consumidos, se puede observar en las Figuras 7 y 8 respectivamente que la prevalencia del contrabando alcanza un 10,87% de los cigarrillos consumidos, donde se consideran cigarrillos de contrabando como todos aquellos que, en las fotografías tomadas, no presenten alguna advertencia para Chile y que no hayan sido adquiridas de forma legal. Por el lado de los cigarrillos saborizados, estos alcanzan un 39,88% del total. Estos temas serán abordados en mayor profundidad en la sección 8.3.

Figura 7– Procedencia de cigarrillos consumidos

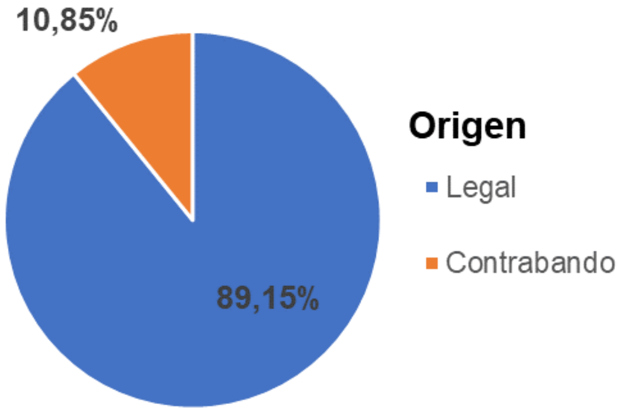

Figura 8 – Sabor de cigarrillos consumidos

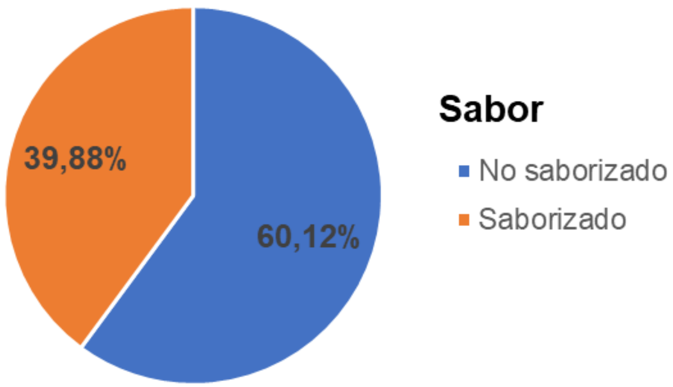

Al analizar los lugares donde las personas compraron cigarrillos la última vez en la Figura 8a, se observa que la gran mayoría los compró en una tienda de barrio (41,48%) o en una tabaquería/botillería (18,73%), mientras que una proporción menor en un supermercado (9,78%), a vendedores ambulantes (9,29%) o en una

estación de servicio (7,56%). Cabe destacar que un 9,65% personas que señalan que los compraron en otro lugar, entre las que destacan kioskos, regalado y en tiendas de metro.

Si observamos la distribución del lugar de última compra de quienes adquirieron cigarrillos legales (Figura 8b), se puede apreciar que ésta sigue una distribución similar al lugar de última compra de todos los cigarrillos (Figura 8a), excepto en las compras a vendedores ambulantes, la que disminuye de 9,29% a 0,97%. La razón de esta disminución es que el lugar de última compra de quienes adquirieron cigarrillos de contrabando (Figura 8c) fue, en gran parte, a vendedores ambulantes; alcanzando un 77,5% de los lugares de compra de cigarrillos de contrabando. En una menor proporción, pero aún importante, los consumidores de marcas de procedencia ilegal realizaron su última compra en tiendas de barrio (17,44%) y el 5% restante lo realizó en tabaquerías o especificó otro (en la calle).

Figura 8a – Lugar de última compra de cigarrillos totales

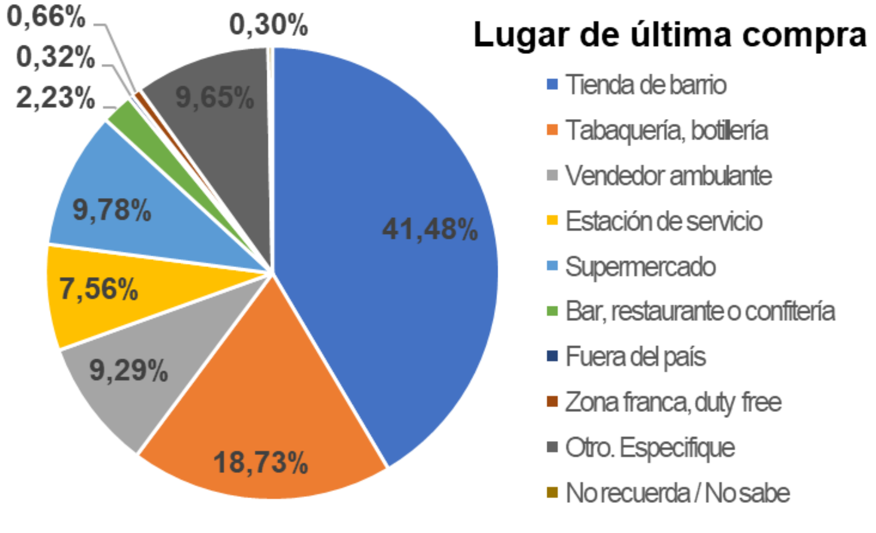

Figura 8b – Lugar de última compra de cigarrillos legales

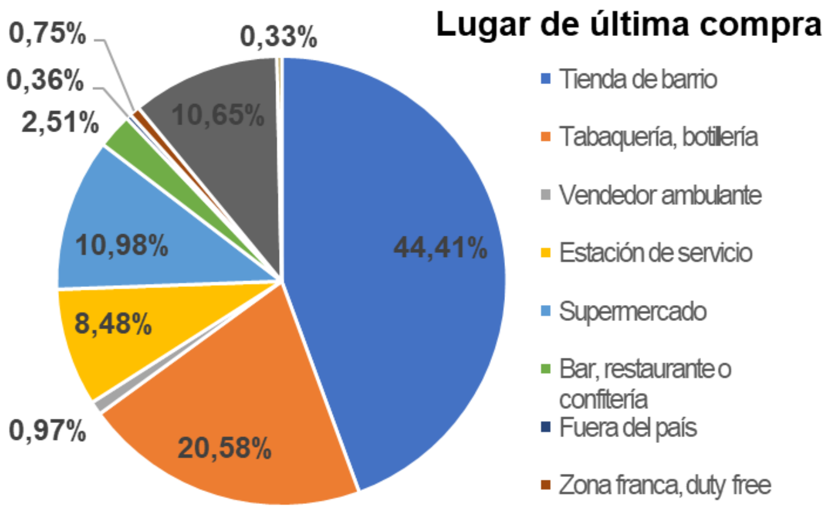

Figura 8b – Lugar de última compra de cigarrillos legales

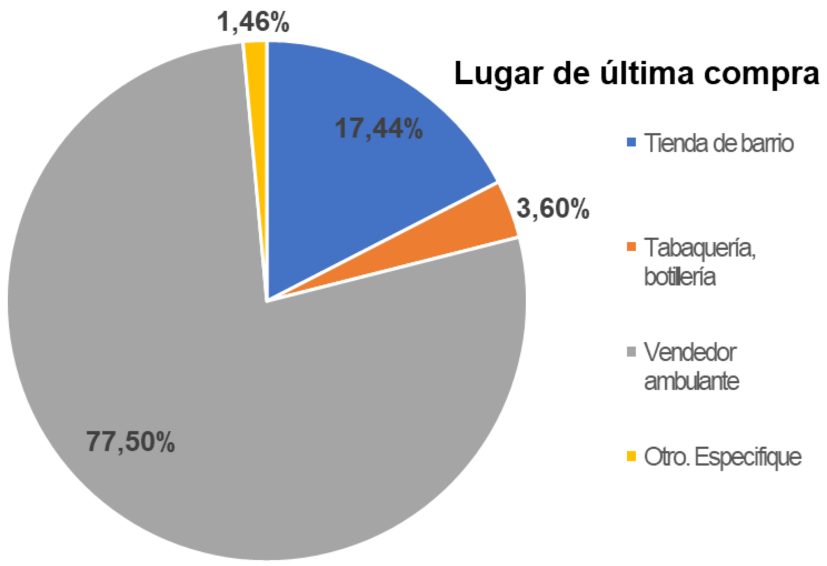

En cuanto a esta última compra, sólo 20 encuestados de los 810 declaró que la marca de esa última compra era distinta a la marca que se encontraban fumando al momento de la encuesta (última marca consumida). Con respecto al tipo de compra realizada esta última vez, casi la totalidad de las personas (99,18%) reportan haber comprado una cajetilla de cigarrillos, mientras sólo un 0,82% reportó haber comprado un cartón. De quienes compraron una cajetilla de cigarrillos, la mayoría compró una cajetilla de 20 cigarrillos (72,46%), un 26,99% compró cajetillas de 10 y sólo un 0,55% compró cajetillas de 11.

Los precios de las cajetillas compradas (Figura 10) van desde los $990 pesos hasta los $4.400, con un valor promedio de $2.497. Al descomponer esta información por tramos de precios se puede observar que la distribución de los precios pagados por las personas (que de alguna manera reflejan su disposición a pagar) es relativamente pareja entre los tramos que van desde los $1.000 a los $3.999: un 33,51% del total de la muestra compró la cajetilla por un valor entre $1.000 y $1.999, un 35,43% lo hizo por un valor entre $2.000 y $2.999 y un 30,65% lo hizo por un valor entre $3.000 y $3.999. Por otro lado, son muy pocas las personas (0,4%) que compraron la cajetilla de cigarrillos por valores iguales o superiores a $4.000.

Figura 10 – Precio pagado por la última cajetilla

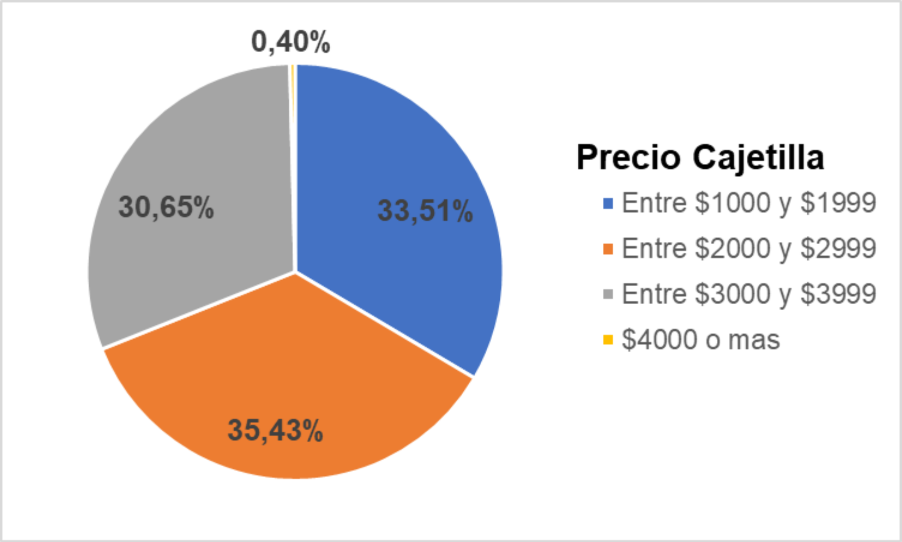

Los precios unitarios por cigarrillos presentan una gran variación según su origen. En la Figura 11 se puede apreciar que los precios unitarios de los cigarrillos legales poseen una distribución distinta a la de los de contrabando; la mediana de los primeros es $155 pesos, versus los $50 pesos de los de contrabando. Además, los cigarrillos legales poseen una variación mayor a los de contrabando, tal que, para las marcas legales, el límite inferior del gráfico de cajas es de $107,5 pesos y el límite superior alcanza los $400 pesos (versus los $49,5 y 160 pesos, respectivamente, de las marcas de contrabando). Sobre los precios mínimos y máximos reportados, se reportó que, para las marcas legales, los precios unitarios van desde $95 pesos a $400 pesos, mientras que para las marcas de origen ilícito el rango va desde $49,5 pesos a $160 pesos.

Figura 11 – Distribución de Precio Unitario por Origen

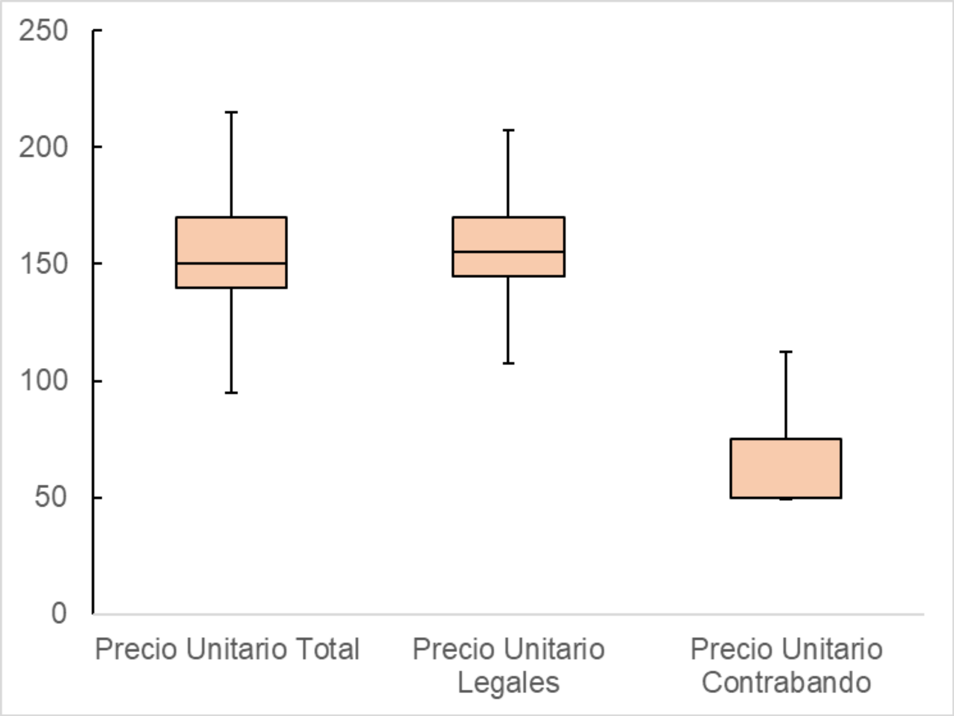

10.3. Caracterización de Fumadores

Dado que el principal objetivo del presente estudio es estimar el contrabando de cigarrillos en el Gran Santiago, resulta crucial caracterizar el consumo de cigarrillos por diferentes grupos de interés. En la siguiente sección se analizan las características de los fumadores desagregando entre quienes consumen marcas de contrabando y marcas legales. Adicionalmente, se caracteriza el consumo de cigarrillos desagregado por sexo, grupos de edad, nivel educativo, situación laboral y tiempo como fumador.

En la Tabla 13 se presentan la media y desviación estándar de las principales variables demográficas y de hábitos tabáquicos, desagregadas por el origen de los cigarrillos consumidos. Respecto al sexo, el porcentaje de mujeres es menor entre los fumadores de contrabando (40,9% versus 51,14% en los fumadores de marcas legales). En promedio, quienes fuman cigarrillos de contrabando tienen una edad mayor a quienes consumen marcas legales; la edad promedio de los fumadores de cigarrillos legales es 39,36, en contraste con los 42,72 de fumadores de contrabando.

Si se analiza por nivel educativo, se aprecia que el máximo nivel educativo alcanzado es mayor entre quienes compran marcas legales; más de un 50% tiene algún estudio universitario o técnico, un 35% posee a educación media completa y cerca del 12% no completó la educación primaria o la educación media. Por el lado de quienes compran marcas de contrabando, sólo un 24,62% tiene algún estudio universitario, menos de la mitad de quienes compran marcas legales, y más de un 35% no terminó la educación primaria ni la media o no tiene estudios. Además, entre quienes consumen marcas legales, el 78,42% de ellos está empleado, versus el 58,59% de quienes consumen marcas de contrabando.

Sobre las características del consumo, el precio promedio pagado por cigarrillo legal es más del doble que los cigarrillos de contrabando, siendo estos $160 y $60 pesos respectivamente. La cantidad de cigarrillos diarios consumidos también varía según el origen de estos; los fumadores de cigarrillos legales consumen, en promedio, 8,74 cigarrillos diarios, en contraste con los 13,03 cigarrillos diarios de los fumadores de cigarrillos de contrabando. Desagregados por tramos de consumo diario, el porcentaje de fumadores de marcas legales que consume diariamente entre 1 y 5 cigarrillos, 6 y 10 cigarrillos, 11 y 20 cigarrillos y más de 20, es de 40,63%, 36,98%, 20,48% y 1,91% respectivamente. Mientras que por el lado de los fumadores de marcas de contrabando sigue una distribución opuesta, donde quienes consumen diariamente entre 1 y 5 cigarrillos, 6 y 10 cigarrillos, 11 y 20 cigarrillos y más de 20, es de 16,66%, 36,24%, 44,16% y 2,94% respectivamente. Además, un 41,55% de los fumadores de marcas legales compra cigarrillos saborizados, casi el doble que los fumadores de marcas de contrabando (24,8%).

Tabla 12 – Características de Fumadores por Origen de Cigarrillos

	Legal		Contrabando		Total	
	Media	Desv. Estándar	Media	Desv. Estándar	Media	Desv. Estándar
**% Mujeres**	51,14%	0,50	40,90%	0,49	50,03%	0,50
**Edad**	39,36	13,02	42,72	15,14	39,72	13,30
**Sin educación o primaria incompleta**	1,92%	0,14	7,01%	0,26	2,47%	0,16
**Primaria Completa, Media Incompleta**	9,68%	0,30	29,88%	0,46	11,87%	0,32
**Media Completa**	35,01%	0,48	38,48%	0,49	35,39%	0,48
**Alguno Universitario/Técnico**	53,39%	0,50	24,62%	0,43	50,27%	0,50
**% Empleados**	78,42%	0,41	58,59%	0,50	76,27%	0,43
**Precio por cigarrillos**	160,11	28,21	61,19	19,50	149,38	41,20
**% Cigarrillos Saborizados**	41,55%	0,49	24,80%	0,43	39,73%	0,49
**Cigarrillos por Día**	8,74	6,64	13,04	7,55	9,21	6,87
**% fuma entre 1-5 cigarrillos por día**	40,63%	0,49	16,66%	0,37	38,03%	0,49
**% fuma entre 6-10 cigarrillos por día**	36,98%	0,48	36,24%	0,48	36,90%	0,48
**% fuma entre 11-20 cigarrillos por día**	20,48%	0,40	44,16%	0,50	23,05%	0,42
**% fuma más de 20 cigarrillos por día**	1,91%	0,14	2,94%	0,17	2,02%	0,14

Desagregando por sexo (Tabla 13 y Figura 12) se aprecia que entre los hombres hay un mayor porcentaje de fumadores que compran marcas de contrabando (12,9% versus 8,9% en mujeres). Sobre la intensidad de consumo, los hombres consumen, en promedio, 9,8 cigarrillos diarios, mayor a los 8,7 cigarrillos diarios que consumen, en promedio, las mujeres. El mayor consumo medio de cigarrillos diarios por parte de los hombres también se puede apreciar en el porcentaje de individuos que consumen más de 10 cigarrillos diarios, donde, en los hombres, ese porcentaje llega al 28,6%, mientras que en las mujeres representa un 21,7%. También destaca que un mayor porcentaje de mujeres consume cigarrillos saborizados que los hombres (46,8% versus 32,6% en hombres). No se observan diferencias entre el precio por cigarrillo pagado, por sexo.

Por grupos de edad (Tabla 14 y Figura 13), se aprecia que el grupo etario que presenta un mayor porcentaje de consumo de marcas de contrabando es el grupo de mayor edad (61 años o más) con un 19,1% del total, seguidos por el grupo de menor edad (16,6% del total). Por otro lado, el grupo etario que presenta un menor consumo de cigarrillos de contrabando es el rango entre 18 y 40 años (8,2%). Respecto al consumo de cigarrillos diarios, se puede ver que, a mayor edad, mayor es el consumo de cigarrillos por día (de 5,7 cigarrillos diarios para el grupo de menor edad a 11,8 para el grupo de mayor edad). También resulta importante destacar que entre los jóvenes existe un mayor porcentaje de fumadores que consumen cigarrillos saborizados; entre los menores de 18 años más de la mitad de los fumadores consume cigarrillos saborizados (56,7%)

Analizando por nivel educativo (Tabla 15 y Figura 14) se observa una tendencia para prácticamente todas las variables de consumo. Las personas con un menor nivel educativo presentan un porcentaje mayor de consumo de marcas de contrabando con un 30,7% del total, porcentaje que disminuye hasta quienes poseen algún estudio universitario (5,3%). Respecto a la cantidad de cigarrillos consumidos diariamente, la situación es similar; las personas de menor nivel educativo consumen en promedio 12,9 cigarrillos al día, y a medida que aumenta el nivel educativo, menor es la cantidad de cigarrillos diarios que se consumen (8,6

cigarrillos diarios para el grupo de nivel educativo más alto). El porcentaje de fumadores que consume entre 1 y 5 cigarrillos a la semana es menor entre los individuos de menos estudios (23,6%) y mayor en los individuos con mayor nivel educativo (43,73%), mientras que los individuos de menores estudios son los que tienen un mayor porcentaje de fumadores que consumen más de 20 cigarrillos al día (2,77%), sin existir grandes diferencias entre los demás grupos. También destaca que, a mayor nivel de estudios, mayor es el precio promedio pagado y mayor es el porcentaje de fumadores que prefiere cigarrillos saborizados.

En Tabla 16 (y figura 15), se presentan las características de consumo desagregadas por situación laboral. Se observa que los fumadores empleados son los que presentan un menor porcentaje de fumadores de marcas de contrabando (8,3%); en los fumadores desempleados ese porcentaje es mas de doble (20,4%), seguidos por los fumadores desocupados (18,37%). No existen diferencias en la cantidad promedio de cigarrillos consumidos diariamente entre los fumadores empleados y desempleados, sin embargo, los individuos desocupados reportan un consumo promedio menor (7,7) y los individuos empleados presentan un mayor porcentaje de personas que fuman más de 20 cigarrillos diarios (2,2% versus los 0,2% de los individuos empleados y 1,8% de los desocupados).

Finalmente, en la Tabla 17 y Figura 16, se desagrega por el tiempo que llevan como fumador. Se puede apreciar que casi el 60% de los fumadores más nuevos (10 años o menos) consumen cigarrillos saborizados, porcentaje que disminuye en aquellos que han sido fumadores por más años. De forma contraria, los fumadores más antiguos son los que presentan un mayor porcentaje de fumadores que prefieren marcas de contrabando (15,2%), mientras que quienes han sido fumadores entre 11 y 22 años son los que reportan el menor porcentaje de fumadores de marcas de contrabando (6,1%). Sobre la intensidad de consumo, entre menos años llevan fumando los encuestados, menor es el consumo diario de cigarrillos reportado; los fumadores más nuevos consumen en promedio 6,8 cigarrillos diarios, aumentando hasta los consumidores más antiguos (11,1 cigarrillos diarios). Además, se observa que los consumidores nuevos (menos de 23 años fumando) tienen una disposición

a pagar mayor, donde el precio promedio pagado por cigarrillo asciende a $156 pesos, mientras que los fumadores más antiguos pagan, en promedio, $138 pesos por cigarrillo.

Tabla 13 – Características del Consumo de Cigarrillos por Sexo

		Hombres			Mujeres			Total	
		Media	Desv. Estándar		Media	Desv. Estándar		Media	Desv. Estándar
% Cigarrillos de Contrabando		12,9%	0,34		8,9%	0,28		10,9%	0,31
% Cigarrillos Saborizados		32,6%	0,47		46,8%	0,50		39,7%	0,49
Cigarrillos diarios promedio		9,8	6,77		8,7	6,94		9,2	6,87
% Fuma 1-5 cigarrillos por día		35,8%	0,48		40,1%	0,49		37,9%	0,49
% Fuma 6-10 cigarrillos por día		35,6%	0,48		38,2%	0,49		36,9%	0,48
% Fuma 11-20 cigarrillos por día		26,4%	0,44		19,8%	0,40		23,1%	0,42
% Fuma más de 20 cigarrillos por día		2,2%	0,15		1,9%	0,14		2,0%	0,14
Precio por cigarrillos		$149	42,17		$149	39,83		$149	40,99

Figura 12 – Características del Consumo de Cigarrillos por Sexo

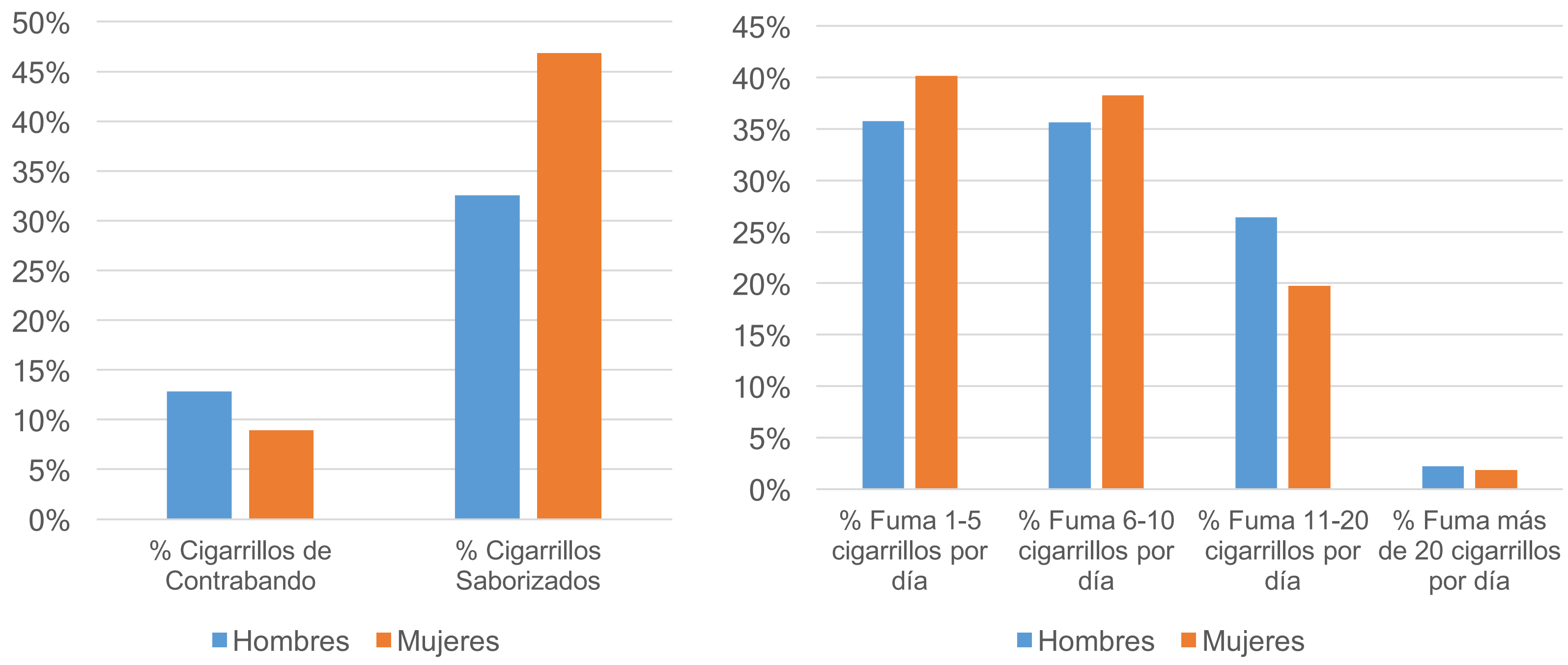

Tabla 14 – Características del Consumo de Cigarrillos por Grupos de Edad

	13 - 17 años		18 - 40 años		41 - 60 años		61 o mas años		Total	
	Media	Desv. Estándar	Media	Desv. Estándar	Media	Desv. Estándar	Media	Desv. Estándar	Media	Desv. Estándar
% Cigarrillos de Contrabando	16,6%	0,37	8,2%	0,27	12,5%	0,33	19,1%	0,40	10,9%	0,31
% Cigarrillos Saborizados	56,7%	0,50	49,4%	0,50	29,5%	0,46	21,9%	0,42	39,7%	0,49
Cigarrillos diarios promedio	5,7	6,06	8,1	5,83	10,4	7,38	11,8	9,01	9,2	6,87
% Fuma 1-5 cigarrillos por día	70,9%	0,46	44,1%	0,50	30,1%	0,46	24,4%	0,43	37,9%	0,49
% Fuma 6-10 cigarrillos por día	19,6%	0,40	37,2%	0,48	37,3%	0,48	41,3%	0,50	36,9%	0,48
% Fuma 11-20 cigarrillos por día	6,7%	0,25	17,1%	0,38	30,5%	0,46	28,8%	0,46	23,1%	0,42
% Fuma más de 20 cigarrillos por día	2,7%	0,16	1,5%	0,12	2,1%	0,14	5,4%	0,23	2,0%	0,14
Precio por cigarrillos	$146	44,35	$156	39,83	$143	39,70	$140	49,49	$149	40,99

Figura 13 – Características del Consumo de Cigarrillos por Edad

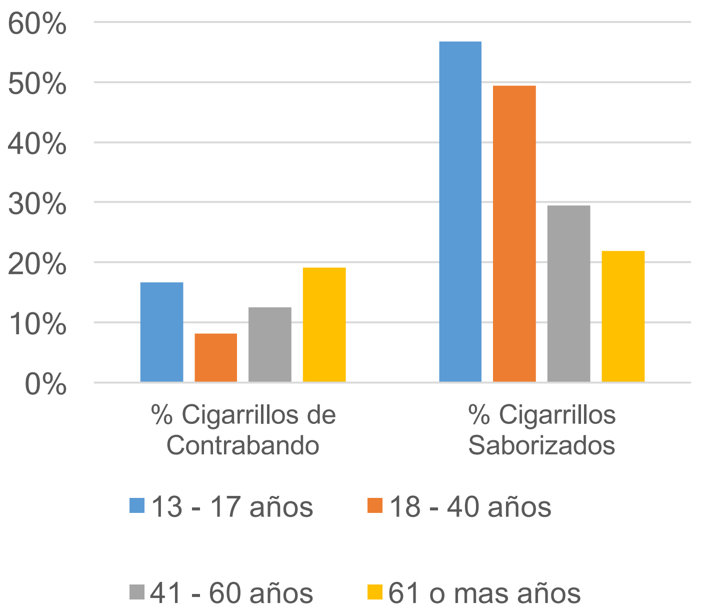

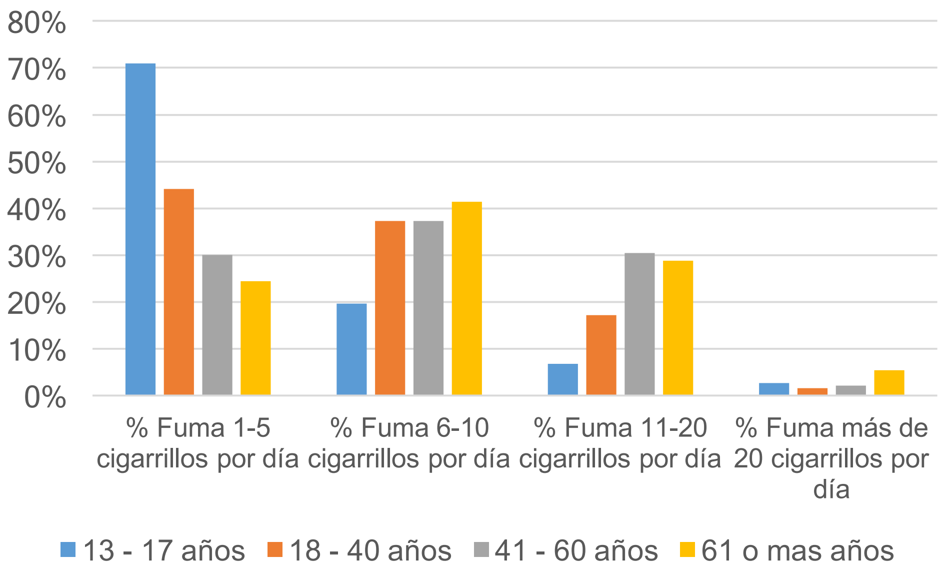

Tabla 15 – Características del Consumo de Cigarrillos por Nivel Educativo

	Sin educación o primaria incompleta		Primaria Completa, Media Incompleta		Media Completa		Alguno Universitario/Técnico		Total	
	Media	Desv. Estándar	Media	Desv. Estándar	Media	Desv. Estándar	Media	Desv. Estándar	Media	Desv. Estándar
% Cigarrillos de Contrabando	30,7%	0,47	27,6%	0,45	11,8%	0,32	5,3%	0,22	10,9%	0,31
% Cigarrillos Saborizados	15,9%	0,37	27,8%	0,45	39,8%	0,49	43,6%	0,50	39,7%	0,49
Cigarrillos diarios promedio	12,6	9,25	10,7	6,63	9,3	6,45	8,6	7,00	9,2	6,87
% Fuma 1-5 cigarrillos por día	23,6%	0,43	25,8%	0,44	34,9%	0,48	43,7%	0,50	37,9%	0,49
% Fuma 6-10 cigarrillos por día	28,7%	0,46	39,4%	0,49	40,9%	0,49	33,9%	0,47	36,9%	0,48
% Fuma 11-20 cigarrillos por día	44,9%	0,51	33,0%	0,47	22,2%	0,42	20,4%	0,40	23,1%	0,42
% Fuma más de 20 cigarrillos por día	2,8%	0,17	1,9%	0,14	2,0%	0,14	2,0%	0,14	2,0%	0,14
Precio por cigarrillos	$119	43,65	$129	50,39	$148	42,69	$157	34,42	$149	40,99

Figura 14 – Características del Consumo de Cigarrillos por Nivel Educativo

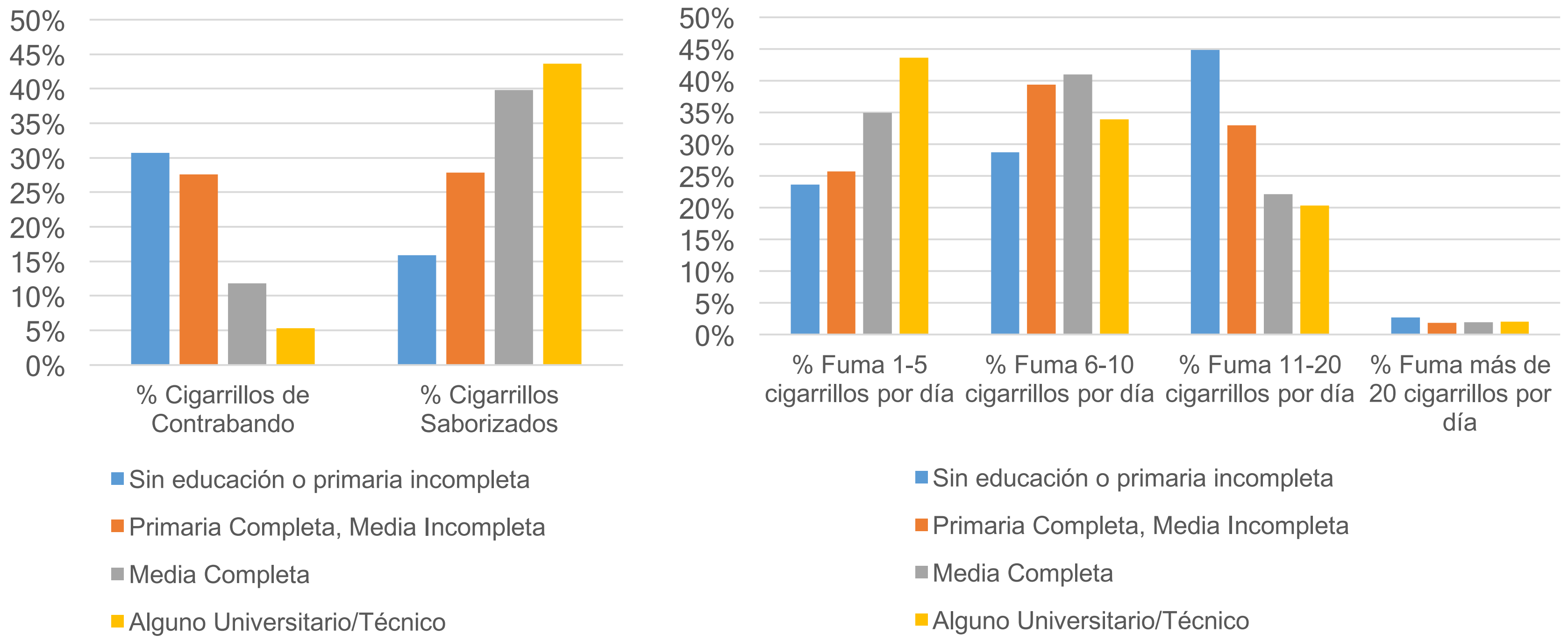

Tabla 16 – Características del Consumo de Cigarrillos por Situación Laboral

	Empleado		Desempleado		Desocupado		Total	
	Media	Desv. Estándar	Media	Desv. Estándar	Media	Desv. Estándar	Media	Desv. Estándar
% Cigarrillos de Contrabando	8,3%	0,28	20,4%	0,41	18,6%	0,39	10,9%	0,31
% Cigarrillos Saborizados	37,6%	0,48	33,8%	0,48	51,6%	0,50	39,7%	0,49
Cigarrillos diarios promedio	9,5	6,90	9,5	6,37	7,7	6,77	9,2	6,87
% Fuma 1-5 cigarrillos por día	35,3%	0,48	34,1%	0,48	51,3%	0,50	37,9%	0,49
% Fuma 6-10 cigarrillos por día	37,6%	0,48	34,4%	0,48	34,9%	0,48	36,9%	0,48
% Fuma 11-20 cigarrillos por día	24,9%	0,43	30,8%	0,47	11,9%	0,32	23,1%	0,42
% Fuma más de 20 cigarrillos por día	2,2%	0,15	0,7%	0,08	1,9%	0,14	2,0%	0,14
Precio por cigarrillos	$151	37,33	$136	42,64	$145	53,28	$149	40,99

Figura 15 – Características del Consumo de Cigarrillos por Situación Laboral

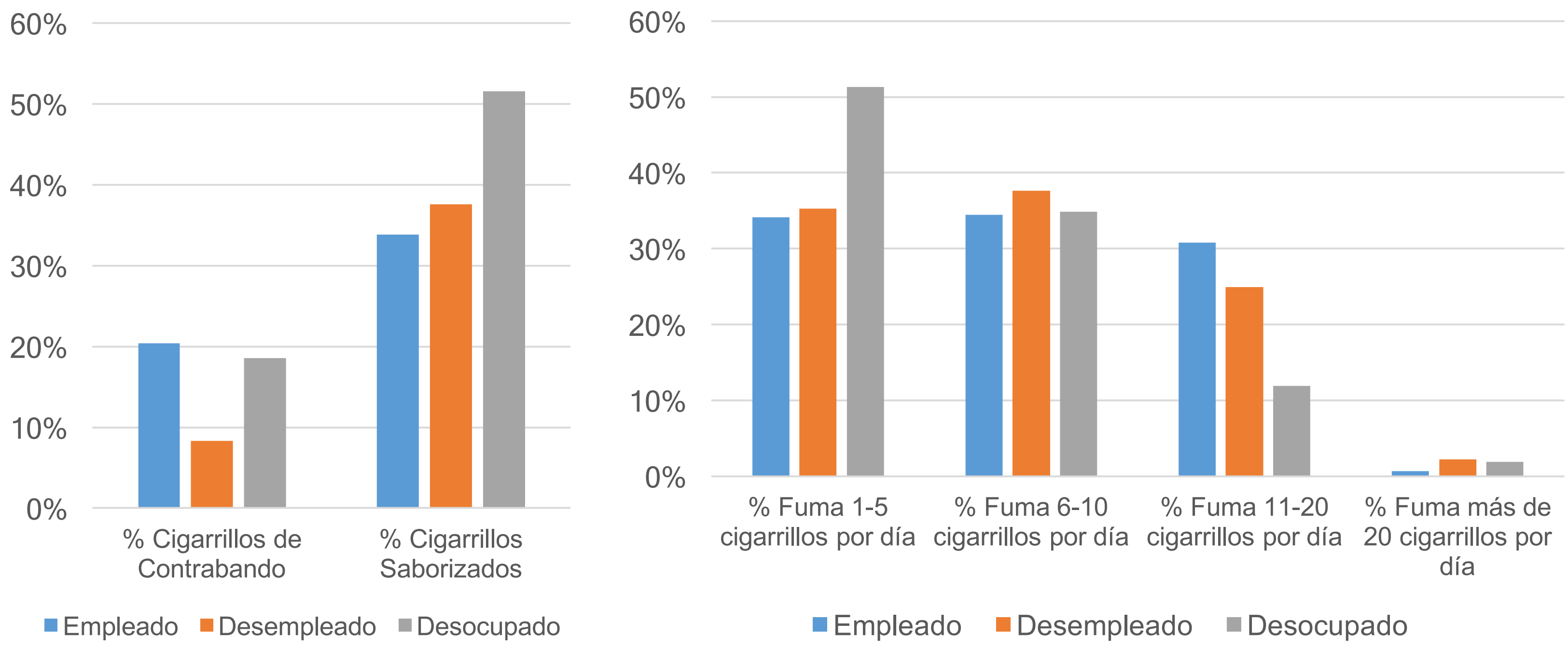

Tabla 17 – Características del Consumo de Cigarrillos por Tiempo de Fumador

	10 o menos años		Entre 11 y 22 años		Entre 23 y 34 años		35 o mas años		Total	
	Media	Desv. Estándar	Media	Desv. Estándar	Media	Desv. Estándar	Media	Desv. Estándar	Media	Desv. Estándar
% Cigarrillos de Contrabando	9,4%	0,29	6,1%	0,24	13,8%	0,35	15,2%	0,36	10,9%	0,31
% Cigarrillos Saborizados	59,3%	0,49	46,9%	0,50	29,9%	0,46	20,8%	0,41	39,7%	0,49
Cigarrillos diarios promedio	6,8	5,86	8,5	5,73	10,6	7,41	11,1	7,62	9,2	6,87
% Fuma 1-5 cigarrillos por día	58,9%	0,49	40,2%	0,49	26,5%	0,44	26,6%	0,44	37,9%	0,49
% Fuma 6-10 cigarrillos por día	29,6%	0,46	38,7%	0,49	38,4%	0,49	40,7%	0,49	36,9%	0,48
% Fuma 11-20 cigarrillos por día	9,1%	0,29	19,5%	0,40	33,5%	0,47	29,8%	0,46	23,1%	0,42
% Fuma más de 20 cigarrillos por día	2,4%	0,15	1,6%	0,12	1,6%	0,13	2,8%	0,17	2,0%	0,14
Precio por cigarrillos	$156	44,49	$156	31,97	$144	43,28	$138	41,86	$149	40,99

Figura 16 – Características del Consumo de Cigarrillos por Tiempo de Fumador

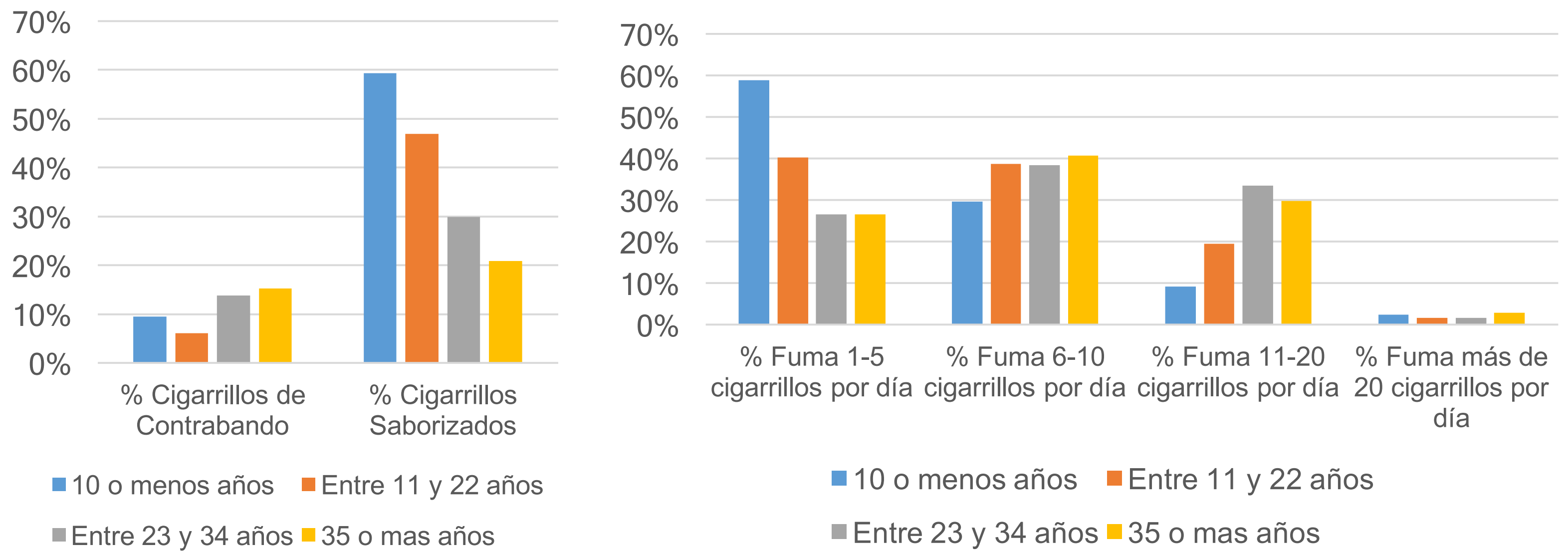

10.4. Análisis Econométrico

Dado que las descripciones previas corresponden a tabulados de medias, en la siguiente sección se realizan regresiones logísticas sobre la probabilidad consumir cigarrillos de contrabando (Tabla 17) y sobre la probabilidad de consumir cigarrillos saborizados (Tabla 18). A diferencia de los tabulados de medias, las regresiones logísticas permiten controlar por múltiples variables (sexo, edad, educación, etc.), pudiendo analizar separadamente las diversas variables asociadas a las decisiones de consumo de cigarrillos. Para cada decisión se estimaron 6 modelos con distintas combinaciones de controles. Entre los controles utilizados se encuentran la edad, la edad al cuadrado (para controlar por efectos no lineales), sexo, edad de inicio en tabaquismo, tiempo siendo fumador, preferencia por precio o sabor (sobre base de preferencia por cigarrillos menos dañinos), situación laboral, logaritmo natural del precio y máximo nivel educativo alcanzado (con base sobre algún estudio universitario/técnico).

Para ambas estimaciones (contrabando y saborizados), los modelos 1, 2 y 3 (que son los preferidos) no incluyen el logaritmo natural del precio que se pagó por la última cajetilla. El primer modelo es el modelo más restringido, sin incluir, ni edad de inicio, ni tiempo de fumador; el modelo 2 incluye edad de inicio y el modelo 3 incluye tiempo como fumador en lugar de edad de inicio. Por su parte, los modelos 4, 5 y 6 siguen la misma dinámica que los modelos 1, 2 y 3, pero incluyendo el logaritmo natural del precio pagado por la última cajetilla.

Sobre la decisión de contrabando (Tabla 17), en los tres modelos sin incluir el precio se observa que los fumadores que tienen preferencia por el precio tienen una mayor probabilidad de elegir cigarrillos de contrabando (sobre los fumadores que tienen preferencia por cigarrillos menos dañinos); quienes tienen preferencia por el precio tienen, en promedio, 24 puntos porcentuales más de probabilidad de elegir cigarrillos de contrabando, que los que prefieren cigarrillos “menos dañinos a la

salud”. También se reportan significativos los efectos sobre la situación laboral, donde los fumadores empleados tienen entre 6,5 y 7 puntos porcentuales menos de probabilidad que los no empleados, de elegir marcas de contrabando. Por otro lado, se observa una relación negativa entre la probabilidad de preferir cigarrillos de contrabando y el nivel educativo; los fumadores sin educación o primaria incompleta reportan, en promedio, 12,4 puntos porcentuales más de probabilidad de consumir marcas de contrabando que los fumadores que presentan algún estudio universitario/técnico, diferencia que disminuye a medida que aumenta el máximo nivel educacional alcanzado.

Respecto a la variable edad, podemos observar una relación negativa; en el modelo 1 por cada año adicional del fumador, la probabilidad de preferir marcas de contrabando aumenta en 0,1 puntos porcentuales. Si bien en el modelo 2 y 3 la edad y la variable edad al cuadrado no se presentan significativas, un test de significancia conjunta reveló que, en el modelo 2, la edad y la edad al cuadrado son significativas conjuntamente al 10%, lo que implicaría que la relación entre la probabilidad de consumir marcas de contrabando podría tener forma de “U”, es decir, en un comienzo, a medida que se incrementa la edad, la probabilidad de consumir cigarrillos de contrabando disminuye, para volver a aumentar en edades posteriores, tal como muestra la Tabla 14.

Luego, a modo de referencia, se agregó como control el logaritmo del precio pagado por cajetilla. Si bien existe endogeneidad por la simultaneidad entre el precio de los cigarrillos y el origen de esta, se decidió incluir estos resultados ya que ilustran una relación entre el precio que se pagó por cajetilla y la probabilidad de que esta sea de contrabando; en promedio, si el precio unitario de los cigarrillos aumenta en un 10%, la probabilidad de que esta sea de contrabando aumenta entre 0,92 y 0,95 puntos porcentuales.

Respecto a la decisión de consumir cigarrillos saborizados (Tabla 18), en los modelos 1, 2 y 3 (que son los preferidos) se observa que, en promedio, si la fumadora es mujer, presenta 13 puntos porcentuales mas de probabilidad de

consumir cigarrillos saborizados. La edad se presenta significativa para los modelos 1, 2, 4 y 5, donde por cada año extra del fumador, la probabilidad de consumir cigarrillos saborizados disminuye en promedio entre 0,9 y 1,9 puntos porcentuales. Si bien las edades al cuadrado no se presentan significativas, en los modelos 2 y 5 las edades se presentan significativas conjuntamente para cualquier nivel de significancia, lo que podría indicar que la disminución en la probabilidad al aumentar la edad es cada vez menor.

También se presentan significativos en la decisión de consumir cigarrillos saborizados las variables de edad de inicio y tiempo de fumador. Para niveles de significancia inferiores al 5%, el tiempo como fumador presenta una relación negativa con la probabilidad de preferir cigarrillos saborizados; cada año extra como fumador disminuye, en promedio, en 0,8 puntos porcentuales la probabilidad de preferir cigarrillos saborizados. Es decir, los fumadores más nuevos tienen más probabilidad de consumir cigarrillos saborizados que los fumadores de mayor edad. También se reportan significativos la edad de inicio (Positiva), cigarrillos consumidos diariamente (negativa), preferencia por el precio (negativa) y tener la primaria completa o media completa sobre algún estudio universitario/técnico (negativo).

Al igual que para la decisión de consumo de marcas de contrabando, en los modelos 4, 5 y 6 se incluyó el logaritmo natural del precio a modo ilustrativo, ya que también existe riesgo de endogeneidad por simultaneidad. Contrariamente a al caso de contrabando, elegir un precio mayor, incremente la probabilidad de que estos sean saborizados; si el precio unitario de los cigarrillos aumenta en un 10%, la probabilidad de que estos sean saborizados aumenta, en promedio, en 1,8 puntos porcentuales.

Tabla 17 – Efectos Marginales Logit Decisión Contrabando[1]

Variable	Modelo 1	Modelo 2	Modelo 3	Modelo 4	Modelo 5	Modelo 6
**Edad**	0,001*<br>(0,0007)	-0,001<br>(0,0035)	-0,002<br>(0,0039)	-0,0003**<br>(0,0002)	0,001<br>(0,0007)	0,002*<br>(0,0008)
**Edad^2**	-<br>-	0,00003<br>(0,00004)	0,00003<br>(0,00004)	-<br>-	-0,00002**<br>(0,00001)	-0,00002**<br>(0,00001)
**Femenino**	-0,029<br>(0,020)	-0,027<br>(0,020)	-0,027<br>(0,020)	-0,017***<br>(0,006)	-0,0179***<br>(0,006)	-0,018***<br>(0,006)
**Edad de Inicio**	-<br>-	-0,001<br>(0,002)	-<br>-	-<br>-	0,001**<br>(0,0003)	-<br>-
**Años como fumador**	-<br>-	-<br>-	0,001<br>(0,002)	-<br>-	-<br>-	-0,001**<br>(0,0002)
**Preferencia por Sabor**	0,029<br>(0,0195)	0,028<br>(0,0194)	0,028<br>(0,0194)	-0,004<br>(0,0063)	-0,004<br>(0,0061)	-0,004<br>(0,0061)
**Preferencia por Precio**	0,237***<br>(0,031)	0,239***<br>(0,031)	0,239***<br>(0,031)	0,008<br>(0,007)	0,007<br>(0,006)	0,007<br>(0,006)
**Cigarrillos diarios consumidos**	0,004***<br>(0,001)	0,004***<br>(0,001)	0,004***<br>(0,001)	0,000<br>(0,0003)	0,000<br>(0,0003)	0,000<br>(0,0003)
**Empleado**	-0,070***<br>(0,018)	-0,065***<br>(0,019)	-0,065***<br>(0,019)	-0,009<br>(0,005)	-0,015**<br>(0,006)	-0,015**<br>(0,006)
**Ln(Precio por cigarrillo)**	-<br>-	-<br>-	-<br>-	-0,092***<br>(0,018)	-0,095***<br>(0,019)	-0,095***<br>(0,019)
**Sin educación o primaria incompleta**	0,124***<br>(0,039)	0,12***<br>(0,039)	0,12***<br>(0,039)	-0,009<br>(0,010)	-0,010<br>(0,009)	-0,010<br>(0,009)
**Primaria Completa, Media Incompleta**	0,114***<br>(0,024)	0,111***<br>(0,025)	0,111***<br>(0,025)	0,003<br>(0,008)	0,002<br>(0,007)	0,002<br>(0,007)
**Media Completa**	0,062***<br>(0,024)	0,062***<br>(0,024)	0,062***<br>(0,024)	0,005<br>(0,005)	0,003<br>(0,004)	0,003<br>(0,004)

***: Significativo al 1%

**: Significativo al 5%

*: Significativo al 10%

1: Errores estándar en paréntesis

Tabla 18 – Efectos Marginales Logit Decisión Saborizados[1]

Variable	Modelo 1	Modelo 2	Modelo 3	Modelo 4	Modelo 5	Modelo 6
**Edad**	-0,009***	-0,018**	-0,0097	-0,008***	-0,019***	-0,0120
	(0,001)	(0,007)	(0,008)	(0,001)	(0,007)	(0,008)
**Edad^2**	-	0,0001	0,0001	-	0,0001	0,0001
	-	(0,0001)	(0,0001)	-	(0,0001)	(0,0001)
**Femenino**	0,130***	0,134***	0,134***	0,132***	0,136***	0,136***
	(0,035)	(0,035)	(0,035)	(0,034)	(0,034)	(0,034)
**Edad de Inicio**	-	0,008**	-	-	0,008**	-
	-	(0,003)	-	-	(0,003)	-
**Años como fumador**	-	-	-0,008**	-	-	-0,008**
	-	-	(0,003)	-	-	(0,003)
**Preferencia por Sabor**	0,0329	0,0293	0,0293	0,0433	0,0402	0,0402
	(0,077)	(0,078)	(0,078)	(0,058)	(0,076)	(0,076)
**Preferencia por Precio**	-0,146*	-0,147*	-0,147*	-0,096	-0,097	-0,097
	(0,081)	(0,081)	(0,081)	(0,082)	(0,082)	(0,082)
**Cigarros diarios consumidos**	-0,007**	-0,006*	-0,006*	-0,006	-0,005	-0,005
	(0,004)	(0,004)	(0,004)	(0,004)	(0,004)	(0,004)
**Empleado**	-0,049	-0,039	-0,039	-0,057	-0,046	-0,046
	(0,041)	(0,044)	(0,044)	(0,041)	(0,044)	(0,044)
**Ln(Precio por cigarrillo)**	-	-	-	0,181***	0,183***	0,183***
	-	-	-	(0,069)	(0,069)	(0,069)
**Sin educación o primaria incompleta**	-0,153	-0,146	-0,146	-0,118	-0,114	-0,114
	(0,119)	(0,123)	(0,123)	(0,118)	(0,123)	(0,123)
**Primaria Completa, Media Incompleta**	-0,130**	-0,135**	-0,135**	-0,107*	-0,113*	-0,114*
	(0,055)	(0,056)	(0,056)	(0,054)	(0,056)	(0,056)
**Media Completa**	-0,010	-0,013	-0,013	-0,001	-0,004	-0,004
	(0,037)	(0,037)	(0,037)	(0,037)	(0,037)	(0,037)

***: Significativo al 1%

**: Significativo al 5%

*: Significativo al 10%

1: Errores estándar en paréntesis

11. Conclusiones

El presente estudio es el primero en lograr estimar, de manera independiente y sin conflictos de interés, la prevalencia de contrabando para el Gran Santiago, por lo que existen diversos resultados que vale la pena destacar.

La prevalencia estimada de cigarrillos de procedencia ilícita en el Gran Santiago durante el tercer trimestre de 2017, es de 10,9% del total de cigarrillos consumidos, cifra que contrasta con el 24% estimada por el Observatorio del Comercio Ilícito, Cámara Nacional de Comercio de Chile y British American Tobacco.[10]

Desagregando por distintas características, se observa que el porcentaje de fumadores que consume cigarrillos ilícitos es mayor en los hombres que en las mujeres (12,9% versus 8,9%). A nivel de edades, los grupos de fumadores de mayor edad son los que presentan la mayor prevalencia de consumo de cigarrillos de contrabando (19,1%), seguidos por los fumadores más jóvenes (16,6%), es decir, los grupos etarios que suelen contar con menores ingresos.

Adicionalmente, se observa que los fumadores más antiguos (los que presentan un mayor nivel de adicción) también presentan un mayor porcentaje de fumadores de marcas de contrabando (15,2%) y, consecuentemente, que los consumidores de cigarrillos de origen ilícito consumen en promedio más cigarrillos diariamente (13,04 versus 8,74). Por último, también resalta que los fumadores que no terminaron la primaria presentan un porcentaje de fumadores de marcas de contrabando más de 5 veces el porcentaje de quienes poseen algún estudio universitario o técnico (30,7% versus 5,3%, respectivamente).

También cabe destacar el precio por cigarrillo de las marcas de procedencia ilegal es menos de la mitad que los cigarrillos de marcas legales; en promedio, el precio por cigarrillo de contrabando es de $61,19 pesos, en contraste con los $160,11

[10] http://www.latercera.com/noticia/cnc-calcula-22-comercio-ilicito-cigarrillos-pais/

pesos de las marcas de contrabando. Adicionalmente, el rango de precios unitarios de las marcas legales va desde $95 pesos a $400 pesos, un rango muy superior al de las marcas de contrabando (entre $49,5 pesos a $160 pesos).

De la estimación econométrica sobre la probabilidad de elegir marcas de contrabando, se encontró que la edad tiene una relación positiva (significativa al 10%) con la probabilidad de elegir marcas de procedencia ilícita; por cada año adicional del fumador, la probabilidad de elegir marcas de contrabando aumenta en promedio 0,1 puntos porcentuales. También se encontró evidencia que la relación podría tener forma de "U", es decir, la probabilidad es mayor en los fumadores más jóvenes, para disminuir en los consumidores de edad media y volver a aumentar a medida que los fumadores entran en edades más avanzadas.

Adicionalmente, las fumadoras mujeres tienen una menor probabilidad de preferir cigarrillos de contrabando (entre 1,7 y 2,9 puntos porcentuales menos que los hombres), al igual que los fumadores empleados (entre 0,9 y 7 puntos porcentuales menos que los no empleados). Los fumadores más intensivos presentan efectos probabilidad promedio superior de preferir marcas de contrabando (0,4 puntos porcentuales más por cada cigarrillo extra consumido diariamente), al igual que quienes tienen preferencia sobre el precio (23,7 puntos porcentuales más que quienes tienen preferencia por marcas menos dañinas). Finalmente, mientras menor sea el nivel educativo del fumador, mayor es la probabilidad de que este decida consumir cigarrillos de contrabando.

De la encuesta también fue posible extraer información respecto a los consumidores de cigarrillos saborizados, los que alcanzan un 39,88% del total de cigarrillos consumidos. Según el análisis a nivel desagregado, las mujeres presentan un mayor porcentaje de fumadoras de cigarrillos saborizados (46,8% versus 32,6% de los hombres). Entre los fumadores más jóvenes, más de la mitad consume cigarrillos saborizados (56,7%), porcentaje que disminuye para fumadores de mayor edad. Adicionalmente, los fumadores más nuevos (menos de 10 años fumando) son los que presentan un mayor porcentaje de consumidores de cigarrillos saborizados

(58,9%), mientras que, a mayor tiempo como fumador, menor es el porcentaje de fumadores que prefiere variedades saborizadas.

De la estimación econométrica sobre la probabilidad de elegir cigarrillos saborizados, se encontró que la edad presenta una relación negativa con la probabilidad de preferir estas variedades, así como también quienes tienen menos años de fumador presentan probabilidades de preferir cigarrillos saborizados. Finalmente, se observa que las mujeres presentan en promedio probabilidades menores que los hombres de preferir variedades de contrabando y que los fumadores más intensivos (mayor consumo de cigarrillos diarios) tienen una probabilidad promedio menor de elegir cigarrillos saborizados, así como también los que tienen preferencias sobre el precio.

12. Anexos

12.1. Cuestionario

<u>Encuesta a personas que consumen cigarrillo y sus hábitos</u>

LEER:

Buenos días/tardes/noches, mi nombre es ___________________ (ENCUESTADOR POR FAVOR: MENCIONE SU NOMBRE) y fui contratado por un equipo de investigación para la realización de un estudio para la American Cancer Society y la Universidad Adolfo Ibáñez. Con el fin de mejorar sus conocimientos sobre patrones de consumo de tabaco, estas instituciones están interesadas en realizarle algunas preguntas. Es importante mencionar que su identidad y comentarios siempre serán mantenidos en estricta confidencialidad, únicamente el equipo de investigación tendrá acceso al material de las encuestas. La información que será reportada a los usuarios de la investigación aparecerá de manera agregada (general), sin relacionar opiniones con individuos.
La información con la que pueda proveernos es sumamente valiosa.

Número de Formulario	Fecha Encuesta			Encuestador
	día	mes	año	

Parte I – Datos Básicos

	0. ¿Usted reside en Santiago?<br>1. Sí<br>2. No →FIN	1. ¿Estaría dispuesto a contestas nuestras preguntas?<br>1. Sí<br>2. No→FIN	2. Fecha de Nacimiento<br>*Anote día, mes y año.*		3. Género<br>1. Masculino<br>2. Femenino
**Pregunta**	**0**	**1**	**2_día**	**2_mes**	**2_año**	**3**
Respuesta						

Parte II – Comportamiento y Actitudes de Tabaquismo

Encuestador, en este módulo se encuentran las preguntas que realizan la caracterización del comportamiento y actitud del encuestado sobre el hábito de fumar.
<u>Para fumadores adultos (nacidos antes del 1999)</u> **LEER: Ahora me gustaría hacerle algunas preguntas acerca del hábito de fumar.**
<u>Para los menores y jóvenes (nacidos en 1999 o posterior)</u> **LEER: Ahora me gustaría hacerle preguntas acerca del hábito de fumar. Entiendo que los demás pueden pensar que usted no debería fumar, SIN EMBARGO LO QUE USTED ME DIGA ES ABSOLUTAMENTE PRIVADO y es importante reconocer lo que se hace en la realidad, para evaluar los hábitos y salud de los fumadores.**

	4. Actualmente fuma:<br>a) Cigarrillo<br>b) Tabaco<br>c) Otro<br>Responda, para cada uno:<br>1. Sí<br>2. No<br>Si responde SI en cigarrillo, continúe la encuesta. De lo contrario, finalícela.			4.1 ¿Con qué frecuencia o cada cuánto fuma cigarrillos?<br>1. Diariamente → ¿Cuántos al día?<br>2. Algunos días a la semana →¿Cuántos a la semana?<br>3. Menos de una vez a la semana → ¿Cuántos al mes?		5. ¿A qué edad probó usted el cigarrillo por primera vez?<br>Anote edad<br>88. No recuerda<br>99. No responde
**Pregunta**	**3_a**	**3_b**	**3_c**	**4_1**	**4_1_Cuantos**	**5_edad**
Respuesta						

1

Parte II – Comportamiento y Actitudes de Tabaquismo				
	6. ¿Qué marca de cigarrillos fumó la última vez?<br><br>*Buscar en la lista adjunta el Código dela Marca y anótelo.*	7. ¿Podría mostrarnos la **cajetilla** de cigarrillos que tiene en este momento? Es con el fin de obtener más información de su marca.<br>1. Sí<br>2. No →pase a 9<br>3. No tengo cajetilla, solo unidad →pase a 9	7.1 ¿Me permite tomar una foto de la cajetilla?<br>1. Sí<br>2. No → pase a 9<br><br>*Encuestador: tomar dos fotos:*<br>*a) A una de las caras con la advertencia sanitaria y la marca*<br>*b) A la cara lateral que indica el lugar de fabricación*<br><br>7.2 Registre el país de fabricación.	
**Pregunta**	**6**	**7**	**7_1**	**7_2_País**
Respuesta				

Parte II – Comportamiento y Actitudes de Tabaquismo				
	Encuestador: Verifique si en la cara lateral aparece el texto:<br>8.1 Producto importado para Chile<br>1. Sí<br>2. No<br><br>8.2 Registre si la cajetilla tiene:<br>a. *Tiene una advertencia vigente en Chile de la ronda 2016.*<br>b. *Tiene una advertencia vigente en Chile de la ronda 2015.*<br>c. *Tiene una advertencia vigente en Chile de rondas anteriores.*<br>d. *Es la advertencia de otro país.*<br>e. *No tiene advertencia.*		Ahora le haré unas preguntas sobre los lugares que venden cigarrillos, sobre la marca de su última compra y los precios de esa compra.<br><br>9. La última vez que compró para usted, ¿Dónde compró los cigarrillos?<br>1. Tienda de barrio.<br>2. Tabaquería, botillería.<br>3. Vendedor ambulante.<br>4. Estación de servicio.<br>5. Supermercado.<br>6. Bar, restaurante o confitería.<br>7. Fura del país.<br>8. Zona franca, duty free.<br>9. No recuerda / No sabe<br>10. Otro. Especifique.	
**Pregunta**	**8_1**	**8_2**	**9**	**9_especifique**
Respuesta				

Parte II – Comportamiento y Actitudes de Tabaquismo				
	10. En el sitio mencionado en el punto anterior, ¿Qué marca compró?<br><br>*Anote respuesta acorde a la lista códigos.*<br><br>99. No recuerda / No sabe.	11. La última vez que compró cigarrillos para usted, ¿compró:<br><br>1. Una cajetilla de cigarros →pase a 12<br>2. Cigarrillos sueltos → pase a 13<br>3. Un cartón → pase a 14	12. Ahora piense en el pago por cajetilla de cigarrillos, ¿Cuánto pagó por la última cajetilla de cigarrillos que compró para usted?<br><br>*Anote monto en pesos.*<br><br>99. No sabe / No responde	12.1 ¿Cuántos cigarrillos tenía la última cajetilla que compró?<br><br>*Anote número*<br><br>99. No sabe / No responde
**Pregunta**	**10**	**11**	**12**	
Respuesta				

Parte II – Comportamiento y Actitudes de Tabaquismo			
	13. ¿Cuánto por el total de cigarrillos sueltos en la última compra?<br><br>*Anote monto en pesos.*<br><br>99. No sabe / No responde	13.1 ¿Cuántos cigarrillos sueltos compró la última vez que compró cigarrillos sueltos para usted?<br><br>*Anote número de cigarrillos.*<br><br>99. No sabe / No responde	14. ¿Cuánto pagó por el cartón?<br><br>*Anote monto en pesos.*<br><br>99. No sabe / No responde
**Pregunta**	**13**	**13_1**	**14**
Respuesta			

Parte II – Comportamiento y Actitudes de Tabaquismo			
	14.1 ¿Cuántas cajetillas tenía el cartón?<br><br>*Anote número de cajetillas.*<br><br>99. No sabe / No responde	*14.2* ¿Cuántos cigarrillos tenía cada cajetilla?<br><br>*Anote número de cigarrillos.*<br><br>99. No sabe / No responde	15. Cuando eligió la marca de los cigarrillos en su última compra, pensó principalmente en:<br><br>1. Sabor<br>2. En que la causaría menos daño<br>3. Precio
**Pregunta**	**14_1**	**14_2**	**15**
Respuesta			

Parte II – Comportamiento y Actitudes de Tabaquismo			
	16. ¿Es la marca de su última compra su marca favorita?<br><br>1. Sí → pase a 19<br>2. No	*17. ¿Cuál es su marca de cigarrillos favorita?*<br><br>*Buscar en la lista adjunta, el código de la marca.*	18. Al elegir [nombre de la marca favorita] como su favorita, usted toma su decisión en base a:<br><br>1. Sabor<br>2. En que la causaría menos daño<br>3. Precio
**Pregunta**	**16**	**17**	**18**
Respuesta			

<table>
<tr><th colspan="5">Parte III – Características Socioeconómicas</th></tr>
<tr><td colspan="5">Encuestador, en este módulo se encuentran las preguntas que realizan la caracterización socioeconómica de los encuestado.
LEER:
Ahora, ¿Podría proporcionarme algunos datos adicionales sobre usted?</td></tr>
<tr><td></td><td colspan="2">19. ¿Cuál es el nivel educativo más alto que usted ha alcanzado?

Anote máximo nivel o n° de años

1. Ninguno
2. Preescolar
3. Enseñanza Básica ciclo I (1° - 4°)
4. Enseñanza Básica ciclo II (5° - 8°)
5. Enseñanza Media (I° - IV°)
6. Técnico sin título
7. Técnico con título
8. Universitario sin título
9. Universidad con título
10. Posgrado sin título
11. Posgrado con título</td><td>20. ¿En qué actividad ocupó la mayor parte del tiempo de la semana pasada?

1. Trabajando
2. Buscando trabajo
3. Pensionado
4. Estudiando
5. Oficios del hogar
6. Incapacitado permanente para trabajar
7. Otra actividad. Especifique.
(2–7) Pase a 22</td><td>21. Es usted:

1. Asalariado (Empleado u obrero)
2. Independiente o Trabajador por Cuenta Propia.</td></tr>
<tr><td>Pregunta</td><td>19</td><td>19_años</td><td>20</td><td>21</td></tr>
<tr><td>Respuesta</td><td></td><td></td><td></td><td></td></tr>
</table>

<table>
<tr><th colspan="6">Parte III – Características Socioeconómicas</th></tr>
<tr><td></td><td>22. ¿Cuál es su comuna de residencia?

Buscar en la lista adjunta el CÓDIGO DE COMUNA</td><td colspan="2">23.1 Lugar de la Encuesta: Comuna

23.2 Lugar de la Encuesta: Dirección</td><td colspan="2">24 Sitio de la Encuesta

1. Tienda de Barrio
2. Salida Centro Educativo
3. Centro de Ciudad
4. Centro Comercial
5. Cafetería
6. Otro. Especifique.</td></tr>
<tr><td>Pregunta</td><td>16</td><td>23_1</td><td>23_2</td><td>24</td><td>24_Especifique</td></tr>
<tr><td>Respuesta</td><td></td><td></td><td></td><td></td><td></td></tr>
</table>

12.2. Hoja de ruta

CENTRO MICRO DATOS

Hoja de Ruta – Consumo de Cigarrillos

**Dirección Referencia de**		**Comuna:**	

**Objetivo Hombres**				**Objetivo Mujeres**			
**Menos de 18 años**	**19 a 40**	**41 a 60**	**61 y +**	**Menos de 18 años**	**19 a 40**	**41 a 60**	**61 y +**

**Logradas Hombres**				**Logradas Mujeres**			
**Menos de 18 años**	**19 a 40**	**41 a 60**	**61 y +**	**Menos de 18 años**	**19 a 40**	**41 a 60**	**61 y +**

**Fecha de Aplicación Día 1 (Día y Mes)**			**Horario de Aplicación Día 1 (Indicar Hora de Inicio y Término)**			
**Día**	**Mes**	**Año**	**Hora inicio**	**Minut**	**Hora fin**	**Minuto fin**
**Fecha de Aplicación Día 2 (Día y Mes)**			**Horario de Aplicación Día 2 (Indicar Hora de Inicio y Término)**			
**Día**	**Mes**	**Año**	**Hora inicio**	**Minut**	**Hora fin**	**Minuto fin**
**Fecha de Aplicación Día 3 (Día y Mes)**			**Horario de Aplicación Día 3 (Indicar Hora de Inicio y Término)**			
**Día**	**Mes**	**Año**	**Hora inicio**	**Minut**	**Hora fin**	**Minuto fin**
**Fecha de Aplicación Día 4 (Día y Mes)**			**Horario de Aplicación Día 4 (Indicar Hora de Inicio y Término)**			
**Día**	**Mes**	**Año**	**Hora inicio**	**Minut**	**Hora fin**	**Minuto fin**

**Nombre Encuestador:**	
**Nombre Coordinador:**	
**Observaciones:**	

<u>Mapa de la Manzana Seleccionada:</u> A continuación se muestran una imagen con el punto geográfico en el que usted deberá encuestar. El área en azul es aquella en la que usted puede realizar encuestas. El punto rojo sirve como referencia de un lugar en el que fluye el transporte público. En la parte inferior se muestra la manzana seleccionada en un mapa más grande.

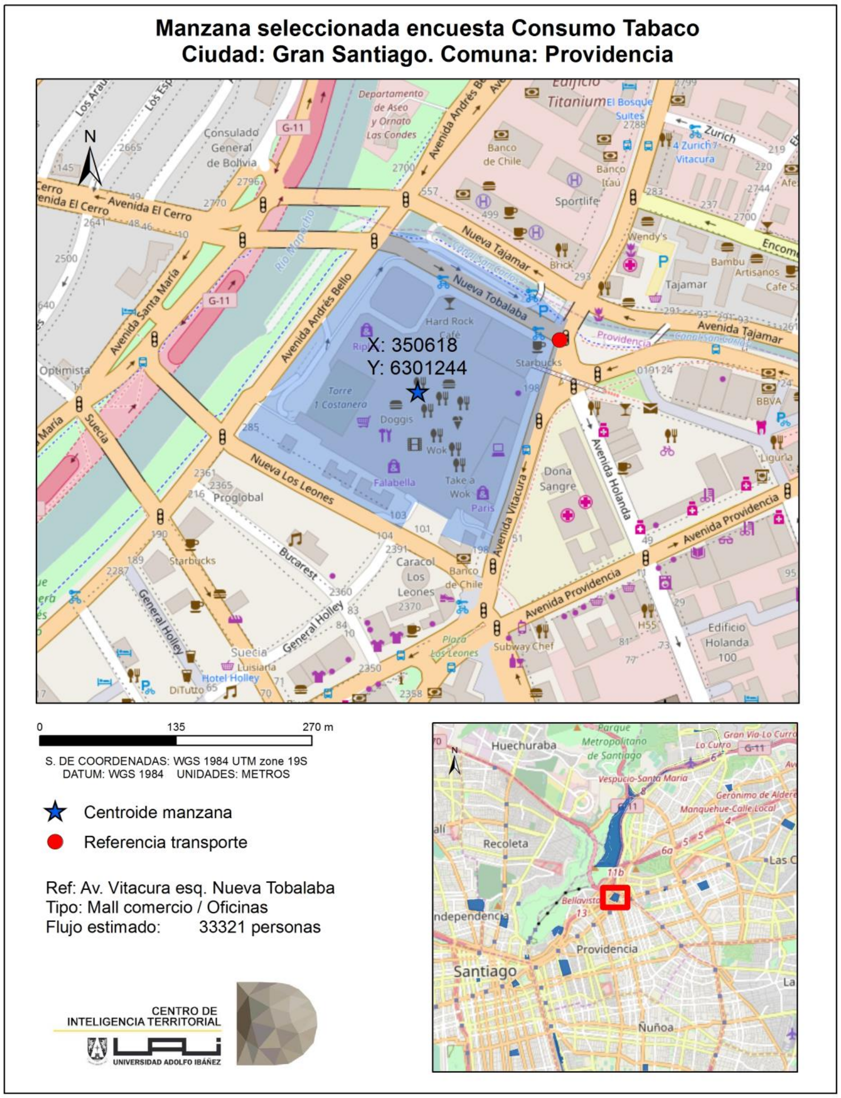

12.3. Tabulación simple de los resultados

Módulo A – Datos Básicos

A0. ¿Usted reside en la Provincia de Santiago?

a0	Freq.	Percent	Cum.
Si	857	100.00	100.00
Total	857	100.00	

A1. ¿Estaría dispuesto a contestar nuestras preguntas?

a1	Freq.	Percent	Cum.
Si	857	100.00	100.00
Total	857	100.00	

A2. Fecha de nacimiento

a2_dia	Freq.	Percent	Cum.
1	35	4.08	4.08
2	31	3.62	7.70
3	31	3.62	11.32
4	36	4.20	15.52
5	40	4.67	20.19
6	25	2.92	23.10
7	26	3.03	26.14
8	37	4.32	30.46
9	19	2.22	32.67
10	45	5.25	37.92
11	26	3.03	40.96
12	31	3.62	44.57
13	14	1.63	46.21
14	26	3.03	49.24
15	22	2.57	51.81
16	26	3.03	54.84
17	23	2.68	57.53
18	33	3.85	61.38
19	35	4.08	65.46
20	37	4.32	69.78

21	28	3.27	73.05
22	20	2.33	75.38
23	37	4.32	79.70
24	16	1.87	81.56
25	27	3.15	84.71
26	30	3.50	88.21
27	19	2.22	90.43
28	24	2.80	93.23
29	24	2.80	96.03
30	25	2.92	98.95
31	9	1.05	100.00
Total	857	100.00	

a2_mes	Freq.	Percent	Cum.
Enero	80	9.33	9.33
Febrero	70	8.17	17.50
Marzo	70	8.17	25.67
Abril	63	7.35	33.02
Mayo	77	8.98	42.01
Junio	72	8.40	50.41
Julio	58	6.77	57.18
Agosto	71	8.28	65.46
Septiembre	85	9.92	75.38
Octubre	63	7.35	82.73
Noviembre	69	8.05	90.78
Diciembre	79	9.22	100.00
Total	857	100.00	

a2_ao	Freq.	Percent	Cum.
1929	1	0.12	0.12
1931	1	0.12	0.23
1933	1	0.12	0.35
1940	1	0.12	0.47
1941	2	0.23	0.70
1942	2	0.23	0.93
1943	3	0.35	1.28
1945	3	0.35	1.63
1946	3	0.35	1.98
1947	1	0.12	2.10
1948	5	0.58	2.68
1949	2	0.23	2.92
1950	8	0.93	3.85
1951	4	0.47	4.32
1952	14	1.63	5.95
1953	9	1.05	7.00
1954	10	1.17	8.17
1955	17	1.98	10.15
1956	10	1.17	11.32
1957	7	0.82	12.14

1958	12	1.40	13.54
1959	5	0.58	14.12
1960	25	2.92	17.04
1961	18	2.10	19.14
1962	17	1.98	21.12
1963	14	1.63	22.75
1964	14	1.63	24.39
1965	20	2.33	26.72
1966	6	0.70	27.42
1967	13	1.52	28.94
1968	19	2.22	31.16
1969	23	2.68	33.84
1970	19	2.22	36.06
1971	16	1.87	37.92
1972	15	1.75	39.67
1973	14	1.63	41.31
1974	20	2.33	43.64
1975	30	3.50	47.14
1976	13	1.52	48.66
1977	20	2.33	50.99
1978	9	1.05	52.04
1979	17	1.98	54.03
1980	15	1.75	55.78
1981	18	2.10	57.88
1982	21	2.45	60.33
1983	15	1.75	62.08
1984	14	1.63	63.71
1985	16	1.87	65.58
1986	28	3.27	68.84
1987	16	1.87	70.71
1988	16	1.87	72.58
1989	20	2.33	74.91
1990	14	1.63	76.55
1991	13	1.52	78.06
1992	16	1.87	79.93
1993	10	1.17	81.10
1994	10	1.17	82.26
1995	12	1.40	83.66
1996	18	2.10	85.76
1997	15	1.75	87.51
1998	11	1.28	88.80
1999	22	2.57	91.37
2000	46	5.37	96.73
2001	14	1.63	98.37
2002	11	1.28	99.65
2003	3	0.35	100.00
Total	857	100.00	

A3. Género

a3	Freq.	Percent	Cum.

```
  Masculino |        431       50.29       50.29
   Femenino |        426       49.71      100.00
------------+-----------------------------------
      Total |        857      100.00

-------------------------------------------------------------------
```

Módulo B – Comportamiento y Actitudes de Tabaquismo

B4. Actualmente fuma

b4	Freq.	Percent	Cum.
Cigarrillo	857	100.00	100.00
Total	857	100.00	

B4.1 ¿Con qué frecuencia o cada cuánto fuma cigarrillos?

b4_1	Freq.	Percent	Cum.
Diariamente	801	93.47	93.47
Algunos días a la semana	55	6.42	99.88
Menos de una vez al mes	1	0.12	100.00
Total	857	100.00	

b4_1_esp	Freq.	Percent	Cum.
1	11	1.28	1.28
2	50	5.83	7.12
3	79	9.22	16.34
4	60	7.00	23.34
5	125	14.59	37.92
6	47	5.48	43.41
7	30	3.50	46.91
8	44	5.13	52.04
9	6	0.70	52.74
10	187	21.82	74.56
12	25	2.92	77.48
13	1	0.12	77.60
14	4	0.47	78.06
15	65	7.58	85.65
18	4	0.47	86.11
20	99	11.55	97.67
25	3	0.35	98.02
30	7	0.82	98.83
40	6	0.70	99.53
50	2	0.23	99.77
60	2	0.23	100.00
Total	857	100.00	

B5. ¿A qué edad probó el cigarrillo por primera vez?

b5	Freq.	Percent	Cum.
1	837	97.67	97.67
2	18	2.10	99.77
3	2	0.23	100.00
Total	857	100.00	

b5_esp	Freq.	Percent	Cum.
6	1	0.12	0.12
7	1	0.12	0.24
8	2	0.24	0.48
9	6	0.72	1.19
10	6	0.72	1.91
11	24	2.87	4.78
12	47	5.62	10.39
13	71	8.48	18.88
14	117	13.98	32.86
15	125	14.93	47.79
16	80	9.56	57.35
17	77	9.20	66.55
18	97	11.59	78.14
19	33	3.94	82.08
20	66	7.89	89.96
21	15	1.79	91.76
22	12	1.43	93.19
23	5	0.60	93.79
24	7	0.84	94.62
25	10	1.19	95.82
26	6	0.72	96.54
27	3	0.36	96.89
28	4	0.48	97.37
29	1	0.12	97.49
30	9	1.08	98.57
31	1	0.12	98.69
32	1	0.12	98.81
35	1	0.12	98.92
37	1	0.12	99.04
40	3	0.36	99.40
45	1	0.12	99.52
47	1	0.12	99.64
57	3	0.36	100.00
Total	837	100.00	

B6. ¿Qué marca de cigarrillos fumó la última vez?

b6	Freq.	Percent	Cum.
Belmont	40	4.67	4.67
Business Class	2	0.23	4.90
Carnival	15	1.75	6.65
Dunhill	17	1.98	8.63
Esse	9	1.05	9.68
Fox	23	2.68	12.37
Hilton	16	1.87	14.24
Indy	1	0.12	14.35
Jaisalmer	16	1.87	16.22
JL	19	2.22	18.44
Kent	103	12.02	30.46
Latino	2	0.23	30.69
Lucky Strike	165	19.25	49.94
Marlboro	8	0.93	50.88
Nirvana	4	0.47	51.34
Pall Mall	377	43.99	95.33
Parliament	2	0.23	95.57
Phillip Morris	14	1.63	97.20
Pine	13	1.52	98.72
Viceroy	1	0.12	98.83
Otro Especifique (You, Miles, etc)	10	1.17	100.00
Total	857	100.00	

B7. ¿Podría mostrarnos la cajetilla de cigarrillos que tiene en este momento? Es con el fin de obtener más información de su marca.

b7	Freq.	Percent	Cum.
Si	857	100.00	100.00
Total	857	100.00	

B7_f. ¿Me permite tomar una foto de la cajetilla?

b7_f	Freq.	Percent	Cum.
Si	857	100.00	100.00
Total	857	100.00	

B7_1. Registre el país de fabricación

b7_1	Freq.	Percent	Cum.
Argentina	18	2.10	2.10
Bolivia	14	1.63	3.73
Brasil	1	0.12	3.85
Chile	733	85.53	89.38
Corea del Sur	30	3.50	92.88
Estados Unidos	10	1.17	94.05
India	13	1.52	95.57
Italia	1	0.12	95.68
Paraguay	21	2.45	98.13
Perú	1	0.12	98.25
Uruguay	2	0.23	98.48
Vietnam	3	0.35	98.83
No especifica	10	1.17	100.00
Total	857	100.00	

B8_1. Encuestador: Verifique si en la cara lateral aparece el texto: Producto importado para Chile

b8_1	Freq.	Percent	Cum.
Si	473	55.19	55.19
No	384	44.81	100.00
Total	857	100.00	

B8_2. Registre si la cajetilla tiene:

b8_2	Freq.	Percent	Cum.
Tiene una advertencia vigente en chile	750	87.51	87.51
Tiene una advertencia vigente en Chile	7	0.82	88.33
Tiene una advertencia vigente en Chile	4	0.47	88.80
Es la advertencia de otro país	86	10.04	98.83
No tiene advertencia	10	1.17	100.00
Total	857	100.00	

B9. ¿La última vez que compró para usted, ¿en dónde compro los cigarrillos?

b9	Freq.	Percent	Cum.
Tienda de barrio.	361	42.12	42.12
Tabaquería, botillería.	163	19.02	61.14
Vendedor ambulante.	81	9.45	70.60
Estación de servicio.	62	7.23	77.83
Supermercado.	80	9.33	87.16
Bar, restaurante o confitería.	15	1.75	88.91
Fuera del país.	3	0.35	89.26
Zona franca, duty free.	5	0.58	89.85
Otro. Especifique.	81	9.45	99.30
No recuerda / No sabe	6	0.70	100.00
Total	857	100.00	

b9_esp	Freq.	Percent	Cum.
	776	90.55	90.55
a una compañera	1	0.12	90.67
a una compañera de trabajo	1	0.12	90.78
en el metro	1	0.12	90.90
en la calle	1	0.12	91.02
kiosko	68	7.93	98.95
le saco la cajetilla a la mama no sab..	1	0.12	99.07
no compra , sus cigarrillos son propo..	1	0.12	99.18
no sabe, fue obsequiada por pareja	1	0.12	99.30
panaderia	1	0.12	99.42
regalado	3	0.35	99.77
se la saco al hermano	1	0.12	99.88
se lo compro la hermana , no sabe donde	1	0.12	100.00
Total	857	100.00	

B10. En el sitio mencionado en el punto anterior, ¿Qué marca compró?

b10	Freq.	Percent	Cum.
Belmont	34	3.97	3.97
Business Class	2	0.23	4.20
Carnival	14	1.63	5.83
Dunhill	7	0.82	6.65
Esse	9	1.05	7.70
Fox	24	2.80	10.50
Jaisalmer	18	2.10	12.60
Kent	120	14.00	26.60
Latino	2	0.23	26.84
Lucky Strike	184	21.47	48.31
Marlboro	10	1.17	49.47
Nirvana	4	0.47	49.94
Pall Mall	393	45.86	95.80
Phillip Morris	14	1.63	97.43
Pine	11	1.28	98.72
Otro Especifique (You, Miles, etc)	9	1.05	99.77
No especifica	2	0.23	100.00
Total	857	100.00	

b10_esp	Freq.	Percent	Cum.
	848	98.95	98.95
Roma	1	0.12	99.07
black jack	2	0.23	99.30
fiesta	1	0.12	99.42
miles	2	0.23	99.65
ruby	1	0.12	99.77
this	1	0.12	99.88
you	1	0.12	100.00
Total	857	100.00	

B11. La última vez que compró cigarrillos para usted, ¿compró:

b11	Freq.	Percent	Cum.
Una cajetilla de cigarros	846	98.72	98.72
Cigarrilos sueltos	2	0.23	98.95
Un cartón	9	1.05	100.00
Total	857	100.00	

B12. Ahora piense en el pago por cajetilla de cigarrillos. ¿Cuánto pagó por la última cajetilla de cigarrillos que compró para usted?

b12	Freq.	Percent	Cum.
No sabe o no responde	14	1.65	1.65
1000	49	5.79	7.45
1100	2	0.24	7.68
1200	7	0.83	8.51
1300	9	1.06	9.57
1400	8	0.95	10.52
1500	66	7.80	18.32
1600	12	1.42	19.74
1700	62	7.33	27.07
1800	48	5.67	32.74
1900	21	2.48	35.22
2000	34	4.02	39.24
2100	3	0.35	39.60
2200	1	0.12	39.72
2300	5	0.59	40.31
2400	1	0.12	40.43
2500	6	0.71	41.13
2600	74	8.75	49.88
2700	15	1.77	51.65
2800	18	2.13	53.78
2900	133	15.72	69.50
3000	44	5.20	74.70
3100	27	3.19	77.90
3108	1	0.12	78.01
3200	19	2.25	80.26
3300	53	6.26	86.52
3400	41	4.85	91.37
3500	38	4.49	95.86
3600	22	2.60	98.46
3700	3	0.35	98.82
3800	7	0.83	99.65
4000	2	0.24	99.88
4400	1	0.12	100.00
Total	846	100.00	

B12_1. ¿Cuántos cigarrillos tenía la última cajetilla que compró?

b12_1_monto	Freq.	Percent	Cum.
10	232	27.42	27.42
20	614	72.58	100.00
Total	846	100.00	

B13. Pago por cigarrillos sueltos ¿Cuánto pagó por el total de cigarrillos sueltos en la última compra?

b13	Freq.	Percent	Cum.
No sabe o no responde	1	50.00	50.00
800	1	50.00	100.00
Total	2	100.00	

B13_1. ¿Cuántos cigarrillos sueltos compró la última vez que compró cigarrillos sueltos?

b13_1	Freq.	Percent	Cum.
4	1	50.00	50.00
No sabe o no responde	1	50.00	100.00
Total	2	100.00	

B14. ¿Cuánto pagó por el cartón?

b14	Freq.	Percent	Cum.
No sabe o no responde	1	11.11	11.11
10000	1	11.11	22.22
15000	1	11.11	33.33
20000	1	11.11	44.44
23000	1	11.11	55.56
27000	2	22.22	77.78
33000	1	11.11	88.89
35000	1	11.11	100.00
Total	9	100.00	

B14_1. ¿Cuántas cajetillas tenía el cartón?

b14_1	Freq.	Percent	Cum.
10	8	88.89	88.89
20	1	11.11	100.00
Total	9	100.00	

B14.2 ¿Cuántos cigarrillos tenía cada cajetilla?

b14_2	Freq.	Percent	Cum.
20	9	100.00	100.00
Total	9	100.00	

B15. Cuando eligió la marca de los cigarrillos en su última compra pensó principalmente en:

b15	Freq.	Percent	Cum.
Sabor	564	65.81	65.81
En que le causaría menos daño	64	7.47	73.28
Precio	229	26.72	100.00
Total	857	100.00	

B16. ¿Es la marca de su última compra su marca favorita?

b16	Freq.	Percent	Cum.
Si	684	79.81	79.81
No	173	20.19	100.00
Total	857	100.00	

B17. ¿Cuál es su marca favorita de cigarrillos?

b17	Freq.	Percent	Cum.
BELMONT	18	10.40	10.40
CAMEL	2	1.16	11.56
CARNIVAL	1	0.58	12.14
DUNHILL	2	1.16	13.29
ESSE	1	0.58	13.87
FOX	2	1.16	15.03
JL	1	0.58	15.61
KENT	25	14.45	30.06
LUCKY STRICKE	59	34.10	64.16
MARLBORO	16	9.25	73.41
PALL MALL	32	18.50	91.91
PHILLIP MORRIS	3	1.73	93.64
VICEROY	5	2.89	96.53
YOU	6	3.47	100.00
Total	173	100.00	

B18. Al elegir (opción elegida) como su favorita, usted tomó su decisión en base a:

b18	Freq.	Percent	Cum.
Sabor	160	92.49	92.49
En que le causaría menos daño	7	4.05	96.53
Precio	6	3.47	100.00
Total	173	100.00	

Módulo C – Características Socioecomómicas

C19. ¿Cuál es el nivel educativo más alto que usted ha alcanzado?

c19_nivel	Freq.	Percent	Cum.
Enseñanza básica ciclo I (1° - 4°)	11	1.28	1.28
Enseñanza básica ciclo II (5° - 8°)	60	7.00	8.28
Enseñanza Media (I° - IV°)	393	45.86	54.14
Técnico sin título	54	6.30	60.44
Técnico con título	124	14.47	74.91
Universitario sin título	73	8.52	83.43
Universidad con título	133	15.52	98.95
Posgrado sin título	3	0.35	99.30
Posgrado con título	6	0.70	100.00
Total	857	100.00	

c19_curso	Freq.	Percent	Cum.
1	53	6.18	6.18
2	129	15.05	21.24
3	155	18.09	39.32
4	338	39.44	78.76
5	102	11.90	90.67
6	23	2.68	93.35
7	11	1.28	94.63
8	45	5.25	99.88
10	1	0.12	100.00
Total	857	100.00	

C20. ¿En qué actividad ocupó la mayor parte del tiempo la semana pasada?

c20	Freq.	Percent	Cum.
Trabajando	600	70.01	70.01
Buscando Trabajo	49	5.72	75.73
Pensionado	36	4.20	79.93
Estudiando	115	13.42	93.35
Oficios del hogar	48	5.60	98.95
Incapacitado permanente para trabajar	5	0.58	99.53
Otra Actividad. Especifique	4	0.47	100.00
Total	857	100.00	

c20_esp	Freq.	Percent	Cum.
	851	99.53	99.53
no hace nada	2	0.23	99.77
no hace nada el próximo año va a hace..	1	0.12	99.88
no quiere estudiar, no hace nada	1	0.12	100.00
Total	855	100.00	

C21. Es usted:

c21	Freq.	Percent	Cum.
Asalariado (empleado u obrero)	442	73.67	73.67
Independiente o Trabajador por Cuenta P	158	26.33	100.00
Total	600	100.00	

C22. ¿Cuál es su comuna de residencia?

c22	Freq.	Percent	Cum.
Santiago	66	7.70	7.70
Cerrillos	17	1.98	9.68
Cerro Navia	18	2.10	11.79
Conchalí	10	1.17	12.95
El Bosque	20	2.33	15.29
Estación Central	27	3.15	18.44
Huechuraba	17	1.98	20.42
Independencia	22	2.57	22.99
La Cisterna	17	1.98	24.97
La Florida	50	5.83	30.81
La Granja	12	1.40	32.21
La Pintana	16	1.87	34.07
La Reina	9	1.05	35.12
Las Condes	21	2.45	37.57
Lo Barnechea	4	0.47	38.04
Lo Espejo	19	2.22	40.26
Lo Prado	9	1.05	41.31
Macul	16	1.87	43.17
Maipú	42	4.90	48.07
Ñuñoa	33	3.85	51.93
Pedro Aguirre Cerda	23	2.68	54.61
Peñalolén	25	2.92	57.53
Providencia	24	2.80	60.33
Pudahuel	38	4.43	64.76
Quilicura	21	2.45	67.21
Quinta Normal	29	3.38	70.60
Recoleta	27	3.15	73.75
Renca	16	1.87	75.61
San Joaquín	12	1.40	77.01
San Miguel	23	2.68	79.70
San Ramón	6	0.70	80.40
Vitacura	8	0.93	81.33
Puente Alto	99	11.55	92.88
Colina	3	0.35	93.23
Lampa	5	0.58	93.82
San Bernardo	42	4.90	98.72
Buin	2	0.23	98.95
Paine	1	0.12	99.07
Talagante	2	0.23	99.30
El Monte	1	0.12	99.42
Padre Hurtado	2	0.23	99.65
Peñaflor	3	0.35	100.00
Total	857	100.00	

C23. Lugar de la encuesta: Comuna

c23	Freq.	Percent	Cum.
Santiago	107	12.49	12.49
Cerrillos	38	4.43	16.92
Conchalí	20	2.33	19.25
Estación Central	65	7.58	26.84
Huechuraba	24	2.80	29.64
Independencia	20	2.33	31.97
La Cisterna	15	1.75	33.72
La Florida	65	7.58	41.31
La Reina	19	2.22	43.52
Las Condes	63	7.35	50.88
Lo Espejo	21	2.45	53.33
Ñuñoa	39	4.55	57.88
Pedro Aguirre Cerda	8	0.93	58.81
Peñalolén	14	1.63	60.44
Providencia	72	8.40	68.84
Pudahuel	20	2.33	71.18
Quilicura	21	2.45	73.63
Quinta Normal	22	2.57	76.20
Recoleta	38	4.43	80.63
San Miguel	40	4.67	85.30
San Ramón	26	3.03	88.33
Vitacura	32	3.73	92.07
Puente Alto	45	5.25	97.32
San Bernardo	23	2.68	100.00
Total	857	100.00	

C23_dir. Lugar de la encuesta: Dirección

c23_dir	Freq.	Percent	Cum.
Autopista Aconcagua esq. Av San Ignacio	21	2.45	2.45
Av. Ejercito Libertador esq. Patria Nue	22	2.57	5.02
Av. Las Nieves esq. Nemesio Vicuña	23	2.68	7.70
San Jose esq. Eyzaguirre	23	2.68	10.39
Metro Santa Rosa L4A	26	3.03	13.42
Plaza Antilen	11	1.28	14.70
Av. Vitacura esq. Embajador Doussinage	21	2.45	17.15
Agustinas esq. Morande	20	2.33	19.49
Metro Universidad Catolica L1	24	2.80	22.29
Cumming esq. Compañia de Jesus	21	2.45	24.74
Av. Santa Rosa esq. Amazonas	22	2.57	27.30
San Diego esq. Placer	20	2.33	29.64
Metro San Miguel L2	40	4.67	34.31
Metro Cerro Blanco L2	17	1.98	36.29
Bellavista esq. Pio Nono	21	2.45	38.74
Carrascal esq. Aviador Bleriot	22	2.57	41.31
San Pablo esq. Santa Victoria	20	2.33	43.64
Av. Vitacura esq. Nueva Tobalaba	24	2.80	46.44
Metro Salvador L1	23	2.68	49.12
Bellavista esq. Francisco Puelma	25	2.92	52.04
Las Perdices esq. Antupiren	14	1.63	53.68
Alhue esq. Quemchi	8	0.93	54.61
Av. Vicuña Mackenna esq. Victoria	20	2.33	56.94
Av. Grecia esq. Av. Jose Pedro Alessand	19	2.22	59.16
Av. Cardenal Raul Silva H. esq. Buenave	21	2.45	61.61
Av. Apoquindo esq. Puerta del Sol	23	2.68	64.29
Metro Alcantara L1	14	1.63	65.93
Av. Las Condes esq. Las Azaleas	26	3.03	68.96
Av. Alc. Fdo. Castillo V. esq. Valenzue	19	2.22	71.18
Av. La Florida / Santa Amalia	22	2.57	73.75
Metro Mirador L5	21	2.45	76.20
Metro Bellavista de la Florida L5	22	2.57	78.76
Av. El Parron esq. La Paz	15	1.75	80.51
Av.Independencia esq. Av. Santos Dumont	20	2.33	82.85
Av. Americo Vespucio esq. Los Libertado	24	2.80	85.65
Av. Ecuador esq. Enrique Kirberg	20	2.33	87.98
Metro Estacion Central L1	45	5.25	93.23
Av. Am. Vespucio N. esq. Av. Pdte. Edua	20	2.33	95.57
Av. Pedro Aguirre Cerda esq. Buzeta	14	1.63	97.20
Autopista Vespucio Sur esq. Camino a Lo	24	2.80	100.00
Total	857	100.00	

C24. Sitio de la encuesta

c24	Freq.	Percent	Cum.
Tienda de Barrio	49	5.72	5.72
Salida Centro Educativo	72	8.40	14.12
Centro de Ciudad	31	3.62	17.74
Centro Comercial	301	35.12	52.86
Cafetería	10	1.17	54.03
Otro. Especifique	394	45.97	100.00
Total	857	100.00	

c24_esp	Freq.	Percent	Cum.
	463	54.03	54.03
afuera de fonasa	1	0.12	54.14
afuera de metro	1	0.12	54.26
afuera hospital militar	1	0.12	54.38
automotora	2	0.23	54.61
banco	5	0.58	55.19
bar	3	0.35	55.54
calle	87	10.15	65.69
canal de television	17	1.98	67.68
clinica	1	0.12	67.79
comisaria	1	0.12	67.91
comunidad ecologica	10	1.17	69.08
consultorio	2	0.23	69.31
costado hospital militar	4	0.47	69.78
edificio profesional	1	0.12	69.89
entrada estadio israelita	2	0.23	70.13
entrada hospital fach	13	1.52	71.65
estacion de metro	9	1.05	72.70
estacion tren	3	0.35	73.05
feria	8	0.93	73.98
fonasa	10	1.17	75.15
hospital	49	5.72	80.86
hotel gran palace	2	0.23	81.10
metro	30	3.50	84.60
municipio	16	1.87	86.46
oficina de partes	2	0.23	86.70
paradero	29	3.38	90.08
parque	17	1.98	92.07
parroquia	3	0.35	92.42
patio de comida	2	0.23	92.65
plaza	44	5.13	97.78
plaza hospital militar	3	0.35	98.13
restaurante	1	0.12	98.25
salida hospital militar	5	0.58	98.83
salida metro	2	0.23	99.07
servicio médico legal	7	0.82	99.88
supermercado	1	0.12	100.00
Total	857	100.00	
